# The genome of *Hibiscus hamabo* reveals its adaptation to saline and waterlogged habitat

**DOI:** 10.1093/hr/uhac067

**Published:** 2022-03-23

**Authors:** Zhiquan Wang, Jia-Yu Xue, Shuai-Ya Hu, Fengjiao Zhang, Ranran Yu, Dijun Chen, Yves Van de Peer, Jiafu Jiang, Aiping Song, Longjie Ni, Jianfeng Hua, Zhiguo Lu, Chaoguang Yu, Yunlong Yin, Chunsun Gu

**Affiliations:** 1 Institute of Botany, Jiangsu Province and Chinese Academy of Sciences, Nanjing, 210014, China; 2 College of Horticulture, Academy for Advanced Interdisciplinary Studies, Nanjing Agricultural University, Nanjing 210095, China; 3 State Key Laboratory of Pharmaceutical Biotechnology, School of Life Sciences, Nanjing University, Nanjing 210023, China; 4 Department of Plant Biotechnology and Bioinformatics, Ghent University, VIB-UGent Center for Plant Systems Biology, B-9052 Ghent, Belgium; 5 Department of Biochemistry, Genetics and Microbiology, University of Pretoria, Pretoria 0028, South Africa; 6 College of Horticulture, Nanjing Agricultural University, Nanjing 210095, China; 7 College of Forest Sciences, Nanjing Forestry University, Nanjing, 210037, China; 8 Jiangsu Key Laboratory for the Research and Utilization of Plant Resources, Jiangsu Utilization of Agricultural Germplasm, Nanjing, 210014, China

## Abstract

*Hibiscus hamabo* is a semi-mangrove species with strong tolerance to salt and waterlogging stress. However, the molecular basis and mechanisms that underlie this strong adaptability to harsh environments remain poorly understood. Here, we assembled a high-quality, chromosome-level genome of this semi-mangrove plant and analyzed its transcriptome under different stress treatments to reveal regulatory responses and mechanisms. Our analyses suggested that *H. hamabo* has undergone two recent successive polyploidy events, a whole-genome duplication followed by a whole-genome triplication, resulting in an unusually large gene number (107 309 genes). Comparison of the *H. hamabo* genome with that of its close relative *Hibiscus cannabinus*, which has not experienced a recent WGT, indicated that genes associated with high stress resistance have been preferentially preserved in the *H. hamabo* genome, suggesting an underlying association between polyploidy and stronger stress resistance. Transcriptomic data indicated that genes in the roots and leaves responded differently to stress. In roots, genes that regulate ion channels involved in biosynthetic and metabolic processes responded quickly to adjust the ion concentration and provide metabolic products to protect root cells, whereas no such rapid response was observed from genes in leaves. Using co-expression networks, potential stress resistance genes were identified for use in future functional investigations. The genome sequence, along with several transcriptome datasets, provide insights into genome evolution and the mechanism of salt and waterlogging tolerance in *H. hamabo*, suggesting the importance of polyploidization for environmental adaptation.

## Introduction

*Hibiscus hamabo* Siebold & Zuccarini, from the Malvaceae family, is a deciduous tree that can reach 5 m in height [1]. *H. hamabo*, which is endangered, is an appealing ornamental woody plant with obvious seasonal variations; the plant blooms in summer, presenting light green to light lemon-yellow flowers, and its leaves turn red in fall [1]. The plant is native to Asia and mainly distributed in the coastal areas of China, Korea, and Japan, as well as India and some Pacific islands [1, 2]. The distribution of *H. hamabo* suggests that this species is not adapted to cold zones but prefers the warm regions of coastal areas in the northwestern Pacific. It is known as a semi-mangrove species with long-floating seeds [1]. Plants from the *Hibiscus* genus show complex ploidy and various chromosomal numbers. Diploids, tetraploids, hexaploids, and octoploids can all be found in the genus, with various chromosomal numbers, ranging from *Hibiscus citrinus* (2n = 22) [3] to *Hibiscus maculatus* (2n = 180). *H. hamabo* is known to contain 92 (2n = 92) chromosomes, but it remains unclear whether it is a diploid species (x = 46) or a diploidized tetraploid species (x = 23) [1]. One phylogenetic study suggested that *H. hamabo* is related to *Hibiscus tiliaceus* (2n = 96) and *Hibiscus glaber* (2n = 82) [4], whereas another study indicated a close relationship between *H. hamabo* and *Hibiscus macrophyllus* [5]. However, both studies were based on a very restricted number of markers and limited taxon sampling, and the relationship between *H. hamabo* and other *Hibiscus* plants therefore remains ambiguous. *H. hamabo* may have independently appeared outside the normal range inhabited by *H. tiliaceus* through the long-distance floating of seeds and/or adaptation to new circumstances [6].

*H. hamabo* is a semi-mangrove plant that can survive in tidal saline soil and under immersion [7, 8]. How it adapts to such stressful environmental conditions is an intriguing question. Numerous studies have investigated the molecular mechanisms that underlie plant tolerance to salt and waterlogging. Under salt stress, the salt overly sensitive (SOS) pathway, which is conserved in plants, is induced within 2 h to export Na^+^ out of cells; SOS2 kinase and the SOS1 Na^+^ antiporter are then induced to increase Na^+^ efflux [9]. Meanwhile, the SOS pathway coordinates with high-affinity K^+^ transporter (*HKT1*) to regulate Na^+^/K^+^ homeostasis in plant cells, reduce the accumulation of Na^+^ in stem tissues, and protect leaves from poisoning caused by Na^+^ [10]. The vacuolar partitioning of Na^+^, which is regulated by a Na^+^/H^+^ exchanger (*NHX*), is the main adaption mechanism used by both halophytes and glycophytes to reduce ion toxicity in the cytoplasm [10]. In addition, ABA-dependent and independent SUCROSE nonfermenting1-related PROTEIN KINASE2 genes (*SnRKs*) in *Arabidopsis* were found to play key roles in regulation at both the transcriptional and post-transcriptional levels under salt stress, mainly through the targeted regulation and phosphorylation of downstream components [11]. When oxygen is limited because of flooding, anaerobic respiration, which is related to glycolysis and fermentation, is the primary method used by plants to produce energy [12, 13]. Anaerobic proteins (ANPs) such as alcohol dehydrogenase (ADH), pyruvate decarboxylase (PDC), and lactate dehydrogenase (LDH), which play major roles in anaerobic respiration pathways, have been identified as key functional components in many plant species, including *Arabidopsis*, rice, and maize [12]. Moreover, as the hormone ethylene is critical for coping with waterlogging [13], Group VII ethylene-responsive element binding factors (ERFs) in *Arabidopsis* are key regulators of genes related to hypoxia and the waterlogging stress response [14].

Recent studies have shown that global warming may result in rising sea levels, which will increase the stresses endured by coastal plants [7]. As a shelterbelt tree species along the coasts, *H. hamabo* is a good candidate for studying responses and regulatory mechanisms under stress, which are crucial for using this species in the ecological greening of coastal vegetation systems. Some attempts have been made to understand the mechanisms of stress adaptation in *H. hamabo*, but the absence of genomic data has hindered systematic investigations to date [2, 15]. Here, we report the assembly of the *H. hamabo* genome at the chromosome level using a combination of PacBio, Hi-C, and Illumina sequencing technology. Using multiple transcriptomic datasets obtained under different treatments and at various time points, we reveal the adaptive mechanisms of *H. hamabo* to saline and waterlogged environments. This genome should serve as a useful resource, helping the investigation of semi-mangrove plants and promoting the development and utilization of woody coastal plants.

**Table 1:** Data from the *H. hamabo* assembly

Statistic	*H. hamabo*
Assembly size (bp)	1718,145,230
Number of scaffolds	1915
Scaffold N50 size (bp)	36 349 750
Longest scaffold (bp)	64 458 935
Number of contigs	4358
Contig N50 size (bp)	1 645 376
Longest contig (bp)	9 364 180
Number of genes	107 309
BUSCO score	98.1%
GC (%)	34.90

## Results

### Genome ploidy, sequencing, assembly, and annotation

**Figure 1:**
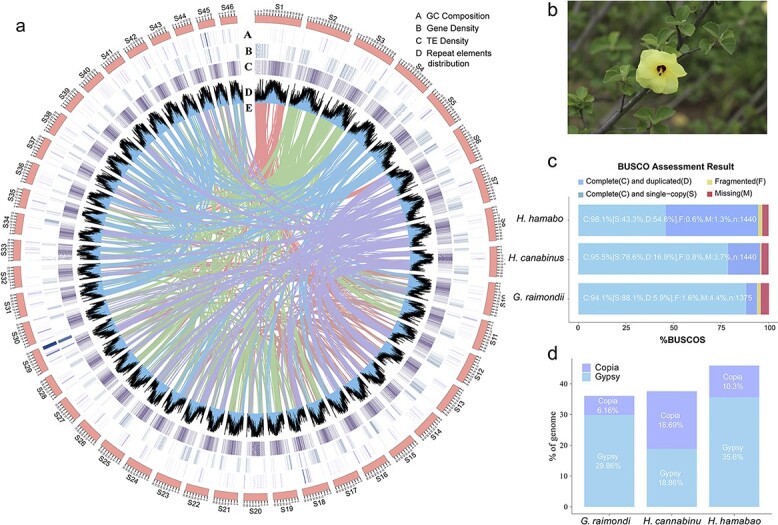
**Overview of the *Hibiscus hamabo* genome (a)** Circos plot displaying the general characteristics of the *H. hamabo* genome. Tracks from outside to inside show GC composition (A), gene density (B), TE density (C), repetitive element distribution (D), and colored bands representing colinear blocks derived from the most recent WGD (whole-genome duplication) event (E). The non-overlapping sliding window for density plots is 500 kb. **(b)** The flower of *H. hamabo*. **(c)** The BUSCO assessment results of the *H. hamabo* genome compared with those of kenaf (*Hibiscus cannabinus*) and cotton (*G. raimondii*). **(d)** LTR retrotransposons in *H. hamabo* compared with kenaf and cotton.

An investigation of ploidy revealed that *H. hamabo* is a diploid species with 46 pairs of chromosomes (2n = 2x = 92) (Fig. S2a), and k-mer analysis of Illumina data indicated that its genome size was 1712 Mb. The results of a genome survey indicated that the *H. hamabo* genome had a low heterozygosity of 0.06% (Fig. S2b, S2c). To obtain a high-quality genome, we adopted an elaborate strategy for genome assembly. We integrated paired-end reads from Illumina (226 G), single-molecule long reads from PacBio (N50 = 7.7 kb, 138 G), and Hi-C sequencing data (161 G) to perform the following steps. First, an initial assembly with a size of 1717 Mb (contig N50 = 1.6 Mb, 4358 contigs) was constructed with the PacBio long reads (80×). Then, Illumina reads (133×) were used to correct the assembled sequences from the previous step. Lastly, we used Hi-C paired-end reads (95×) to produce a final assembly of 1718 Mb (scaffold N50 = 36.3 Mb) in 1915 scaffolds (Table 1, Table S4), similar to the results of the previous assessment. Forty-six of the longest pseudomolecules, accounting for 98.4% (1690 Mb) of the assembly, were selected to correspond to the 46 chromosomes of the haplotype genome of *H. hamabo* (Fig. 1a, Fig. S5). A genome-wide interaction heatmap shows the grouping and ordering results obtained using the Hi-C data (Fig. S6). Altogether, 52.9% (909 Mb) of the assembly was annotated as repetitive sequences, with long terminal repeat (LTR) elements forming the predominant component (47.4%), including 35.6% Gypsy and 10.3% Copia retro-elements (Fig. 1c, Tables S9, S10). We integrated *ab initio*, homology-based, and RNA-assisted gene prediction methods to obtain gene models for the *H. hamabo* genome. We predicted 107 309 protein-coding genes with an average length of 1009 bp and an average of 4.42 exons per gene. RNA sequences obtained using the PacBio and Illumina sequencing platforms from eight different tissues and organs were used to provide transcriptional evidence to support the gene annotations. Of all the protein-coding genes, 90.4% were assigned putative functional annotations, among which 35.7% (38297), 14.2% (15242), and 60.2% (64637) were from the MF (molecular function), CC (cellular component), and BP (biological process) categories, respectively (Table S13).

To obtain gene models for the *H. hamabo* genome, we adopted three different strategies for gene prediction, as mentioned above (see **Methods**). Our annotation captured 98.1% of the Embryophyta BUSCO gene set (odb10, complete and duplicated complete), higher than that in other published Malvaceae genomes, including *Hibiscus cannabinus* (95.5%) and *Gossypium raimondii* (97.4%) (Fig. 1b), indicating the high quality of the *H. hamabo* genome in terms of annotation completeness. Given the high percentage of transposable elements (TEs), we further assessed genome quality by calculating the LTR Assembly Index (LAI) and obtained a final LAI value of 9.89, very close to the “reference” level according to the criteria of Ou et al [16]. We also identified 2743 transfer RNAs, 3936 ribosomal RNAs, 583 small nuclear RNAs (snRNAs), and 439 microRNAs (miRNAs) (Table S14).

### Phylogenetic relationships, divergence times, and gene family evolution

**Figure 2:**
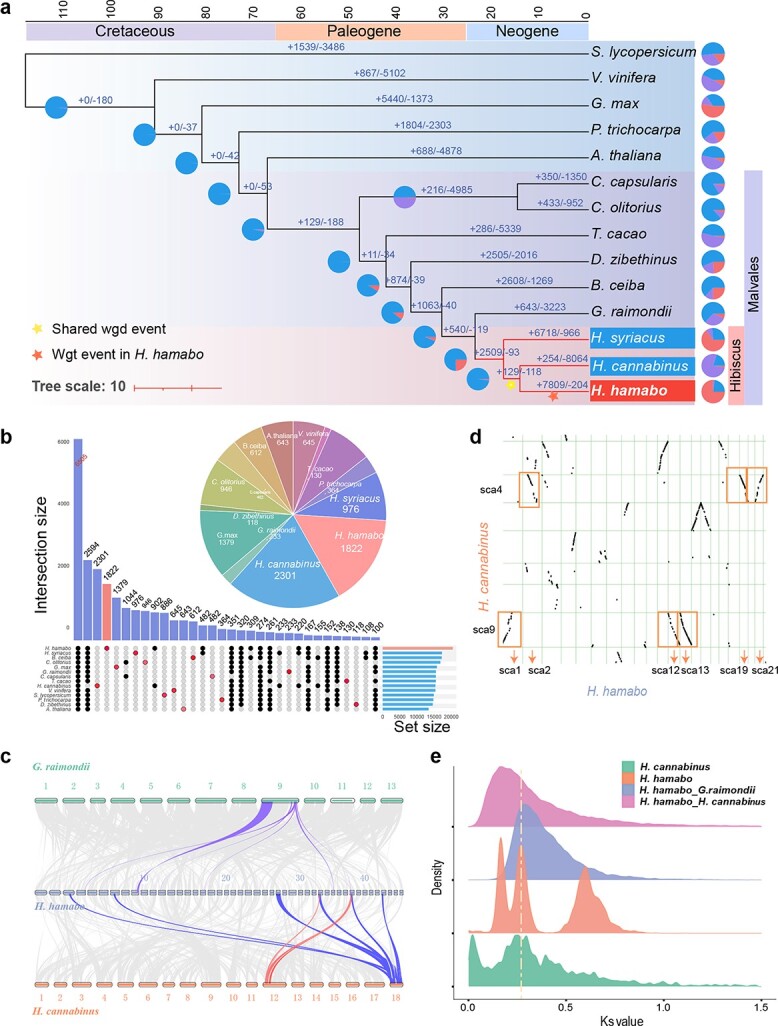
**Analysis of evolution and gene families in 14 species (a)** Phylogenetic tree of 14 species; the scale at the top represents the divergence time, and the pie charts represent the expansion and contraction of gene families, with red indicating expansion and purple indicating contraction. The yellow and red stars indicate a shared WGD event and a WGT event in *H. hamabo*, respectively. **(b)** Upset plots for 14 species; the pie chart shows the number of unique families for each species. **(c)** Intergenome collinearity plot shows a pattern of 1:6 for *G. raimondi* and *H. hamabo* and 2:6 for *Hibiscus cannabinus* and *H. hamabo.***(d)** Intergenome collinearity dot plot shows a pattern of 1:3 for *H. cannabinus* and *H. hamabo.***(e)** Distribution of overall synonymous substitution levels (Ks) for paralogs found in syntenic blocks of *H. cannabinus* and *H. hamabo* and for orthologs between *H. hamabo* and *H. cannabinus* and between *H. hamabo* and *G. raimondii*. The yellow dotted line indicates a recent WGD event shared by *H. hamabo* and *H. cannabinus.*

Phylogenetic analysis was performed using 385 concatenated low-copy nuclear genes from 14 angiosperm species, including four from the Malvaceae. The phylogenetic inference recovered monophyly of the genus *Hibiscus* and the family Malvaceae. *H. hamabo* was resolved as the sister to *H. cannabinus*, and *Hibiscus syriacus* was placed as the sister to a lineage composed of *H. hamabo* and *H. cannabinus*, suggesting a closer relationship between *H. hamabo* and *H. cannabinus* among the three species. Molecular dating based on this topology suggested that *Hibiscus* originated about 25 million years ago (Mya), and the speciation of *H. hamabo* was inferred to have occurred approximately 15 Mya with a 95% confidence interval (Fig 2a, Fig S12).

The 107 309 protein-coding genes of *H. hamabo* were classified into 35 408 families, including over 6000 families shared with the other 13 species and 1822 unique families (Fig. 2b). Gene expansion and contraction analysis indicated that *H. hamabo* has undergone more expansion (7809) than contraction (204) of gene families since its divergence from *H. cannabinus* (Fig. 2a). Gene ontology (GO) enrichment analysis showed that 2004 significantly expanded (p < 0.05) gene families in *H. hamabo* were most enriched in the BP terms “cellular response to lipid”, “flower development”, “leaf development”, “plant organ morphogenesis”, “response to water”, “response to auxin” and “regulation of hormone levels” and the MF terms “identical protein binding”, “DNA-binding transcription factor activity”, and “*cis*-regulatory region sequence-specific DNA binding” (Fig. S11). GO analysis of the 1822 unique gene families showed that they were most enriched in the BP terms “response to endogenous stimulus”, “response to hormone”, “dephosphorylation”, “cell redox homeostasis”, “anatomical structure development”, and “response to auxin” and the MF terms “phosphoprotein phosphatase activity”, “ubiquitin-protein transferase activity”, “protein serine/threonine phosphatase activity”, “protein disulfide oxidoreductase activity” and “protein kinase regulator activity” (Fig. S13). These results suggested an important contribution of expanded and unique genes to functions in environmental adaptability to salt and water, especially with regard to signal transduction.

### Polyploidy events in *H. hamabo*

Intra-genomic synteny analysis of *H. hamabo* indicated that there were a number of syntenic blocks (Fig. 1A) and a higher density of duplications compared with *H. cannabinus* [17], suggesting an unusual history of polyploidization in this species. We first calculated the number of synonymous substitutions per synonymous site (Ks) for all the paralogs (paranome) of *H. hamabo* and found only a single signature peak between 0.15–0.2 (Fig. S15a). After rate adjustment, two peaks at Ks = 0.21 (mode a) and Ks = 0.56 (mode b) emerged, suggesting that two polyploidy events had occurred in *H. hamabo*. As *H. hamabo*’s speciation peaks with *H. cannabinus* and *H. syriacus* were located at 0.12 and 0.18 (Fig. S15b), it was clear that these two polyploidy events took place in the common ancestor of all *Hibiscus* plants. To precisely locate the position of the whole-genome duplication (WGD) associated with Ks = 0.56, the paralogs of *G. raimondii* and the reciprocal best hit (RBH) orthologs were extracted. The Ks distribution showed a WGD peak for *G. raimondii* at Ks ≈ 0.5 (Fig. S15c), consistent with that of *H. hamabo* (mode b, Ks ≈ 0.56), and a speciation peak at Ks ≈ 0.3 between *H. hamabo* and *G. raimondii* (Fig. S15d), suggesting that there was a shared WGD event between *H. hamabo* and *G. raimondii* before speciation*.* According to these findings, *H. hamabo* and *H. cannabinus* should have experienced the same polyploidy events. However, we performed subsequent inter-genomic synteny analyses between the two species using several different methods, and interestingly, all of the results showed a 6:2 or 3:1 pattern between them (Fig. 2c, 2d, Fig. S16), indicating that *H. hamabo* may have undergone an extra independent whole genome triplication (WGT) event after its split from the common ancestor of *H. hamabo* and *H. cannabinus*. With respect to the Ks peak at 0.21, if there had been a species-specific WGT in *H. hamabo*, the WGT peak would probably be covered by or integrated into this 0.21 peak. To more precisely distinguish the Ks value for the peak of triplicated genes from the peaks of other WGD events, the anchor-pair genes retained in all of the syntenic blocks were extracted. Then, another parameter—the Ks median value of every syntenic block—was used for subsequent analyses [18]. As a result, two very close but independent peaks located at Ks values of 0.1–0.2 and 0.2–0.3 were identified, representing two recent polyploidy events (Fig. 2e, Fig. S17b). Dot plots of the syntenic blocks representing the most recent WGT event showed that the pattern for each block had three syntenic counterparts. Therefore, using *H. cannabinus* as a reference, we discovered clues to the “hidden” WGT event and successfully distinguished it from another WGD event that occurred not long before. This recent WGT also explains the large number (over 100 000) of protein-coding genes in *H. hamabo*. In summary, *H. hamabo* and *H. cannabinus* shared two polyploidy events before their speciation, the earliest of which was also shared with *G. raimondii*, and shortly after the divergence of *H. hamabo*, a WGT event took place in this species (Fig. 2e).

### Transcriptomic responses and regulatory networks that are critical to survival in a tidal saline soil and waterlogged environment

**Figure 3:**
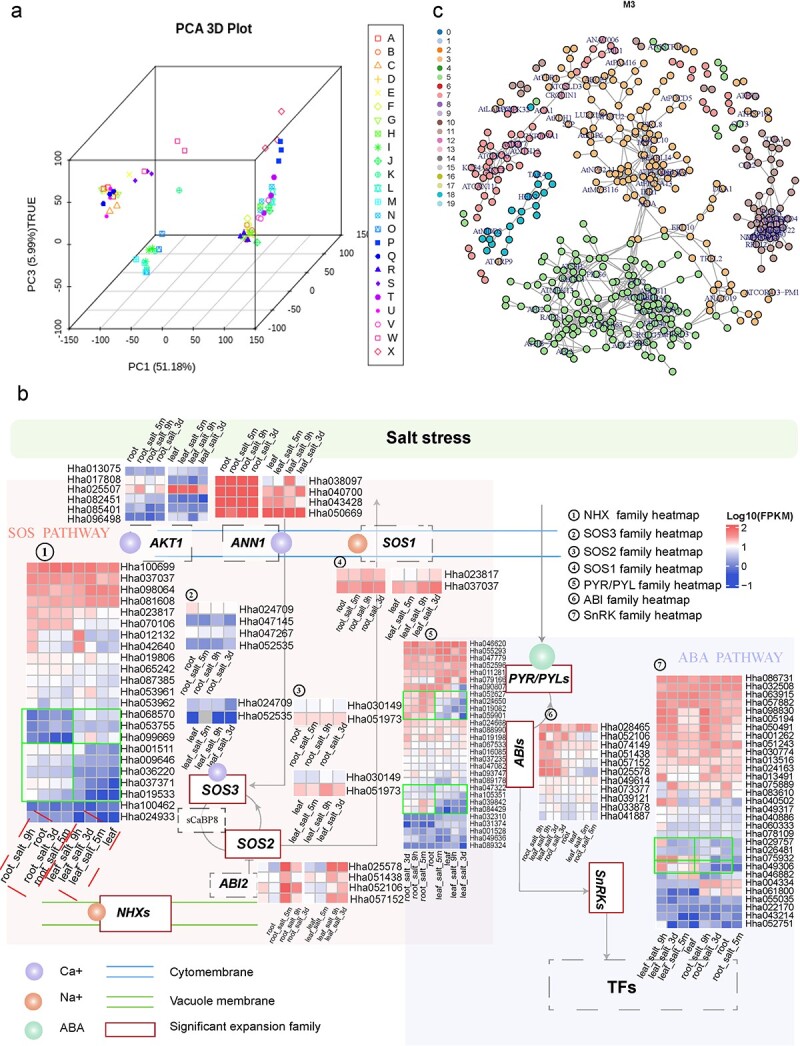
**Transcriptome-based analysis of stress-resistance mechanisms in *H. hamabo* (a)** Principal component analysis of transcriptome data from different samples. A–X represent different sample groups. See Table S15 for information about sample groupings. **(b)** SOS (Salt overly sensitive) and ABA (abscisic acid) pathway responses to salt stress in *H. hamabo*. ANN1 (annexin1) and AKT1 are a Ca^2+^ and K^+^ transporter, respectively. Under salt stress, the SOS pathway is activated by a calcium signal, and SCaBP8 is phosphorylated by SOS2, which may disassociate from AKT1 to promote the concentration of K^+^. The SOS signaling pathway comprises SOS3, SCaBP8, SOS2, and SOS1 and is important for the regulation of Na^+^/K^+^ ion homeostasis. ABI2 (an ABA-responsive gene) negatively regulates the kinase activity of SOS2. NHXs are vacuolar K^+^/H^+^ exchangers. In the ABA pathway, the accumulation of ABA activates SnRK2s via the PYR/PYLs-PP2Cs/ABIs-mediated regulatory module. The heatmap shows the differential gene expression patterns in leaves and roots. **(c)** Correlation networks under salt and waterlogging stress. The numbers 0–19 indicate 20 modules related to salt stress identified from the transcriptomic data.

To explore the regulatory mechanisms used by *H. hamabo* to survive under salt and/or waterlogging stress, we sequenced the transcriptomes of its roots and leaves under different treatments: saline soil (S), waterlogging (W), and salt + waterlogging (SW) at 5 min, 9 h, and 3 d after treatments (Table S15). The transcriptomic data showed that the roots and leaves had distinct gene expression profiles, suggesting the presence of different responses and regulatory events in roots and leaves after salt and/or waterlogging treatments (Fig. 3a). To analyze the expression of stress-related genes, all differentially expressed genes (DEGs) in the leaves and roots were classified into 20 clusters according to their expression patterns (Figs. S19, S20).

Maintaining a proper Na^+^/K^+^ ratio in the cytoplasm is an important adaptive characteristic of salt-tolerant plants to the presence of excess ions [19]. In *H. hamabo* roots, *SOS2* (Hha031532) and *AKT1* (Hha025507) were quickly (5 min) upregulated under S and SW treatments compared with the control (CK), indicating their sensitivity to stimuli and their important roles in short-term responses to the changing Na^+^/K^+^ ratio. Based on the clustering results, *SOS2* and *AKT1* fell into Subclusters 11 and 18 (Figs. S20, S21). The leaf transcriptomes from 5 min after treatment showed no significant changes, indicating a slower response than that seen in the roots. However, when plants were exposed to saline conditions for 9 h and 3 d, *H. hamabo NHX2* (Hha100699), encoding a major tonoplast-localized NHX isoform, was induced in leaves to regulate the ion balance. It can influence the Na^+^/K^+^ ratio by adjusting the vacuolar absorption of K^+^, thereby regulating cell turgor and stomatal function [20]. The *NHX* gene was grouped into Subcluster 14, which was also enriched in genes related to transcription factor activity, suggesting a role for transcription factors in leaves (Figs. S19, S22). Aside from its involvement in calcium signaling, Na^+^ influx also induces an increase in the production of reactive oxygen species (ROS) [11]. *H. hamabo CAT1* (catalase 1, Hha081091 in Subcluster 17), which encodes an antioxidant enzyme, was upregulated in root samples 9 h and 3 d after S and SW treatments, and this is considered to be a reasonable regulatory response to long-term salt stress [21]. In the same subcluster, genes related to proline biosynthetic and metabolic processes, response to gibberellin, and the gibberellin-mediated signaling pathway were also enriched, indicating that solutes and phytohormones also participated in medium- and long-term adjustments under salt stress (Fig. S23). The accumulation of ROS under salt stress subsequently triggers MAP kinase (MAPK) cascades [19], which have been shown to participate in salt-stress signaling responses in *Arabidopsi*s [22, 23] and were also found to respond in *H. hamabo* roots under S and SW conditions. *MPK3* (Hha060729, Hha033359, and Hha018260) and *MPK4* (Hha095461) were in Subcluster 18, and *MPK6* (Hha011239) was in Subcluster 17. SnRK kinase, a key transcriptional and post-transcriptional regulator under salt stress, has a variety of downstream phosphorylation targets that mediate many processes, such as ion transport, ROS production, and gene transcription [11]. In *H. hamabo* roots, *SnRK*s (Hha057882 and Hha063915) were also found in Subcluster 17, together with antioxidant enzymes and MAPK members, demonstrating that ABA may activate ROS signaling and MAPK cascades through *SnRK*s to regulate the growth of the root system. Another ABA receptor, *GCR2* (G-protein-coupled receptor 2, Hha009184) in Subcluster 1, was upregulated in leaf samples treated with S and SW for 9 h and 3d, suggesting that it may be involved in the regulation of stomatal closure and senescence through ABA signal transduction in leaf tissue [24]. Genes related to the metabolism of energy-storing substances and the gibberellin-mediated signaling pathway were also significantly enriched in Subcluster 1 (Fig. S24), suggesting that *H. hamabo* may also regulate hormone signal transduction and energy metabolism in leaves to defend against stress injury. In summary, genes enriched in root Subclusters 11 and 18 may have regulatory roles in the short-term root salt stress response, whereas Subcluster 17 genes seem to participate more in long-term regulation. The leaves did not appear to respond very quickly to the stress treatments, but genes in Subclusters 1 and 14 may be relevant in the long term.

Waterlogging can reduce gas diffusion, resulting in limited oxygen availability, a condition commonly referred to as hypoxia [25]. To overcome this unfavorable condition, endogenous ethylene accumulates in submerged plant tissues to activate several adaptive signal transduction pathways [25]. In *H. hamabo*, *ADH1* (alcohol dehydrogenase) (Hha098758, Hha081929, Hha098733, Hha005263, Hha005264, and Hha097945) and *PDC1* (pyruvate decarboxylase) (Hha038280) were found in Subcluster 9 and were strongly upregulated in W- and SW-treated root samples at all time points to ensure the provision of energy supply under stress (Fig. S25). *RAP2.2* (Hha038572, Hha021850, and Hha050179) was also found in Subcluster 9 and was strongly upregulated in W- and SW-treated roots at 5 min to promote the upregulation of *ADH* and *PDC*. In Subcluster 9, genes annotated with the terms small molecule metabolic process, single-organism metabolic process, purine-containing compound metabolic process, purine ribonucleotide metabolic process and others were significantly enriched, suggesting that *H. hamabo* can deploy metabolic systems to clean up damaged nucleotide molecules in response to environmental stress.

Finally, we compared the expression patterns of salt-resistance pathway genes under SW treatment (Fig. 3b). Because of the WGT event, all related genes are expanded in *H. hamabo*, and some duplicates show different expression patterns in roots and leaves, suggesting further functional divergence among duplicated copies. For example, *AKT1* has expanded into six duplicates, with one copy, Hha025507, exhibiting higher expression in leaves than roots, and another, Hha017808, showing the opposite pattern. The *NHX* family also showed a reciprocal expression pattern, with three members (Hha068570, Hha53755, and Hha099669) more highly expressed in leaves than in roots and five members (Hha001511, Hha009646, Hha036220, Hha037371 and Hha019533) more highly expressed in roots. *ANN1* has expanded into four duplicates, almost all of which are extremely highly expressed in roots but less so in leaves. Among six duplicates of *AKT1*, one gene, Hha025507, shows much higher expression in both roots and leaves than the other five members. Similarly, four members of the *NHX* family (Hha100699, Hha037037, Hha098064, and Hha081608) show higher levels of expression than the other members. This scenario may reflect the functional redundancy of the recent duplicates.

Because many of the identified genes were involved in different pathways and associated with regulation under stress, it is likely that they form a network to co-regulate responses under stress, especially salt stress. Based on the transcriptomic data, we performed a weighted gene co-expression network analysis (WGCNA) to identify genes potentially involved in the salt-stress response network. Based on their expression patterns, the genes expressed under SW treatment were classified into 20 modules (Fig. S26), among which Modules 3, 5, 7, 11, and 18 contained known functional salt-resistance genes (e.g. *AKT1*, *SOS2*, *SOS3*, and *ABI2*) (Fig. 3c). Thus, the genes in these modules may play a role in the salt stress regulatory network. The well-known salt-stress genes *SOS2* and *SOS3* were classified into Modules 11 and 18, respectively, and these two modules contained 125 and 40 genes, respectively (Table S16), providing clues to assist in the investigation of the plant salt-stress regulatory network. Future functional characterization studies should investigate the functions of these candidate genes.

**Figure 4:**
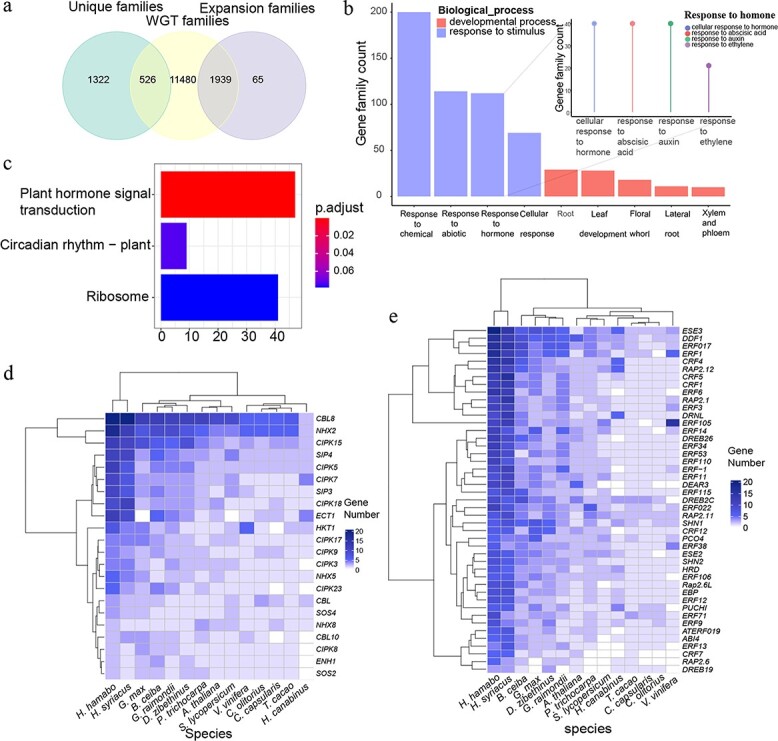
**Analysis of “WGT-expansion” gene families (a)** Venn diagram showing the relationships among unique families, WGT families, and expanded families in the *H. hamabo* genome**. (b)** Summary of the GO enrichment results for the “WGT-expansion” genes; see Fig. S18 for more details. The diagram represents the enriched first-level GO terms “developmental process” and “response to stimulus”, and the lollipop chart shows secondary terms associated with “response to hormone”. **(c)** KEGG enrichment result for the “WGT-expansion” genes. **(d)** Expansion of genes involved in the salt stress signal transduction pathway. The different colors represent the copy numbers of each gene. **(e)** Expansion of genes involved in the response to ethylene. The different colors represent the copy numbers of each gene.

### Polyploidy in *hibiscus* is likely to be associated with stress resistance

As a semi-mangrove plant, *H. hamabo* resides in a very harsh habitat compared with other *Hibiscus* plants, and its strong tolerance to stress guarantees its survival in such environments. Because many studies have pointed out that polyploids have stronger resistance to stressful environments [26–29], we wanted to investigate whether there was an association between the specific recent WGT of *H. hamabo* and its adaptation to stress. The unique gene set, significantly expanded gene set, and WGT gene set were extracted for comparison and analyses. We found that almost all of the significantly expanded genes (over 96.7%) and over a quarter of the unique genes (28.4%) belonged to the WGT gene set, suggesting that the recent WGT event contributed greatly to gene family expansion (Fig. 4a). In total, 1939 “WGT-expansion” gene families were used in a GO enrichment analysis, and these genes were mainly enriched in “response to chemical”, “response to abiotic stimulus”, “response to hormone” and “cellular response” from the “response to stimulus” category and were also significantly enriched in “developmental process”, including “leaf development”, “floral whorl”, “lateral root”, and “xylem and phloem” (Fig. 4b, Fig. S18). The genes that responded to hormones were mainly enriched in GO terms related to ABA, auxin, and ethylene. The ABA pathway is important for plant stress resistance [30], auxin can enhance plant waterlogging tolerance by regulating lateral root initiation and development [11, 31], and the ethylene signal transduction pathway plays a key role in the response to waterlogging [30, 32]. These genes were additionally mapped to Kyoto Encyclopedia of Genes and Genomes (KEGG) pathways, and they were enriched (p < 0.05) in the “plant hormone signal transduction”, “circadian rhythm-plant” and “ribosome” categories (Fig. 4c). These results suggested that the *H. hamabo* WGT played a critical role in its adaptability to survival in a high-saline and waterlogged environment. We also annotated and identified *SOS* and ethylene-response gene families/subfamilies, which regulate salt [9] and waterlogging stress [12, 13, 32, 33], respectively. *CIPKs*, *CBLs*, and *NHXs*, which are supposed to respond to salt stress, were all expanded in *H. hamabo* compared with other *Hibiscus* species. Likewise, all ethylene-response gene families were also significantly expanded. Interestingly, *H. syriacus*, a species with medium salt tolerance, had a pattern of gene family expansion similar to that of *H. hamabo* (Fig. 4d, 4e). *H. syriacus* is also likely to have undergone an independent WGD event, implying an underlying mechanistic link between stronger adaptive ability and polyploidy events.

## Discussion

The genus *Hibiscus* comprises over 200 species that are widely distributed in the tropics and subtropics. *H. hamabo* was originally discovered on the eastern coasts and islands of China and is one of the few species in this genus that can survive in saline and waterlogged habitats. In this study, we sequenced the first semi-mangrove plant genome using a combination of Illumina, PacBio, and Hi-C technology and produced a chromosome-level assembly. The diversity of chromosome numbers in *Hibiscus* suggests very complicated and distinct evolutionary histories for these species, and polyploidy events are likely to have played important roles in this process. We identified multiple rounds of polyploidy events in three sequenced *Hibiscus* species, including WGD and WGT, with some events shared by all species and some specific to particular species. Polyploidy events inevitably doubled the chromosome number, whereas post-fragmentation is likely to have exacerbated genome reshuffling, leading to more complex chromosome numbers in this genus. There may also have been hybridization events within and outside the genus, as *Hibiscus* species do not always form a monophyletic group [6]. Ambiguity in the phylogenetic relationships between *Hibiscus* and closely related taxa remains and may be associated with frequent polyploidy and possible hybridization events. To achieve a well-resolved phylogeny, broader taxon sampling and genomic markers would be necessary. The identification of a recent WGT helped us to interpret the evolution of the *H. hamabo* genome and explains the >100 000 protein-coding genes in the species. The identification of this recent WGT also demonstrates the importance of references for comparative analysis. Without *H. cannabinus*, the evidence for the WGT in *H. hamabo* would have been difficult to verify. The complex evolutionary history of *Hibiscus* species subsequently shaped their diverse morphological characteristics. The *H. syriacus* genome experienced one individual WGD event, and the preferentially retained genes played a critical role in its unique floral morphology [34]. Similarly, a recent WGD took place in the *H. cannabinus* genome*,* and the reduplicated gene families are involved in bast fiber formation [17]. Our research revealed one WGD shared with *H. cannabinus* and another WGT event that occurred after speciation. This high-quality genome will lay the foundation for further research on *Hibiscus* evolution and the molecular mechanisms of its environmental adaptation.

The WGT also generated more copies of stress-resistance genes, including genes involved in salt and waterlogging resistance that contribute to the survival of this species in a harsh habitat. Polyploids, especially recently formed polyploids, have broad ecological tolerance [29]. WGDs provide abundant genetic resources to enable plants to cope with environmental changes. After a WGD (or WGT), neofunctionalization and subfunctionalization create novel genes, leading to expanded functional diversity and more complex regulatory networks [35]. In *H. hamabo*, >95% of the significantly expanded gene families belonged to recent WGT-derived genes (Fig. 3A), including multiple stress resistance-related genes, such as SOS pathway and ethylene-related genes (Fig. 4d, 4e). Stress resistance pathways form interrelated regulatory networks. A change in gene number in one pathway inevitably influences other related pathways, and gene dosages must be balanced to enable all of the pathways and networks to work properly [36, 37] (Fig. 3c). For example, salt stress activates the SOS pathway and causes the accumulation of ROS, which subsequently triggers MAPK cascades [19]. These cascades function in ROS homeostasis as central regulators of ROS detoxification and also contribute to ion homeostasis by promoting SOS1 phosphorylation [19].

As a semi-mangrove species with strong resistance to salt, *H. hamabo* may have many mechanisms of salt tolerance, such as salt excretion, salt rejection, and salt dilution. Furthermore, *H. hamabo* has a series of structures adapted to the saline environment, such as stellate hairs and crystal cells [38]. The selective absorption of Na^+^ and K^+^ and a higher proportion of Na^+^ accumulation in the roots relative to the whole plant are among the salt exclusion characteristics of *H. hamabo* [39]. Because it is a salt-dilution halophyte, osmotic regulation and ion compartmentation may also be physiological adaptations of *H. hamabo* to salt stress [40]*.* As the transcriptomic data suggest, roots respond very quickly to stress, adjusting ion concentrations in and outside plant cells, as well as inhibiting the production of damaging compounds. In an ion concentration test, the Na^+^/K^+^ ratio was significantly higher in the 9-h S group, the 24-h SW group, and the 3-d SW group than in the control group, and it was significantly higher in the root system than in the leaves (Fig. S27), suggesting that *H. hamabo* may be a kind of K^+^-preferring and Na^+^-refusing halophyte. *H. hamabo* not only is an exclusion-type halophyte that exhibits preferential absorption of K^+^ over Na^+^ through the roles of genes like *AKT* and *SOS* but also exhibits the mechanism of salt dilution through osmotic regulation and ion zone isolation. Leaves take longer to respond, triggering transcription factors and auxin biosynthesis and regulation. Leaves containing auxin show the greatest capacity for *de novo* auxin synthesis and commonly show increased resistance to stress; in particular, auxin with stress-induced ROS signals has been reported to correlate plant development with plant responses to environmental changes [41]. With further investigation of the large numbers of genes identified in the *H. hamabo* genome, the complex mechanisms of salt and waterlogging tolerance in *H. hamabo* will be resolved, which will also shed light on semi-mangrove plants.

## Materials and methods

### Sample preparation and sequencing

The *H. hamabo* tissue was originally obtained from the Institute of Botany, Jiangsu Province, and the Chinese Academy of Sciences. We obtained high-quality genomic DNA from *H. hamabo* leaves using a modified CTAB method [42]. Several tools and techniques were used to examine DNA quantity and quality: 0.8% agarose gel electrophoresis, a Qubit 3.0 Fluorometer (Life Technologies, Carlsbad, CA, USA) with the Qubit dsDNA HS Assay Kit, and a NanoDrop 2000 spectrophotometer (NanoDrop Technologies, Wilmington, DE, USA). We extracted total RNA using the TRIzol reagent (Invitrogen, CA, USA) and assessed its purity and integrity on a NanoDrop 2000 spectrophotometer and a Bioanalyzer 2100 system (Agilent Technologies, CA, USA). We assessed RNA density using 1.5% agarose gel electrophoresis. The Sequel Binding Kit 2.1, Sequel Sequence Kit 2.1, and Sequel SMRT Cell 1 M V2 were used for sequencing, and data were analyzed using SMRT Link 5.1 software. Initial filtering was performed based on the read quality values of the original data, and the effective data output after filtering was counted.

### Transcriptome sequencing and analysis

The transcriptome analysis was performed with seedlings grown from seeds of the *H. hamabo* individual that was used for genome sequencing. Four treatments were applied to seedlings with 8–10 true leaves: (*i*) Control (CK), the seedlings were irrigated with 1/4 Hoagland’s nutrient solution; (*ii*) Salt stress (S), the NaCl concentration of the irrigation solution was set to 3.5 wt% (simulated seawater); (*iii*) Waterlogging (W), a 1–2 cm layer of nutrient solution was maintained above the soil surface; and ($i\varpi$) A combination of S and W (SW). Leaves and roots were collected 5 min, 9 h, and 3 d after treatment, and RNA samples were isolated for Illumina sequencing using the paired-end 150-bp sequencing mode. All N bases, low-quality reads, and adapter sequences were removed to acquire the final clean reads. HISAT2 and Bowtie 2 (v2.2.2) were used to align clean reads to the *H. hamabo* reference genome with default parameters. FPKM (fragments per kb per million reads) was used for the normalization of gene and isoform expression values. All differentially expressed genes (DEGs) were identified using the R package edgeR with the criteria of false discovery rate < 0.05 and fold change ≥2. Several other R packages were also used to analyze gene expression patterns, including NbClust for DEG clustering and ggplot2 for visualization.

For WGCNA, we used the R package WGCNA (v1.66) to construct co-expression networks. Genes with FPKM values less than 1 were filtered out. The parameters of blockwiseModules () were set to soft power = 16, minModulesSize = 30, mergeCutHeight = 0.25, and default parameters were used for other settings. The function pickSoftThreshold () in WGCNA was used to choose the soft power. Candidate hub genes were identified based on a thresholding of 0.6. Cytoscape was used to visualize the co-expression network.

### Assembling third-generation long reads

After removal of adaptor sequences from sequences generated by the PacBio Sequel platform, we obtained 17 800 118 subreads. The longest 150x subreads were chosen for subsequent genome assembly. First, the *H. hamabo* genome was assembled using FALCON (v0.2.2) (https://github.com/PacificBiosciences/FALCON/tree/v0.2.2) with default parameters. The Arrow pipeline in the SMRT Link 4 toolkit was used to polish the primary assembly. Finally, short reads from Illumina sequencing were used to further correct any remaining errors using Pilon v1.22 [43]. To evaluate the integrity of the assembly and the uniformity of the sequencing coverage, CLR (Continuous Long Reads) subreads of *H. hamabo* were selected and matched back to the assembled genome using the comparison tool minimap2 [44] with default parameters. The comparison rate of reads, the extent of genome coverage, and the depth distribution were determined to evaluate the integrity of the assembly and the uniformity of the sequencing coverage.

### Genome quality evaluation

To assess assembly completeness, we first mapped the CLRs to the final assembly with the default parameters of minimap2 (v2.5) [44]. We then evaluated the assembly completeness using BUSCO v3.0.2 [45]. We also mapped the Illumina paired-end short reads to the genome to confirm the high quality of the contig assembly based on a high ratio of alignment and a high distribution peak of the insertion length distribution. BWA 0.7.17 software was used to align the Illumina short reads to the reference genome to evaluate its accuracy at the single-base level [46]. Homozygous SNP loci were identified with the GATK 4.0.8.1 [47] package. We also assessed genome quality by calculating the LAI using the LTR_Finder [48] and LTR_retriever [49] packages.

### Genome annotation

Repetitive sequences were identified in the *H. hamab*o genome using two combined methods: homologous annotation and *de novo* prediction. For homology-based analysis, the RepeatMasker (open-4.0.9) [50] package was used to identify known TEs from the Repbase TE library [51]. For *de novo* prediction, the comprehensive pipeline RepeatModeler (http://www.repeatmasker.org/RepeatModeler/) was used to construct a *H. hamabo* genome repeat library, and the subprograms RECON (v1.08) [52] and RepeatScout (v1.0.5) [53] were used to identify decentralized repeat sequences in the genome. LTR_Finder (v1.0.7) [48] was used to extract all LTR sequences. Tandem repeats were extracted with the TRF [54] package. Finally, RepeatMaker was used to merge the results from the two methods to identify the repetitive content.

A combination of homology-based, RNA-seq-guided and *ab initio* methods were used to predict protein-coding genes. Repetitive sequences were masked with RepeatMasker, and *ab initio* gene prediction was performed with the Augustus (v3.3.1) package [55–57] and GeneScan software [58]. The previous RNA-seq dataset was chosen as a source for model training. For homology-based prediction, we used Exonerate to align query sequences to the genome and predict coding genes. For RNA-seq-guided gene prediction, the TopHat (v2.1.1) package [59] was used to assemble all transcripts, and Cufflinks (v2.2.1) [60] was used for gene structure identification. We used Maker (v3.00) [61] to integrate the results of all gene-prediction methods.

The National Center for Biotechnology Information (NCBI) non-redundant (NR) database, the TrEMBL [62], InterPro [63], and Swiss-Prot [62] protein databases, and the KEGG database [64] were used to predict gene functions based on the best matched alignments using BLASTP [65, 66] with an E-value threshold of 1e^−5^. The InterPro protein database was used for protein domain annotation with PfamScan [67] and InterProScan v5.35–74.0 [68]. Motifs and domains within gene models were identified with PFAM databases [69], and Blast2GO [70] was used to map GO [71] terms to each gene.

### Analysis of gene families

The longest transcripts of each protein-coding gene in *H. hamabo* and other carefully selected species (*Arabidopsis thaliana* [72]*, Corchorus olitorius* [73]*, Gossypium raimondii* [74]*, H. syriacus* [34]*, Theobroma cacao* [75]*, Bombax ceiba* [76]*, Durio zibethinus* [77]*, H. cannabinus* [17]*, Populus trichocarpa* [78]*, Vitis vinifera* [79]*, Corchorus capsularis* [80]*, Glycine max* [81] and *Solanum lycopersicum* [82]) were used to cluster the gene families. All longest transcripts were extracted using a python script from the OrthoFinder repository at Github (https://github.com/davidemms/OrthoFinder). BLASTP [65] was used to search for all possible homologs within all species with an E-value threshold of 1e^−5^. OrthoFinder v14 [83] was used to classify genes into gene families with default parameters. To exclude false positive effects, genes with amino acid sequences shorter than 50 amino acids were filtered out. To assign functions to genes related to stress resistance, orthologous genes were identified from *H. hamabo* and *A. thaliana* using OrthoFinder, and functional annotations were obtained from the TAIR website. All stress resistance-related subfamilies were used to construct a phylogenetic tree, and all orthologs were identified.

### Phylogenetic analysis

To determine the evolutionary relationships among *H. hamabo* and other angiosperm species, we constructed a phylogenetic tree using protein-coding sequences of 586 low-copy orthologous genes. The translated amino acids of the genes were aligned using MUSCLE v3.8.31 [84] and then concatenated using a python script. We then used RAxML v8.2.11 [85] to reconstruct a consensus tree using the maximum likelihood (ML) algorithm with the rapid bootstrap method.

The MCMCTree program in the PAML (version 4.9 h) package was used for divergence time estimation, and the ultrametric phylogenetic tree with divergence times as branch lengths and the previously identified gene families were used for the analysis of gene family expansion and contraction using the CAFÉ package [86]. A random birth and death model was used to determine how gene families changed with time. All p-values of gene families were calculated, and a threshold of p < = 0.05 was used as the criterion for identification of rapidly evolving gene families.

### Analysis of WGD events

WGD software [87] was used for Ks-based paralog age distributions. All potential paralogs were detected with all-vs-all protein sequence blast using BLASTP with an E-value threshold of 10^−10^, and the MCL package was used for gene family construction. Mafft [88] was used to align each family. Gene families (with n members) for which n × (n − 1)/2 > `max pairwise` were removed, and a phylogenetic tree was built for each family using FastTree [89]. Ks values for each pair resulting from pairwise comparisons were obtained using a maximum likelihood algorithm in the CODEML program of the PAML v.4.4c package [90], and the weighted Ks values were used to construct the Ks distribution. Finally, we performed mixture modeling for all possible WGD inferences using the BGMM method. The ksrates package (https://github.com/VIB-PSB/ksrates) was used for rate adjustment, and the JCVI [91] and minimap packages [44] were used for syntenic visualization. The WGDI package [18] was used for collinearity anchor pair identification and analysis. First, all syntenic blocks were identified using the improved collinearity pipeline in WGDI with “p-value = 0.05”. Then the Ks value for each anchor gene pair located in syntenic blocks was calculated using the Ks pipeline in WGDI. A Ks dotplot of all anchor pairs was obtained using the blocks pipeline in WGDI, and the kspeaks pipeline in WGDI was used for distribution analysis of the Ks median value for each syntenic block. Finally, all of the above results from the Ks distributions were summarized in a single picture using the ggplot2 package.

## Acknowledgments

This work was supported by the Six Talent Peaks Project of Jiangsu Province (NY-042), the Open Fund of the Jiangsu Key Laboratory for the Research and Utilization of Plant Resources (JSPKLB201928), and the Talent Training Funds of the Institute of Botany, Jiangsu Province and Chinese Academy of Sciences.

## Author contributions

Conceived and designed the experiments: GC. Performed the experiments: WZ, ZF, and NL. Analyzed the data: WZ, XJ, HS, ZF, YR, CD, JJ, SA, YV, and GC. Contributed reagents/materials/analysis tools: HJ, LZ, YC, and YY. Wrote the paper: WZ, XJ, HS, and GC. All authors read and approved the final manuscript.

## Data availability

The data supporting the findings of this work are available within the paper and its Supporting Information files. The data sets generated and analyzed during this study are available from the corresponding author upon request. All the whole-genome raw data generated during this study have been deposited in the SRA database under BioProject number PRJNA759075. Transcriptome clean data have been deposited in the SRA database under BioProject number PRJNA759717. The final chromosome-scale genome assembly and annotation data have been deposited in the Figshare database (https://doi.org/10.6084/m9.figshare.19142558.v1).

## Conflict of interest statement

The authors declare no competing financial interests.

## Supplementary data

Supplementary data is available at *Horticulture Research * online.

## Supplementary Material

Web_Material_uhac067
