# Supplementary material for: The genome of Hibiscus hamabo reveals its adaptation to saline and waterlogged habitat
Source: Hortic Res. 2022 Mar 23;9:uhac067. doi: 10.1093/hr/uhac067 (PMC9039499; doi:10.1093/hr/uhac067)
Supplement: Web_Material_uhac067 [file web_material_uhac067.zip › supplementary_information.docx]

# Supplementary Information

**Article title**: The genome of Hibiscus hamabo reveals its adaptation to saline and waterlogged habitat

Authors: Zhiquan Wang, Jia-Yu Xue, Shuai-Ya Hu, Fengjiao Zhang, Ranran Yu, Dijun Chen, Yves Van de Peer, Jiafu Jiang, Aiping Song, Longjie Ni, Jianfeng Hua, Zhiguo Lu, Chaoguang Yu, Yunlong Yin, Chunsun Gu

**Supplementary Notes**

**Note S1** **Sequencing and assembly**

**Sample and sequencing**

The *H. hamabo* tissue was originally collected from Institute of Botany,Jiangsu Province and Chinese Academy of Sciences (NANJING BOTANICAL GARDEN MEM.SUN YAT-SEN). High-quality genomic DNA was extracted from leaves using a modified CTAB method. The quality and quantity of the extracted DNA were examined using a NanoDrop 2000 spectrophotometer (NanoDrop Technologies, Wilmington, DE, USA), Qubit dsDNA HS Assay Kit on a Qubit 3.0 Fluorometer (Life Technologies, Carlsbad, CA, USA) and electrophoresis on a 0.8% agarose gel, respectively. Total RNA was extracted using Trizol reagent (Invitrogen, CA, USA). RNA purity and integrity was monitored by NanoDrop 2000 spectrophotometer (NanoDrop Technologies, Wilmington, DE, USA) and a Bioanalyzer 2100 system (Agilent Technologies, CA, USA). RNA contamination was assessed by 1.5% agarose gel.

Sequel Binding Kit 2.1, Sequel Sequence Kit 2.1 and Sequel SMRT Cell 1M V2 are used for sequencing and data was sequenced using SMRT Link 5.1 software. According to the read quality value of the original data, the initial filtering is carried out, and the effective data output after filtering is counted, number of Polymerase Read, average sequence length, sequence N50 length, and maximum Subread average length are shown in **Table S2**. As can be seen from **Table S2**, *H. hamabo* samples produced 138.1GB of data. Data statistics of Subread are shown in **Table S3**. After filtering low-quality data, the Polymerase Read length distribution is shown in **Fig.** **S1**.

**Ploidy analysis and genome size prediction**

1. To accumulate metaphases, treat excised root tips (5-20 mm long) with 8-hydroxyquinoline for 1-2 h at room temperature, then 1-2 h at 4°C.

2. Quickly blot material and transfer to fixative (Alcohol:acetic acid fixative: three parts 96% ethanol (or 100% methanol) to one part glacial acetic acid).

3. Leave for 2 h at room temperature and then transfer to new fixative (or 70% or 96% ethanol) and store at -20°C.

4. Wash 2-10 root tips twice for 10 min in 2-5 ml enzyme buffer (40 ml 100 mM citric acid + 60 ml 100 mM tri-sodium-citrate, adjust to pH 4.8, store stock solution at 4°C. Dilute 1: 10 in water for use) to remove the fixative (until they sink).

5. Transfer material into 1-2 ml enzyme solution and digest at 37°C until the material is soft.

Enzyme solution: 2% (w/v) cellulase from *Aspergillus niger* (Calbiochem, 21947, 4000 units g^-1^; final concentration: 80 units ml^-1^) or a mixture of 1.8% Calbiochem and 0.2% 'Onozuka' RS cellulase (5000 units g^-1^; final concentration: 10 units ml^-1^) and 3% (v/v) pectinase from *A.* *niger* (solution in 40% glycerol, Sigma P4716, 450 units ml^-1^; final concentration: 13.5 units ml^-1^). Make up in 1 x enzyme buffer. Store in aliquots at -20°C.

6. Wash material in 1x enzyme buffer for at least 15 min.

7. Transfer enough material for one preparation (typically one root) into 45% acetic acid in an embryo dish or small Petri dish for 1-5 min.

8. Make chromosome preparations on an acid-cleaned slide. Under the stereo microscope, in one drop (10-30 µl) of 45% acetic acid (or 60% acetic acid to increase dispersion of cytoplasm), dissect the meristematic tissue by removing as much of the other tissue as possible.

9. Apply coverslip to the material without trapping air bubbles. Carefully disperse the material between glass slide and coverslip by tapping the coverslip gently with a needle or flat back of a pencil, and then squash the cells, usually using the thumb with a pressure that just turns the nail white.

10. Check the slide under a phase contrast microscope. If not sufficiently flat, squash again.

11. Drop 20 ul DAPI on the slide, cover the coverslip, perform the microscopic examination with OLYMPUS BX53F microscope (Olympus, Tokyo, Japan).

DAPI (4' ,6-diamidino-2-phenylindole, Sigma): prepare DAPI stock solution of 100 tlg ml-1 in water.

12. Place the spread slide on to dry ice for 5-10 min (preferred method) or immerse into liquid nitrogen until frozen (about 30 s), then flick off the coverslip with a razor blade (Note 8). Allow the slide to air-dry.

Samples were placed in 400 ul Nuclei Extraction buffer, chopped with sharp blade for 30 seconds then filtered through a 50um filter, added 1600 ul of staining solution and put in dark for 30 minutes. Nuclei suspension was analyzed with CyFlow Space flow cytometer (Sysmex Partec, Muenster, Germany) and the corresponding FloMax software. The result is as shown in **Fig S2a：**

**Genome features estimation from Kmer method**

The short-reads from illumina platform were quality filtered by HTQC v1.92.310^1^ using the following method. Firstly, the adaptors were removed from the sequencing reads. Second, read pairs were excluded if any one end has an average quality lower than 20. Third, ends of reads were trimmed if the average quality lower than 20 in the sliding window size of 5 bp. Finally, read pairs with any end was shorter than 75 bp were removed. The quality filtered reads were used for genome size estimation. We generated the 17-mer occurrence distribution of sequencing reads from short libraries using the k-mer method and the proportion of repeat sequences and heterozygosity rate of the genome were determined using GCE ^2^.

**Genome assembly by third-generation long reads**

With one SMRT cells in PacBio Sequel platform, we generated 17,800,118 subreads by removing adaptor sequences within sequences. The longest 150X subreads data is used for genome assembly. The draft assembly of the genome is assembled using Falcon (v0.2.0) (https://github.com/PacificBiosciences/falcon) with default parameters. To correct errors in the primary assembly, we used the arrow pipeline from the SMRT link 4 toolkit to polish the genome (https://www.pacb.com/products-and-services/analytical-software/smrt-analysis/) after the initial assembly of the genome was completed. Finally, we used Illumina-derived short reads to correct any remaining errors by pilon^3^ (v1.22). Finally, the *H. hamabo* genome assembly had a total length of about 1,717 Mb, which accounted for ~90.4% of the genome size estimated by k-mer analysis, containing 4,358 contigs. The genomic indicators obtained after assembly are shown in (**Table S4**). As can be seen from Table S4, the contig N50 of *H. hamabo* genome reached 1.65 Mb. We conducted statistics on the proportion and GC content of A, G, C, T and N bases in the genome (**Table S5**).

In order to evaluate the integrity of assembly and the uniformity of sequencing coverage, CLR (Continuous Long Reads) subreads of *H. hamabo* were selected and matched back to the assembled genome using the comparison tool minimap2 ^4^ (the default parameter). The comparison rate of reads, the extent of genome coverage and the distribution of depth were counted to evaluate the integrity of assembly and the uniformity of sequencing coverage. The results are shown in (**Table S6**). CLR subreads were compared to the assembly results to obtain the coverage depth of each locus on the genome; Then, a 10K window was used to continuously slide the genome without overlap (the sequence length was less than 10K according to its true length) to calculate the average sequencing depth (the sum of the sequencing depth of all sites in the window/the window size) and the percentage of GC content within the window. Finally, a density map of contig GC content distribution and sequencing depth distribution was drawn based on the statistical data (**Fig S3**).

Based on the single-copy homologous gene set in OrthodB, BUSCO ^5^ was used to predict these genes and calculate their integrity, fragmentation, and possible loss rates. Thus, the integrity of the gene region in the whole assembly result was assessed. The Busco gene set used in this evaluation was embryophyta_odb9. The Busco evaluation results are shown in **Table S7.**

Reads were mapped to the reference genome with BWA, SNP calling was performed with GATK and filtered, and the number of homozygous and heterozygous SNPs was counted (the results are shown in **Table S8, Table S9**). INSERSIZE was counted according to the comparison results and was plotted (**Fig S4**).

**Hi-C technology help anchor contigs**

For anchored contigs, the clean reads pairs were generated from the Hi-C library and mapped to the polished *H. hamabo* genome using BWA (bwa-0.7.17) with the default parameters. Paired reads with mate mapped to a different contig were used to do the Hi-C associated scaffolding. Self-ligation, non-ligation and other invalid reads, such as Start NearRsite, PCR amplification, random break, Large Small-Fragments and Extreme Fragments, were filtered. Then contigs were clustered into groups with the agglomerative hierarchical clustering method in Lachesis ^6^. Lachesis was further applied to order and orient the clustered contigs.

As a result, 95× Hi-C paired-end reads using the allHiC pipeline scaffolded contigs to achieve a final assembly of 1,718 Mb (scaffold N50 = 36.3 Mb) in 1,915 scaffolds, accounting for 90.4% of the estimated genome. Forty-six longest pseudomolecules accounting for 88.9% (1690 Mbp) of the estimated genome, were selected to presumably correspond to the 46 chromosomes of the haplotype genome of *H. hamabo* (**Fig S5**). The genome-wide interaction heatmap shows a high-quality result of grouping and ordering by the Hi-C data (**Fig S6**).

**Genome quality evaluation**

To examine the assembly integrity, the CLR (Continuous Long Reads) subreads were aligned again onto the final assembly using minimap2 (v2.5) with default parameters. A total of 99.52% of raw reads can be mapped. The assembled genome was also subjected to BUSCO v3.0.2 with the OrthoDB to evaluate the completeness of the genome. Overall, 96.72% complete and 1.00% partial of the BUSCOs were identified in the assembled genome. Aligning short reads from Illumina platform to the genome, thus the high alignment ratio and single peak insertion length distribution demonstrated the high-quality of contig assembly. To evaluate the accuracy of the genome at the single base level, using the Illumina short read alignment to the reference genome of the *H. hamabo* by BWA 0.7.17 software^7^, we identified 23,288 homozygous SNP loci (0.0032% of total *H. hamabo* assembly) by GATK 4.0.8.1 ^8^ package.

**Note S2** **Genome annotation**

**Annotation of repetitive sequences**

The two methods are combined to identify the repeat contents in our genome, homology-based and de novo prediction. Homology-based analysis: We identified the known TEs within the *H. hamabo* genome using RepeatMasker (open-4.0.9)^9^ with the Repbase TE library^10^. RepeatProteinMask searches were also conducted using the TE protein database as a query library. De novo prediction: We constructed a denovo repeat library of the *H. hamabo* genome using RepeatModeler (http://www.repeatmasker.org/RepeatModeler/), which can automatically execute two core de novo repeat-finding programs, namely, RECON (v1.08)^11^ and RepeatScout (v1.0.5)^12^, to comprehensively conduct, refine and classify consensus models of putative interspersed repeats for the *H. hamabo* genome. Furthermore, we performed a de novo search for long terminal repeat (LTR) retrotransposons against the *H. hamabo* genome sequences using LTR_FINDER (v1.0.7) ^13^. We also identified tandem repeats using the Tandem Repeat Finder (TRF) package ^14^ and the non-interspersed repeat sequences, including low-complexity repeats, satellites and simple repeats, using RepeatMasker. Finally, we merge the lib library files of the two methods and use repeatmaker to identify the repeat contents. The workflow can be found in **Fig S7.**

As an important part of the genome, tandem repeats are classified into two major categories: tandem repeats and interspersed repeats. Tandem repeat sequences include microsatellite sequences, microsatellite sequences and so on. Scattered repeats, also known as transposon elements, include DNA transposons and retrotransposons that transpose in a DNA-DNA manner. The annotation results are shown in the following (**Table S9**, **Table S10).** With RepBase as the library, the TE bifurcation degree distribution diagram obtained by REPEATMASKER annotation is shown in **Fig S8**. The horizontal axis is the divergence degree of annotated TE sequences in the genome and corresponding sequences in the RepBase; the vertical axis is the percentage of TE sequences under the divergence degree in the genome, and different TE sequences are marked with different colors. The TE bifurcation distribution predicted by the De Novo method is shown in **Fig S9**. The abscissus is the degree of divergence between the annotated TE sequence and the corresponding repeat sequence in the De Novo library; The ordinate is the percentage of TE sequences in the whole genome under the bifurcation degree; Different TE's are indicated in different colors.

**Annotation of Protein coding gene**

We predicted protein-coding genes of the *H. hamabo* genome using three methods, including ab initio gene prediction, homology-based gene prediction and RNA-Seq-aided gene prediction. Prior to gene prediction, the assembled *H. hamabo* genome was hard and soft masked using RepeatMasker. We adopted Augustus (v3.3.1) ^15–17^ and Genescan ^18^ to perform ab initio gene prediction. Models used for each gene predictor were trained from a set of high-quality proteins generated from the RNA-Seq dataset. We used Exonerate (v2.2.0) to conduct homology-based gene prediction. First, the protein sequences were aligned to our genome assembly and predicted coding gene using Exonerate with the default parameters. To carry out RNA-Seq-aided gene prediction, we first assembled clean RNA-Seq reads into transcripts using tophat (v2.1.1) ^19^, and the gene structure were formed using Cufflinks (v2.2.1) ^20^. Finally, Maker (v3.00) ^21^ was used to integrate the prediction results of the three methods to predict genes models. The output included a set of consistent and non-overlapping sequence assemblies, which were used to describe the gene structures. The detailed annotation results are shown below (**Table S11, Table S12, Fig. S10)**.

**Functional annotation of protein-coding genes**

Gene functions were inferred according to the best match of the alignments to the National Center for Biotechnology Information (NCBI) Non-Redundant (NR), TrEMBL ^22^, InterPro^23^ and Swiss-Prot protein databases using BLASTP (ncbi blast v2.6.0+) ^24,25^ and the Kyoto Encyclopedia of Genes and Genomes (KEGG) database ^26^ with an E-value threshold of 1E-5. The protein domains were annotated using PfamScan (pfamscan _ version) ^27^ and InterProScan (v5.35-74.0) ^28^ based on InterPro protein databases. The motifs and domains within gene models were identified by PFAM databases ^29^. Gene Ontology ^30^ (GO) IDs for each gene were obtained from Blast2GO ^31^. As result showing in **Table S13.**

**Annotation of non-coding RNA genes**

We used tRNAscan-SE (v1.3.1) algorithms with default parameters to identify the genes associated with tRNA, which is an adaptor molecule composed of RNA used in biology to bridge the three-letter genetic code in messenger RNA (mRNA) with the twenty-letter code of amino acids in proteins. For rRNA identification, we first downloaded the closely related species rRNA sequences from the Ensembl database. Then rRNAs in the database were aligned against our genome using blastn ^24,25^ with cutoff of E-value<1e-5, identity ≥85% and match length ≥50 bp. snoRNAs are a class of small RNA molecules that guide chemical modifications of other RNAs, mainly ribosomal RNAs, transfer RNAs and small nuclear RNAs.

MiRNAs and snRNAs were identified by Infernal (v1.1.2) ^32^ software against the Rfam (v14.1) database ^29^ with default parameters.

**Note S3** **Evolutionary analysis**

**Gene family clustering**

To cluster families from protein-coding genes, proteins from the longest transcripts of each gene from *H. hamabo* and other angiosperm species, including *Arabidopsis thaliana, Corchorus olitorius, Gossypium raimondii, Hibiscus syriacus, Theobroma cacao, Bombax ceiba, Durio zibethinus, Hibiscus cannabinus, Populus trichocarpa, Vitis vinifera, Corchorus capsularis, Glycine max, H. hamabo, Solanum lycopersicum*. All protein were extracted and aligned to each other using BLASTP ^25^ programs (NCBI blast v2.6.0) with a maximal e-value of 1e-5. To exclude putative fragmented genes, identity less than 30%, coverage less than 50% and genes encoding protein sequences shorter than 50 bp amino acids were filtered out. Orthofinder (v14-137) ^33^ was used to cluster genes from these different species into gene families by default parameters. The unique families of *H. hamabo* were used for GO enrichment analysis (**Fig S11)** using R package clusterprofiler.

**Phylogenetic and divergence time analysis**

To reveal phylogenetic relationships among *H. hamabo* and other closely related species, protein sequences from 586 low-copy orthologous genes were identified by Orthofinder (v14-137) and further used for phylogenetic analyses. The protein sequences of the low-copy orthologous genes were aligned with MUSCLE (v3.8.31) ^34^ program, and the corresponding Coding DNA Sequences (CDS) alignments were generated and concatenated with the guidance of protein alignment. RAxML (v8.2.11) ^35^ were used to construct the phylogenetic tree with the maximum Likelihood method. The phylogenetic relationship of other closely related species was consistent with previous studies. MCMCTree program in the PAML ((version 4.9 h) ^36^ package was used for divergence time estimation (**Fig S12)**.

**WGD analysis**

JCVI package was used for syntenic blocks detection and visualization, the intra-genomic comparison within *H. hamabo* shows a pattern of 6:6 (**Fig. S14)**, which suggests an unusual history of polyploidization for this species. We first calculated the synonymous substitutions per synonymous sites (Ks) for all the paralogues (paranome) of *H. hamabo* using wgd package (see method for details), and only one single signature peak between 0.15-0.2 was observed (**Fig. S13a**). After rate-adjusting, two peaks at Ks=0.21 (mode a) and Ks=0.56 (mode b) emerged respectively, suggesting two polyploidy events in *H. hamabo*. As *H. hamabo*’s speciation peaks with *H. cannabinus* and *H.* *syriacus* were located at 0.12 and 0.18 (**Fig. S13b**), it is clear that these two polyploidy events took place in the common ancestor of all *Hibiscus* plants. To precisely locate the position of the WGD of Ks=0.56, the paralogs of *G. raimondii* and the reciprocal best hit (RBH) orthologs were extracted, and the Ks distribution show a WGD peak of *G. raimondii* at Ks≈0.5 (**Fig. S15c**) which is consistant with that of *H.* *hamabo* (mode b, Ks≈ 0.56) and a speciation peak at Ks ≈0.3 between *H.* *hamabo* and *G. raimondii* suggesting that there was a shared WGD event occurred before the speciation between *H.* *hamabo* and *G. raimondii.*

According to the results above, *H.* *hamabo* and *H. cannabinus* should have experienced the same polyploidy events; however, we performed subsequent inter-genomic synteny analyses between the two species using several different methods, and interestingly all results showed a 6:2 or 3:1 pattern in between (**Fig. 2c,2d,Fig. S14**), indicating that *H. hamabo* should have undergone an extra independent whole genome triplication (WGT) event after its spilt from the common ancestor of *H. hamabo* and *H. cannabinus*. Then back to the Ks peak 0.21, if there was a species-specific WGT in *H. hamabo*, the WGT peak is probably covered by or integrated into this 0.21 peak.

To more precisely distinguish the Ks values’ peak of triplicated genes from peaks of other WGD events, WGDI package was used for collinearity anchor-pairs identifying and analysing (see method), the anchor-pair genes retained in all syntenic blocks were extracted, then another parameter-the Ks median value of every syntenic block was used for subsequent analyses. As a result, two very close but independent peaks located at Ks value 0.1-0.2 and 0.2-0.3 were identified respectively, representing two recent polyploidy events (**Fig. 2e, Fig. S16**): the dot plots of synteny blocks representing the most recent WGT event show that a pattern of each block having three syntenic counterparts.

**Note S4** **Analysis of adaptive mechanism**

**Sample treatment**

The transcriptome analysis was conducted on seedlings grown from seeds of the H. hamabo tree that was used in the genome sequencing. Four treatments were established on seedlings with 8–10 true leaves: (i) Control (CK), the seedlings were irrigated with 1/4 Hoagland’ nutrient solution; (ii) Salt stress (S), the salt (NaCl) concentration of the irrigation solution was set at 3.5 wt% (simulated seawater); (iii) Waterlogging (W), a 1–2 cm layer of nutrient solution above the soil surface was maintained; and (iv) A combination of S and W (SW). Leaves and roots were collected 5 min, 9 h and 3 d after treatment (**Table S15**).

**Transcriptome sequencing and analyses of *H. hamabo***

Samples were collected and flash-frozen in liquid nitrogen and, then treated by trizol in - 80℃ until RNA extraction. Extracting total RNA from tissues by trizol. The RNA degradation and contamination was monitored on 1.5% agarose gels. Then RNA purity was checked using the NanoPhotometer® spectrophotometer (IMPLEN, CA, USA). The next RNA concentration was measured using Qubit® RNA Assay Kit in Qubit® 3.0 Flurometer (Life Technologies, CA, USA). RNA integrity was assessed using the RNA Nano 6000 Assay Kit of the Agilent Bioanalyzer 2100 system (Agilent Technologies, CA, USA). The clustering of the index-coded samples was performed on a cBot Cluster Generation System using HiSeq X Ten Cluster Kit (Illumia) according to the manufacturer’s instructions. After cluster generation, the library preparations were sequenced on an Illumina Hiseq X Ten platform and 150bp paired-end reads were generated.

Before performing any further analyses, quality control is required in order to detect whether the data is qualified or not. Software FraserQC (v1.2) was used to do quality control. Sequencing reads were aligned to the *H. hamabo* genome sequence using Tophat2 (v2.1.1) and bowtie2 (v2.2.2) in default parameter. Genes and isoforms expression level are quantified by a software package: RSEM (RNASeq by Expectation Maximization v1.3.0). RSEM computes maximum likelihood abundance estimates using the Expectation-Maximization (EM) algorithm as its statistical model. EdgeR (v3.6.8) package method was used for screening differentially expressed genes. EdgeR implements a range of statistical methodology based on the negative binomial distribution, including empirical Bayes estimation, exact tests, generalized linear models and quasi-likelihood tests. We screened differentially expressed genes according to the following criteria: Foldchange ≥2 and FDR <0.05.

**Ion concentration measurements**

The seedlings with the same growth (3~6 functional leaves) of the *H. hamabo* tree grown from seeds were transplanted into small POTS with substrate (river sand: vermiculite = 1:1) and irrigated with 1/4 Hoagland nutrient solution for 7 days, the treaement methods were depicted as follows: (ⅰ) **CK：**Control (no NaCl; well drained). (ⅱ) **S：**Salinity (well drained pots watered with **0.35%** NaCl solution). (ⅲ) **W：**Waterlogging (submerged pots (**1~2 cm** above the soil surface); no NaCl). (ⅳ) **SW：**Combined waterlogging and salinity (pots submerged in NaCl solution). Root and leaf were sampled at 5 min,5 h,9 h,24 h,3d and 6 d after treatment, with three biology repeat for each treatment and each biological replicate consisted of 3 plants.

The ion concentration of Na^+^ and K^+^ were measured, the Na^+^*/*K^+^ ratio was used for significant difference analysis. As a result, Na^+^/K^+^ in 9 h **S** group, 24 h **SW** group and 3 d **SW** group was significantly higher than that in the control group, and Na^+^/K^+^ in the root system was significantly higher than that in the leaves, suggesting that *H. hamabo* may response to salt stress at 9 h, 24 h and 3 d, and may be a kind of K- preference and Na- refusing halophyte.

**Reference**

1 Yang X, Liu D, Liu F *et al.* HTQC: a fast quality control toolkit for Illumina sequencing data. *BMC Bioinformatics* 2013; **14**: 33.

2 Liu B, Shi Y, Yuan J *et al.* Estimation of genomic characteristics by analyzing k-mer frequency in de novo genome projects. *arXiv:13082012 [q-bio]* 2020.http://arxiv.org/abs/1308.2012 (accessed 19 Sep2021).

3 Walker BJ, Abeel T, Shea T *et al.* Pilon: An Integrated Tool for Comprehensive Microbial Variant Detection and Genome Assembly Improvement. *PLoS ONE* 2014; **9**: e112963.

4 Li H. Minimap2: pairwise alignment for nucleotide sequences. *Bioinformatics* 2018; **34**: 3094–3100.

5 Simão FA, Waterhouse RM, Ioannidis P, Kriventseva EV, Zdobnov EM. BUSCO: assessing genome assembly and annotation completeness with single-copy orthologs. *Bioinformatics* 2015; **31**: 3210–3212.

6 Burton JN, Adey A, Patwardhan RP, Qiu R, Kitzman JO, Shendure J. Chromosome-scale scaffolding of de novo genome assemblies based on chromatin interactions. *Nat Biotechnol* 2013; **31**: 1119–1125.

7 Li H. Aligning sequence reads, clone sequences and assembly contigs with BWA-MEM. *arXiv:13033997 [q-bio]* 2013.http://arxiv.org/abs/1303.3997 (accessed 10 Aug2021).

8 McKenna A, Hanna M, Banks E *et al.* The Genome Analysis Toolkit: A MapReduce framework for analyzing next-generation DNA sequencing data. *Genome Research* 2010; **20**: 1297–1303.

9 Tarailo‐Graovac M, Chen N. Using RepeatMasker to Identify Repetitive Elements in Genomic Sequences. *Current Protocols in Bioinformatics* 2009; **25**. doi:10.1002/0471250953.bi0410s25.

10 Jurka J. Repbase Update: a database and an electronic journal of repetitive elements. *Trends in Genetics* 2000; **16**: 418–420.

11 Bao Z. Automated De Novo Identification of Repeat Sequence Families in Sequenced Genomes. *Genome Research* 2002; **12**: 1269–1276.

12 Price AL, Jones NC, Pevzner PA. De novo identification of repeat families in large genomes. *Bioinformatics* 2005; **21**: i351–i358.

13 Xu Z, Wang H. LTR_FINDER: an efficient tool for the prediction of full-length LTR retrotransposons. *Nucleic Acids Research* 2007; **35**: W265–W268.

14 Benson G. Tandem repeats finder: a program to analyze DNA sequences. *Nucleic Acids Research* 1999; **27**: 573–580.

15 Stanke M, Steinkamp R, Waack S, Morgenstern B. AUGUSTUS: a web server for gene finding in eukaryotes. *Nucleic Acids Research* 2004; **32**: W309–W312.

16 Stanke M, Keller O, Gunduz I, Hayes A, Waack S, Morgenstern B. AUGUSTUS: ab initio prediction of alternative transcripts. *Nucleic Acids Research* 2006; **34**: W435–W439.

17 Stanke M, Morgenstern B. AUGUSTUS: a web server for gene prediction in eukaryotes that allows user-defined constraints. *Nucleic Acids Research* 2005; **33**: W465–W467.

18 Burge C, Karlin S. Prediction of complete gene structures in human genomic DNA. *Journal of Molecular Biology* 1997; **268**: 78–94.

19 Trapnell C, Pachter L, Salzberg SL. TopHat: discovering splice junctions with RNA-Seq. *Bioinformatics* 2009; **25**: 1105–1111.

20 Trapnell C, Williams BA, Pertea G *et al.* Transcript assembly and quantification by RNA-Seq reveals unannotated transcripts and isoform switching during cell differentiation. *Nat Biotechnol* 2010; **28**: 511–515.

21 Cantarel BL, Korf I, Robb SMC *et al.* MAKER: An easy-to-use annotation pipeline designed for emerging model organism genomes. *Genome Research* 2007; **18**: 188–196.

22 Boeckmann B. The SWISS-PROT protein knowledgebase and its supplement TrEMBL in 2003. *Nucleic Acids Research* 2003; **31**: 365–370.

23 Mitchell A, Chang H-Y, Daugherty L *et al.* The InterPro protein families database: the classification resource after 15 years. *Nucleic Acids Research* 2015; **43**: D213–D221.

24 Altschul S. Gapped BLAST and PSI-BLAST: a new generation of protein database search programs. *Nucleic Acids Research* 1997; **25**: 3389–3402.

25 Camacho C, Coulouris G, Avagyan V *et al.* BLAST+: architecture and applications. *BMC Bioinformatics* 2009; **10**: 421.

26 Kanehisa M, Goto S, Sato Y, Furumichi M, Tanabe M. KEGG for integration and interpretation of large-scale molecular data sets. *Nucleic Acids Research* 2012; **40**: D109–D114.

27 Mistry J, Bateman A, Finn RD. Predicting active site residue annotations in the Pfam database. *BMC Bioinformatics* 2007; **8**: 298.

28 Jones P, Binns D, Chang H-Y *et al.* InterProScan 5: genome-scale protein function classification. *Bioinformatics* 2014; **30**: 1236–1240.

29 Punta M, Coggill PC, Eberhardt RY *et al.* The Pfam protein families database. *Nucleic Acids Research* 2012; **40**: D290–D301.

30 Ashburner M, Ball CA, Blake JA *et al.* Gene Ontology: tool for the unification of biology. *Nat Genet* 2000; **25**: 25–29.

31 Conesa A, Götz S. Blast2GO: A Comprehensive Suite for Functional Analysis in Plant Genomics. *International Journal of Plant Genomics* 2008; **2008**: 1–12.

32 Nawrocki EP, Kolbe DL, Eddy SR. Infernal 1.0: inference of RNA alignments. *Bioinformatics* 2009; **25**: 1335–1337.

33 Emms DM, Kelly S. OrthoFinder: phylogenetic orthology inference for comparative genomics. *Genome Biol* 2019; **20**: 238.

34 Edgar RC. MUSCLE: multiple sequence alignment with high accuracy and high throughput. *Nucleic Acids Research* 2004; **32**: 1792–1797.

35 Stamatakis A. RAxML version 8: a tool for phylogenetic analysis and post-analysis of large phylogenies. *Bioinformatics* 2014; **30**: 1312–1313.

36 Yang Z. PAML: a program package for phylogenetic analysis by maximum likelihood. *Bioinformatics* 1997; **13**: 555–556.

## Supplementary Information Figures


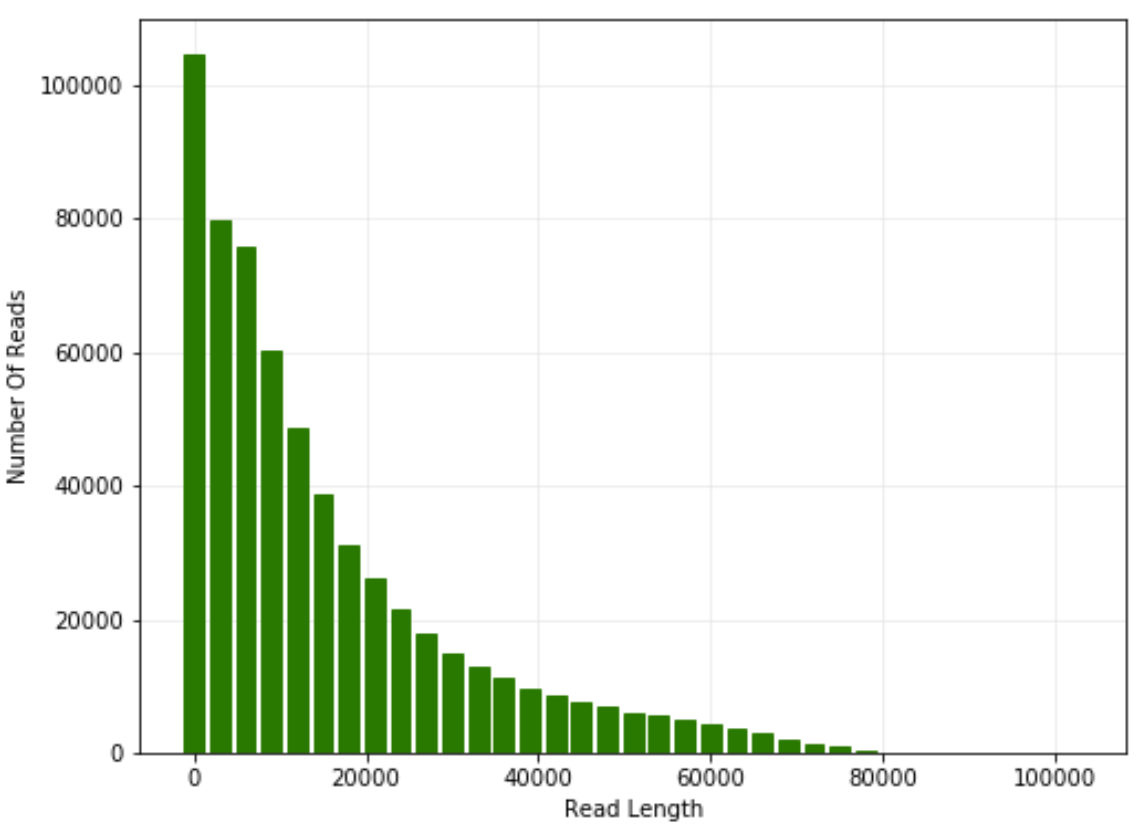

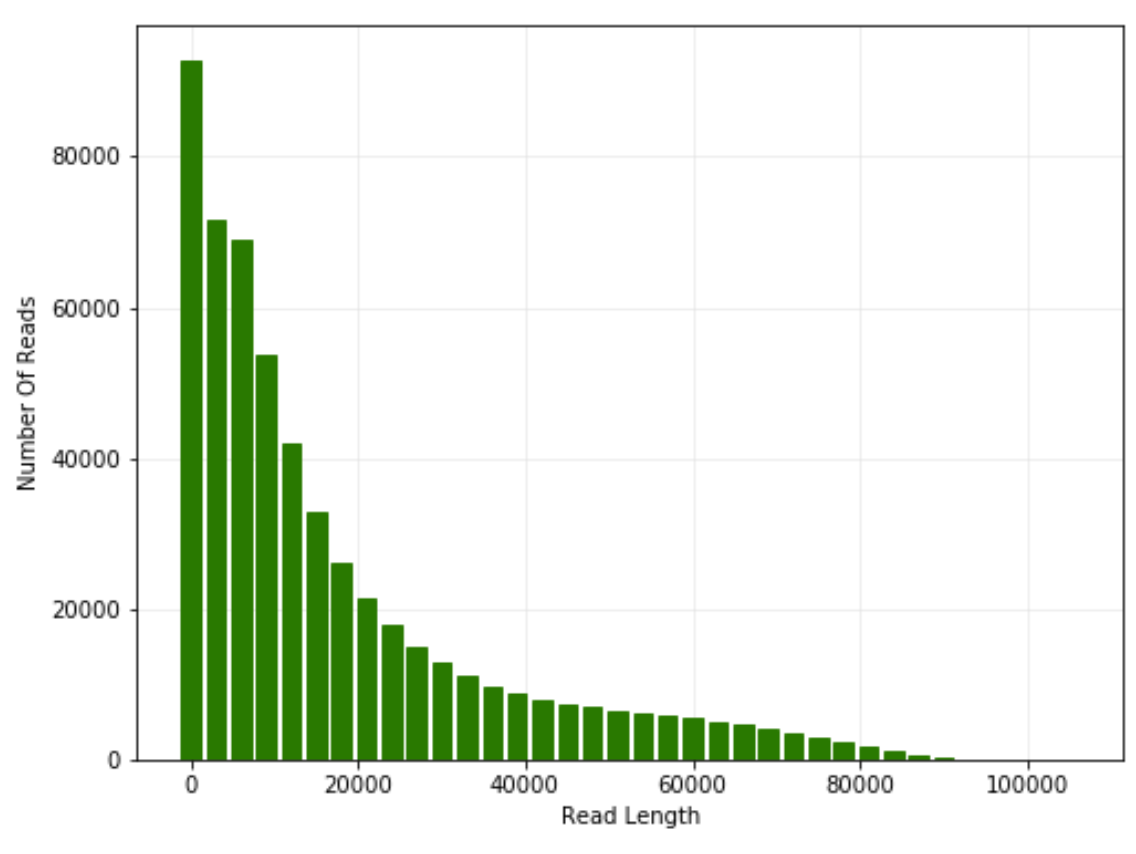

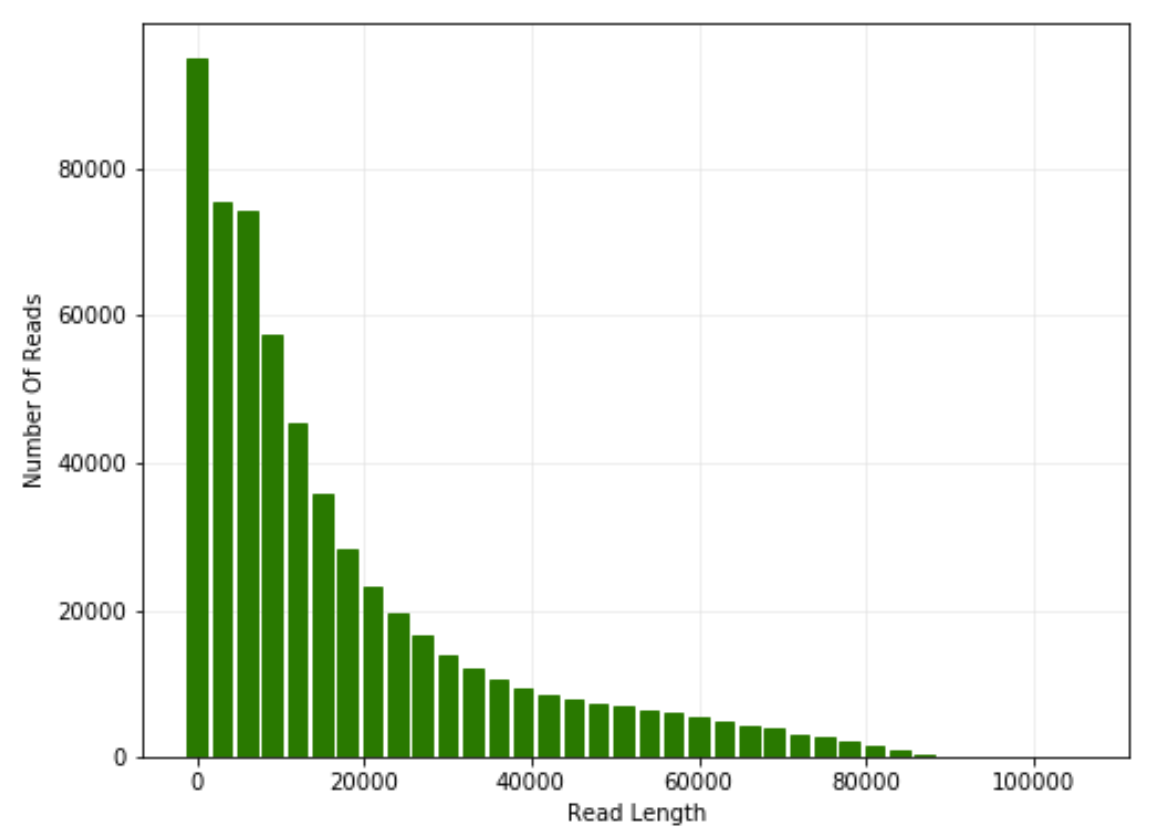

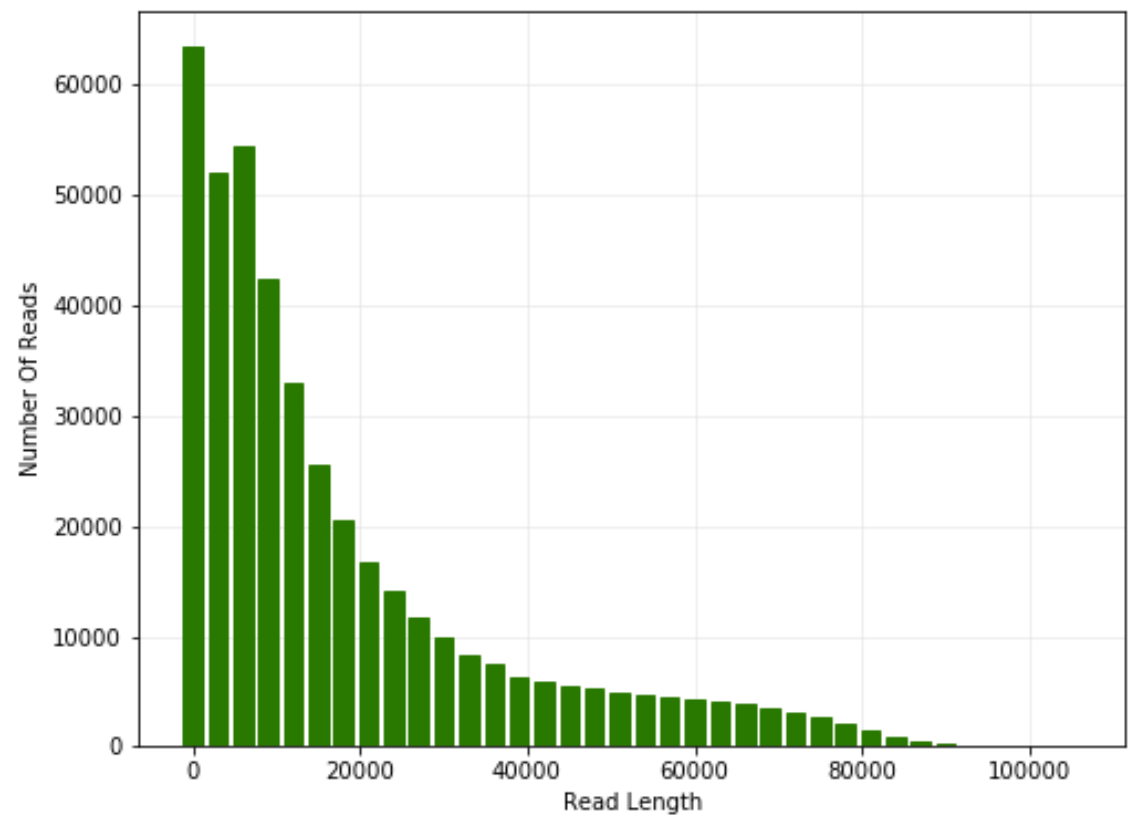


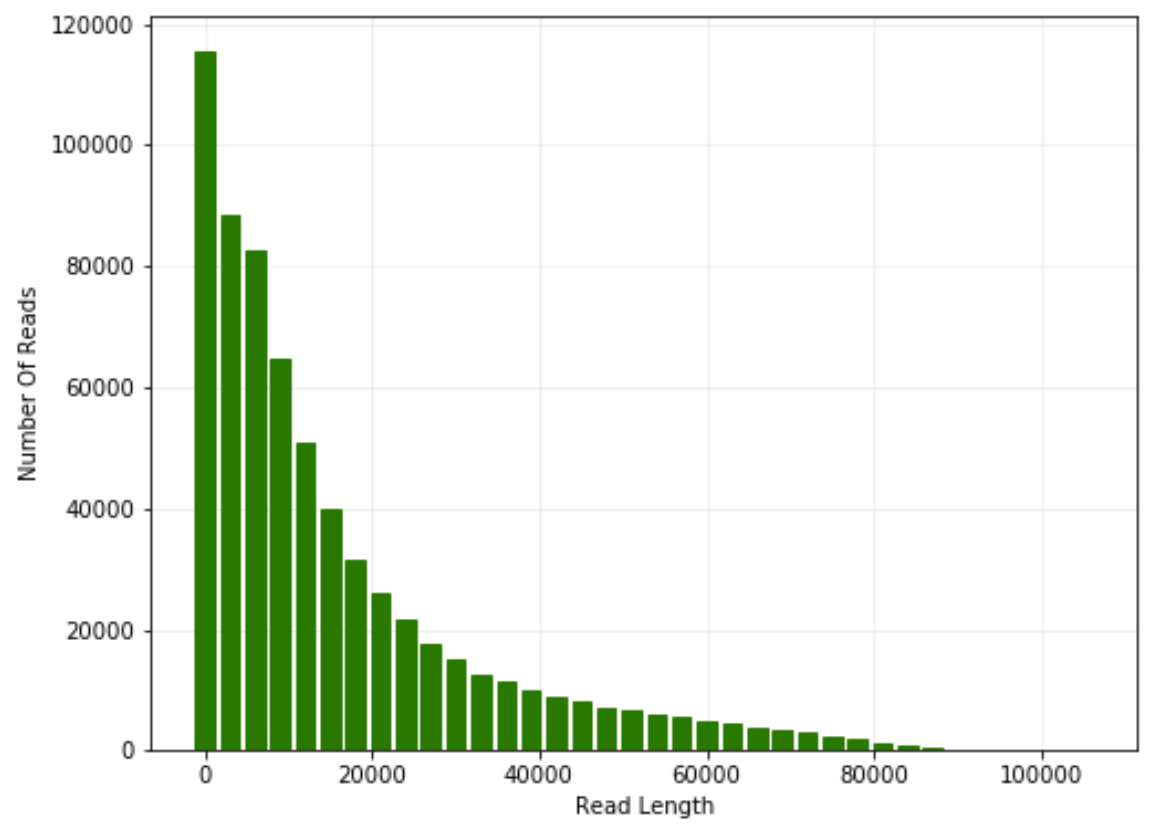

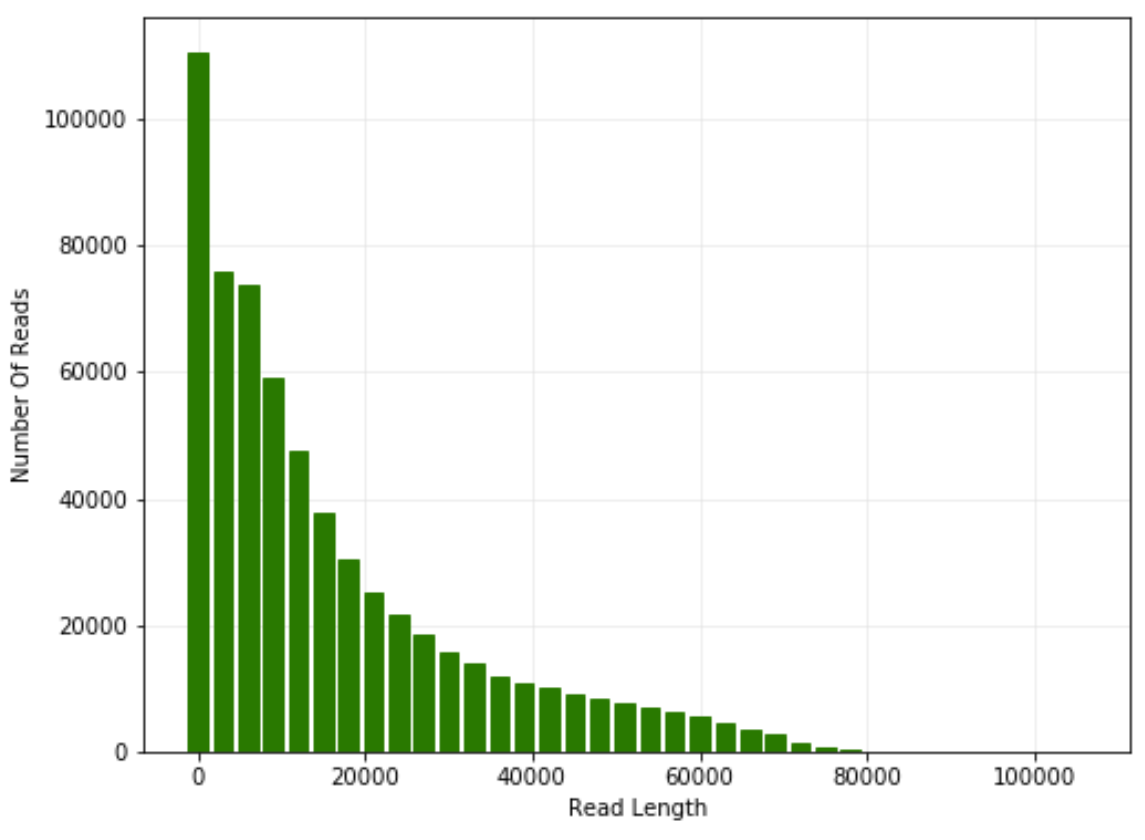


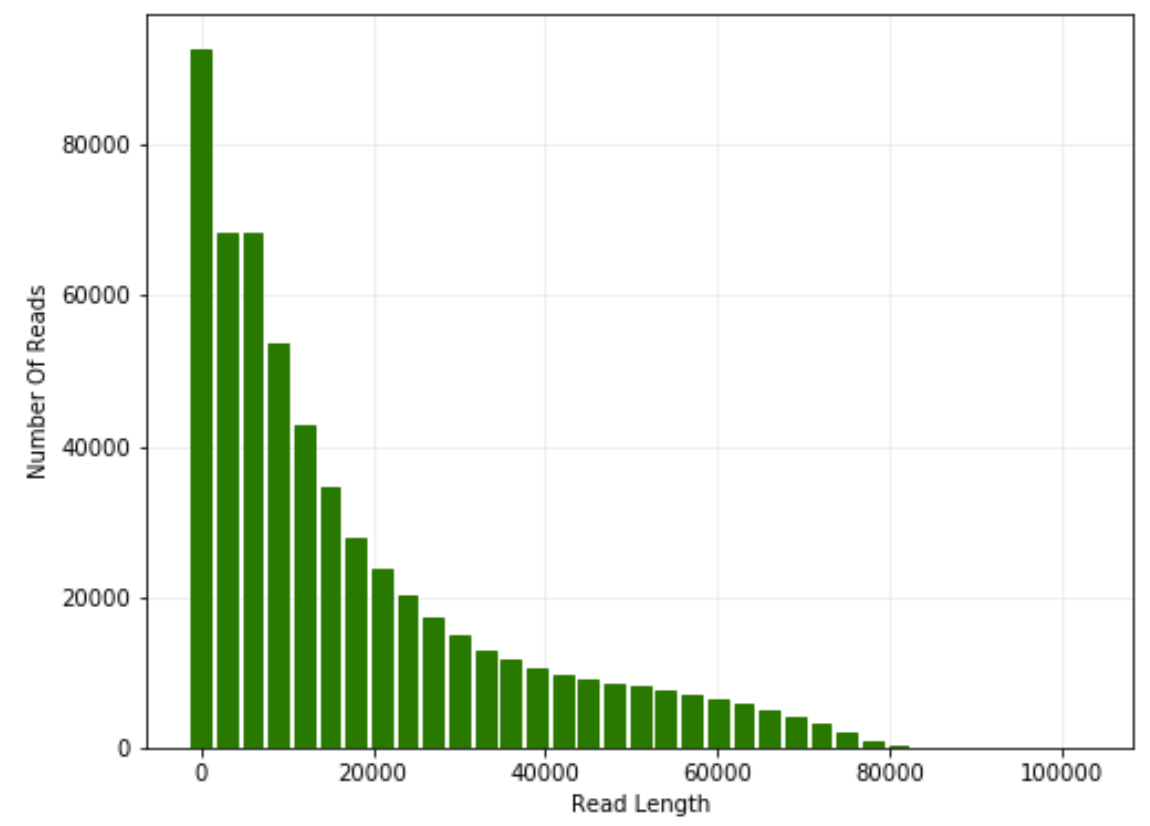

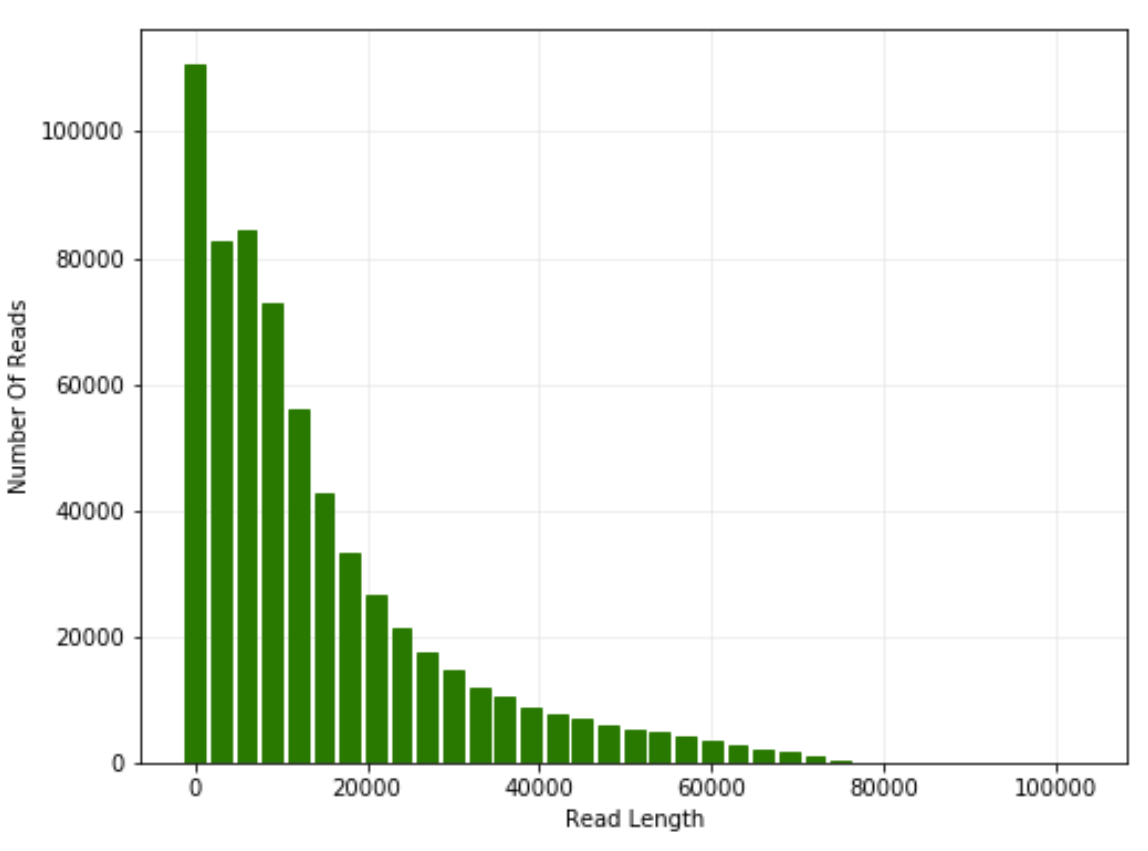


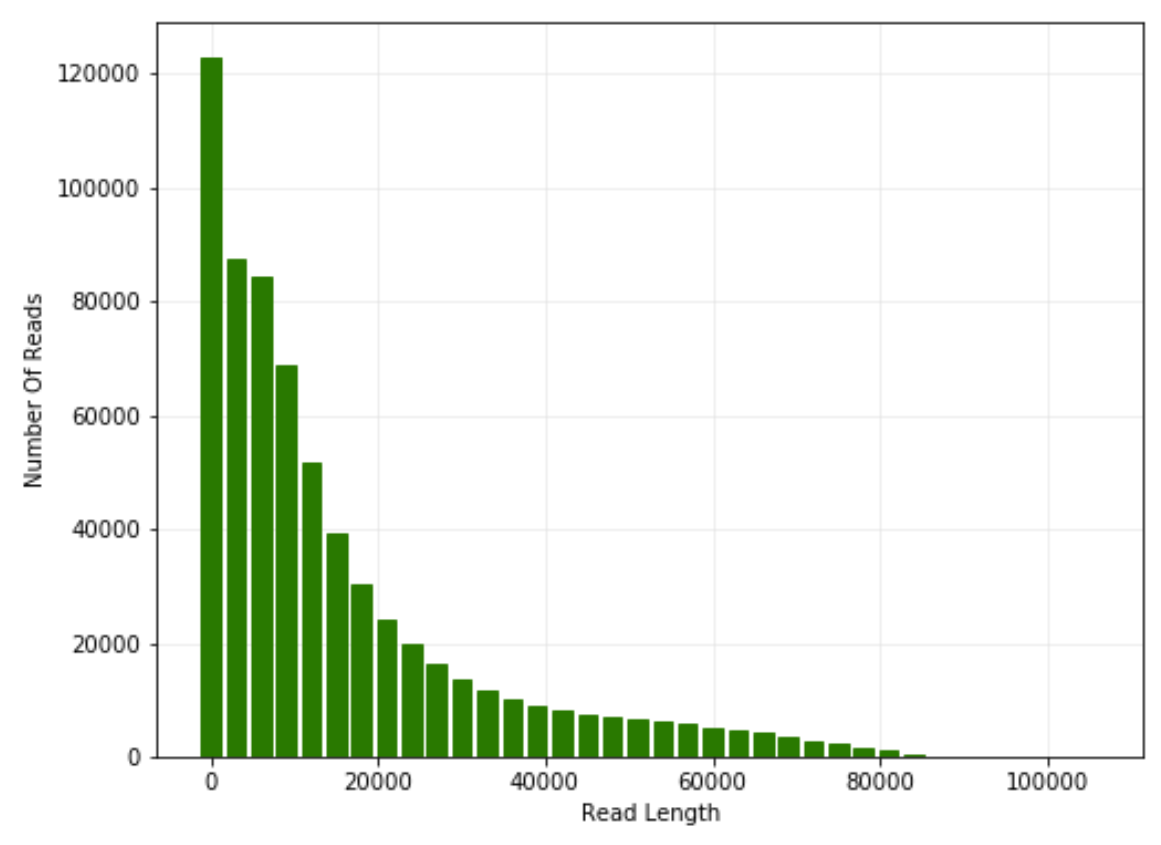

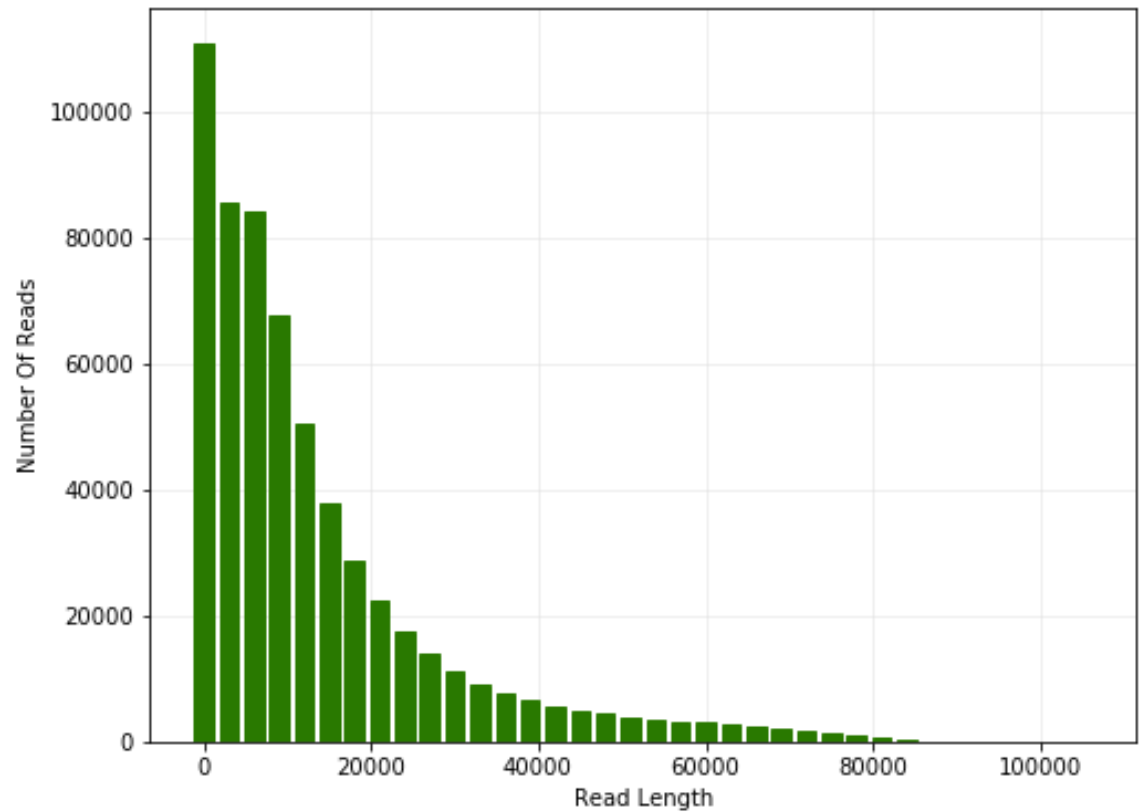


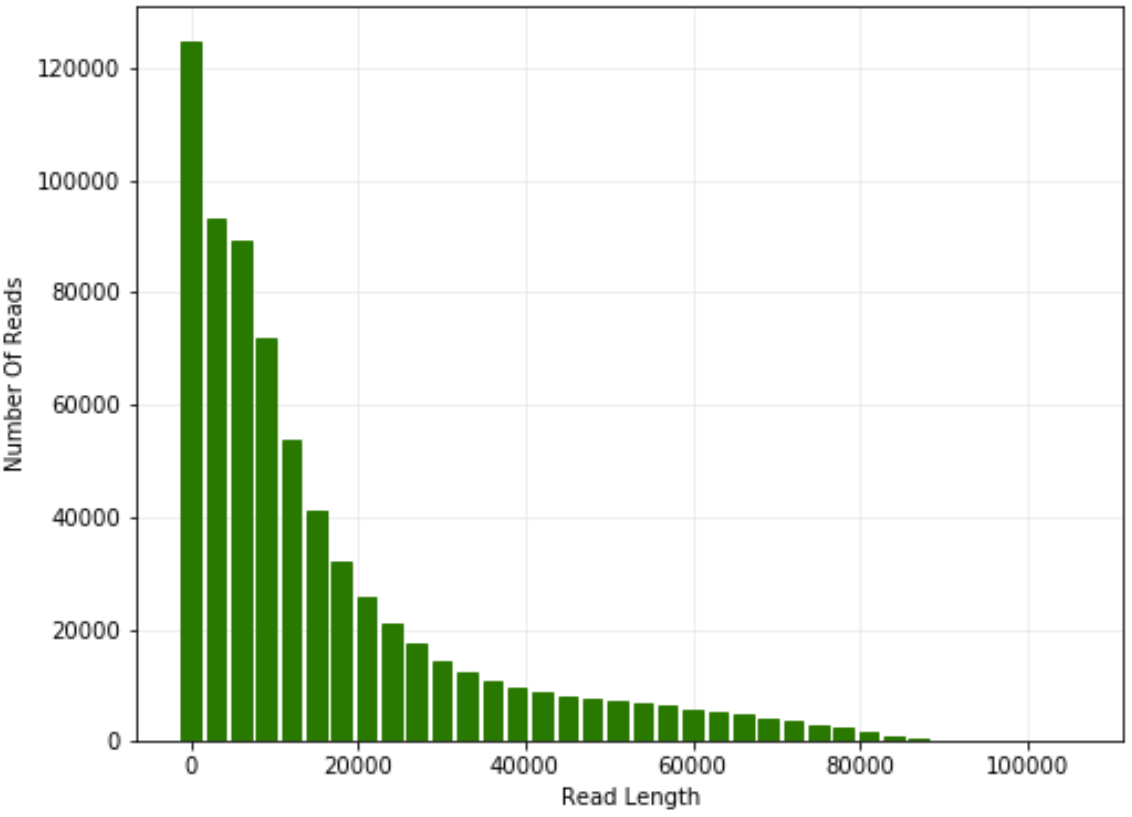

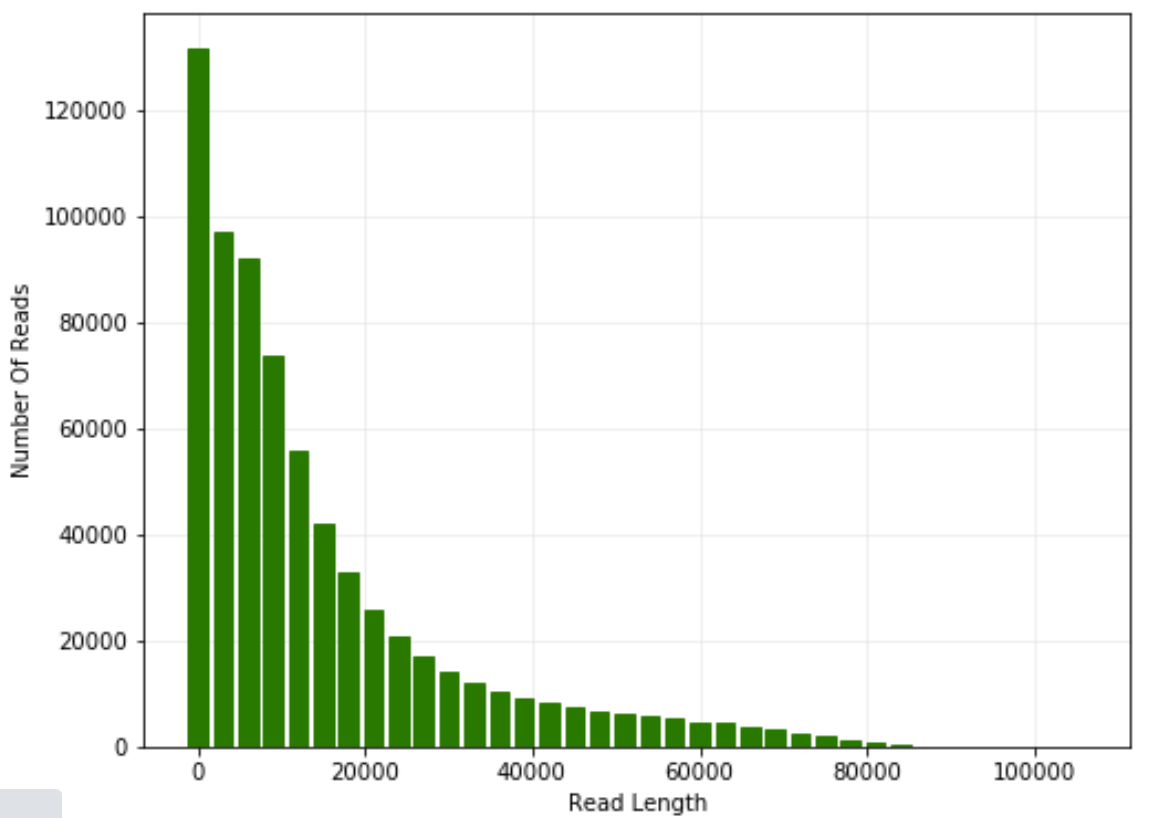


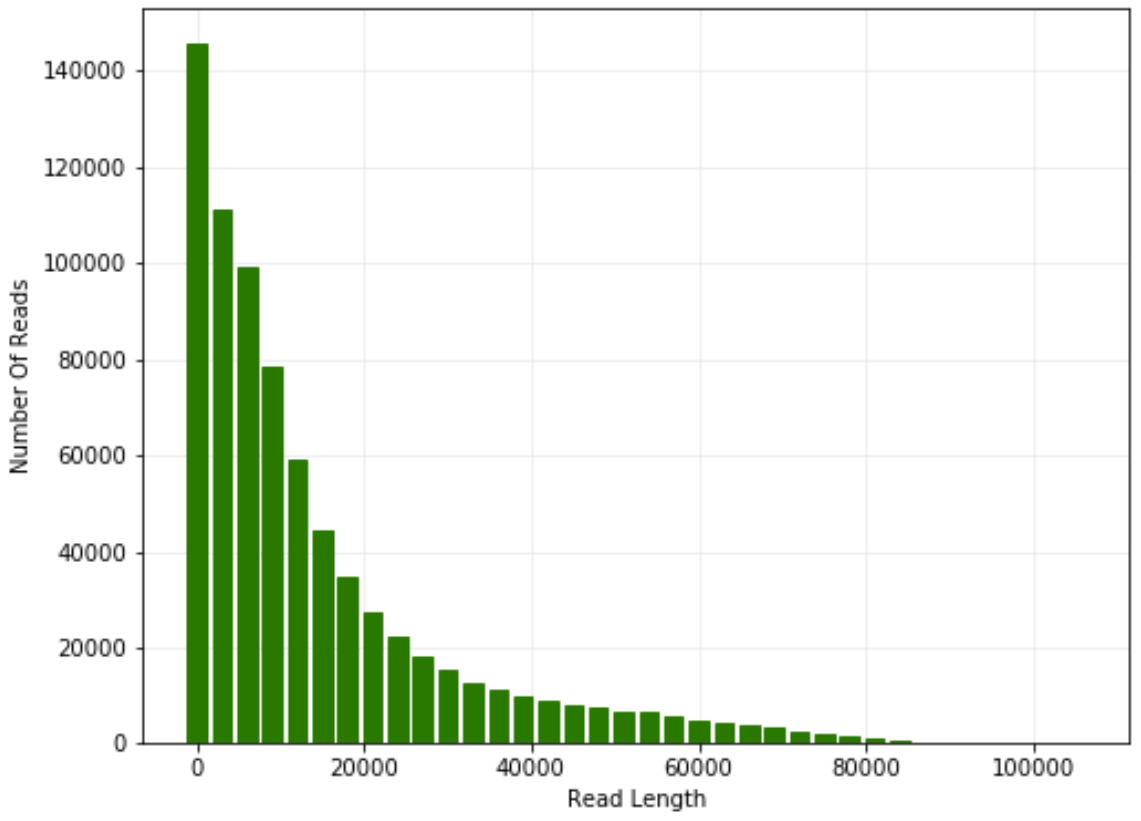


**Fig. S1** Polymerase sequence length distribution in sequencing data.


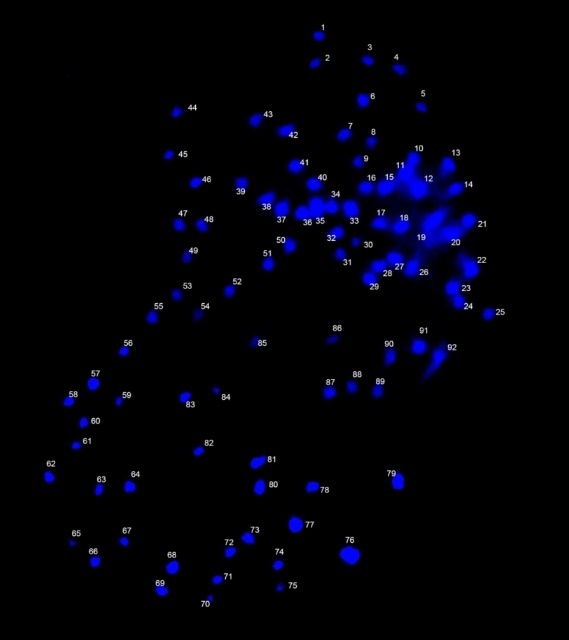

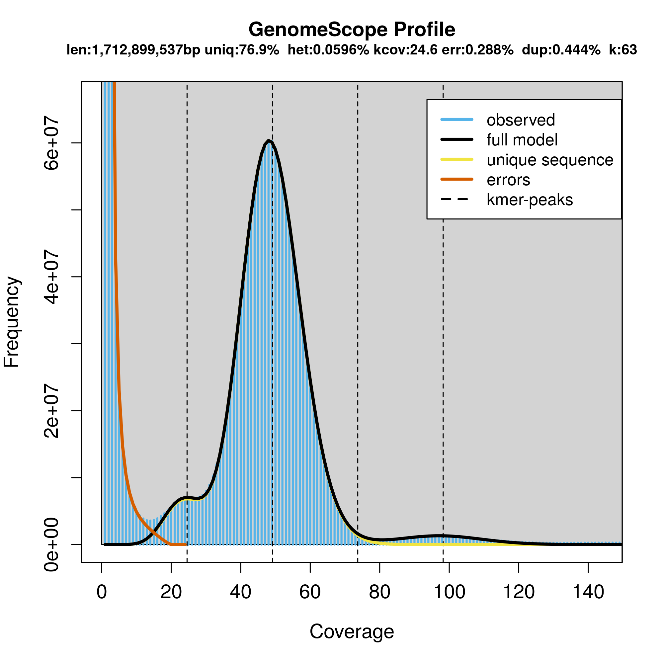


c
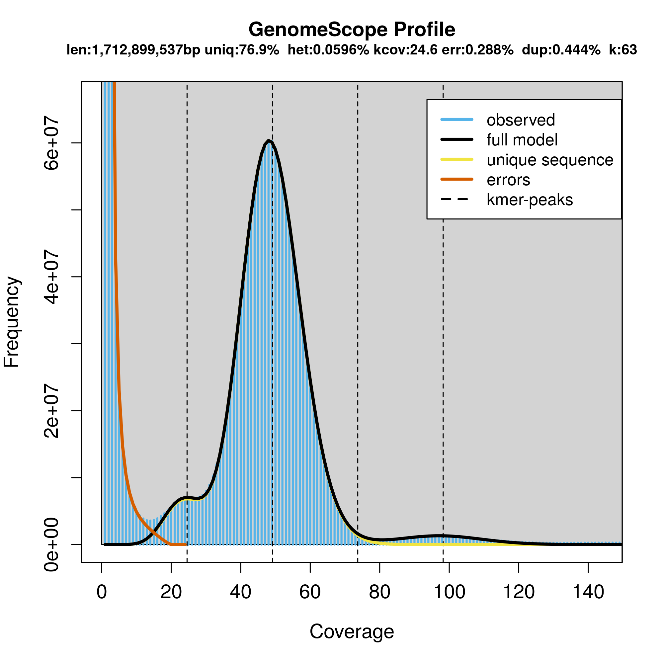


b
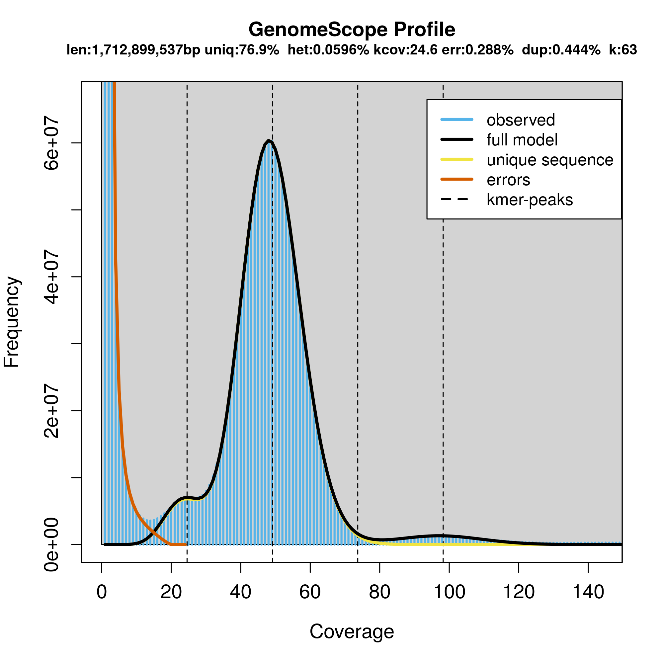


a
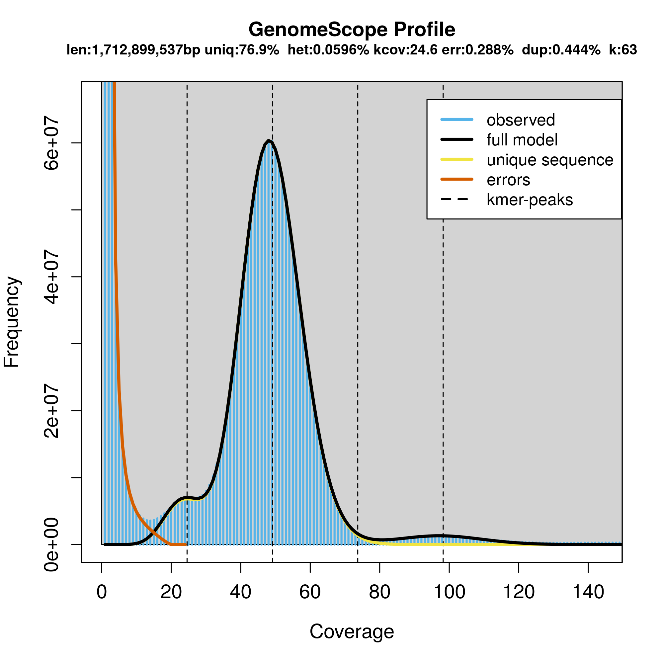


**
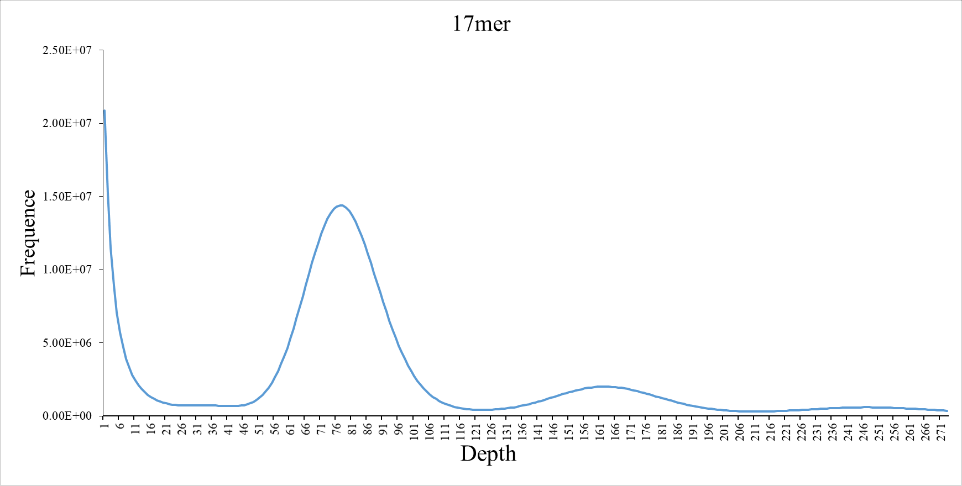
**

**Fig. S2** Ploidy investigation and genome size estimation of *H. hamabo.*

**(a)** The result of Ploidy investigation. **(b)**The result of 17-mer analysis, final genome size(1997.46Mb) was calculated by “genome_size=Total_Base_Pairs/[Kmer_Depth*Read_Length/(Read_Length-Kmer+1)]”. **(c)** Result of 63-mer analysis. After excluding low frequency KMERS due to sequencing errors and very high KMERS, the genome size was calculated to be 1,712,899,537 bp (1.71Gb), the repeat sequence 395,427,260 bp (23%), and the heterozygosity rate was 0.06%.

**
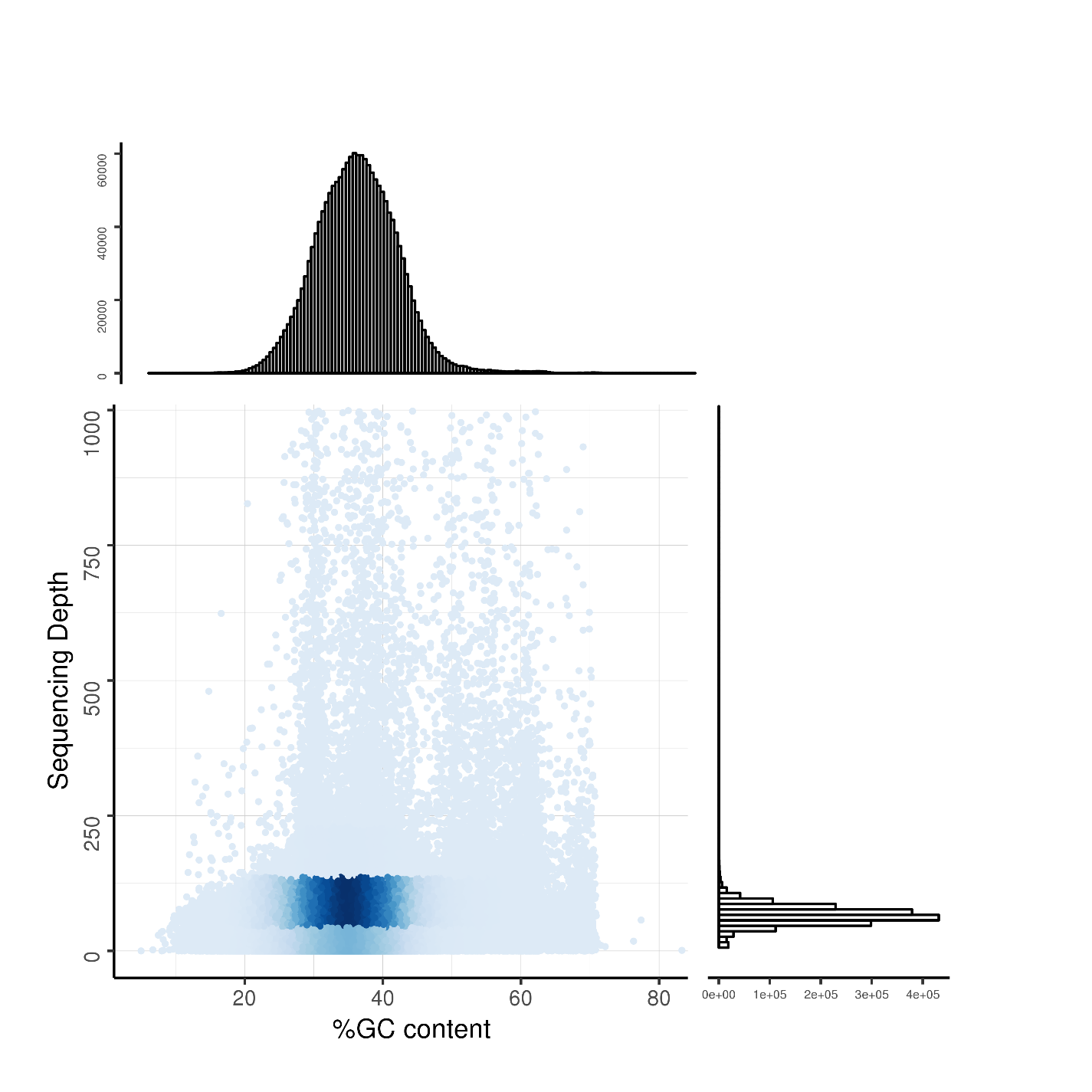
**

**Fig S3** Density map of Contig GC content and sequencing depth distribution

**
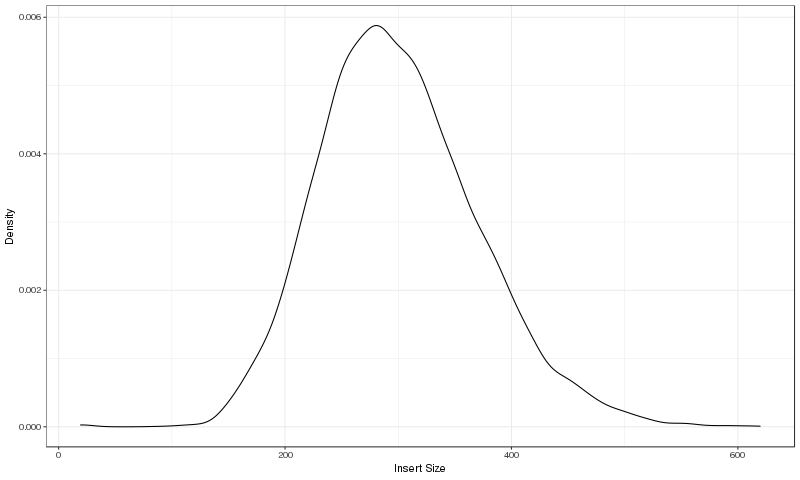
**

**Fig S4** Density map of insert segments distribution.


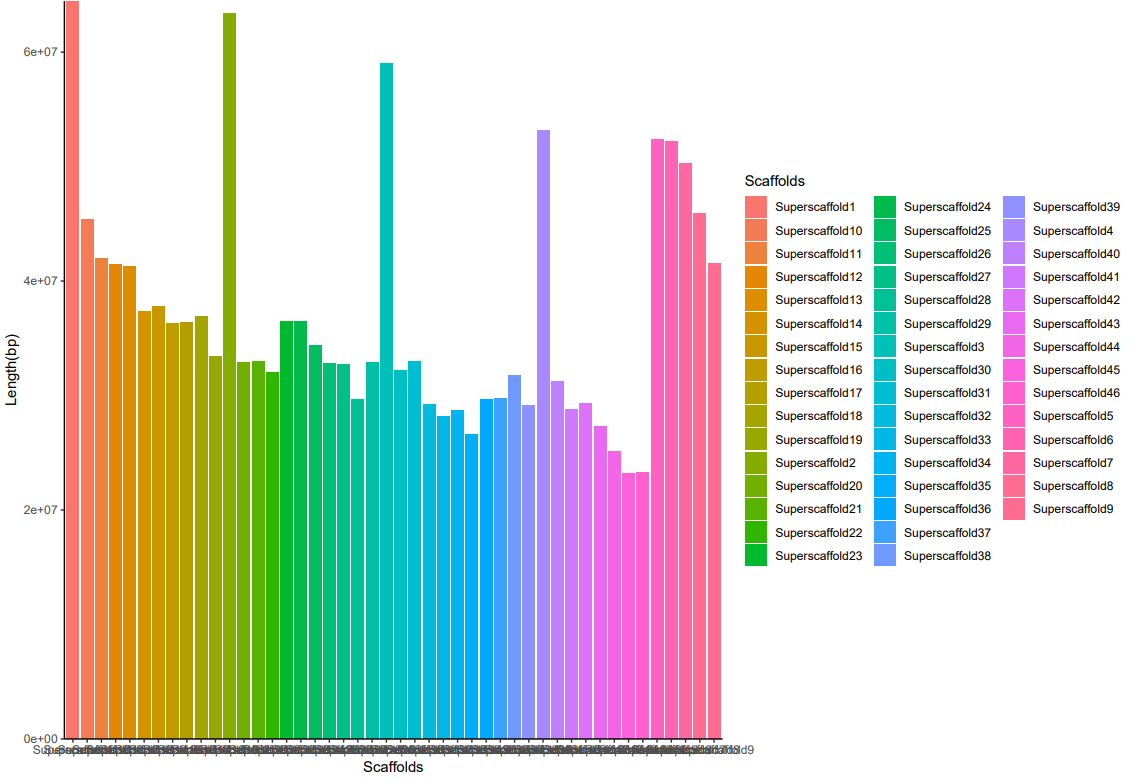


**Fig S5** Forty-six longest pseudomolecules.


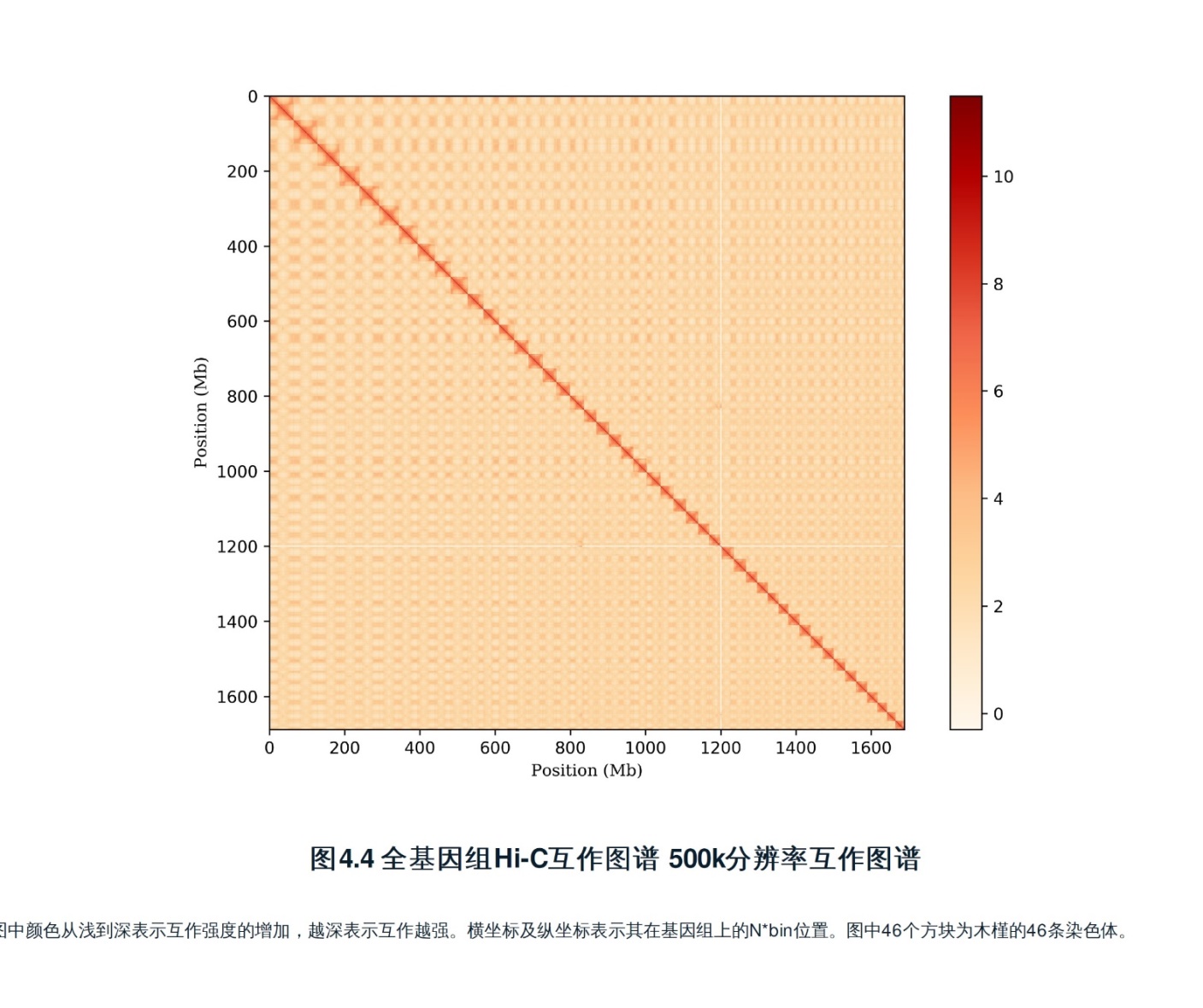


**Fig S6** DNA interactions in 46 *H.hamabo* chromosomes.

Heat map shows a normalized contact matrix, with strong contacts in red and weak contacts in yellow.


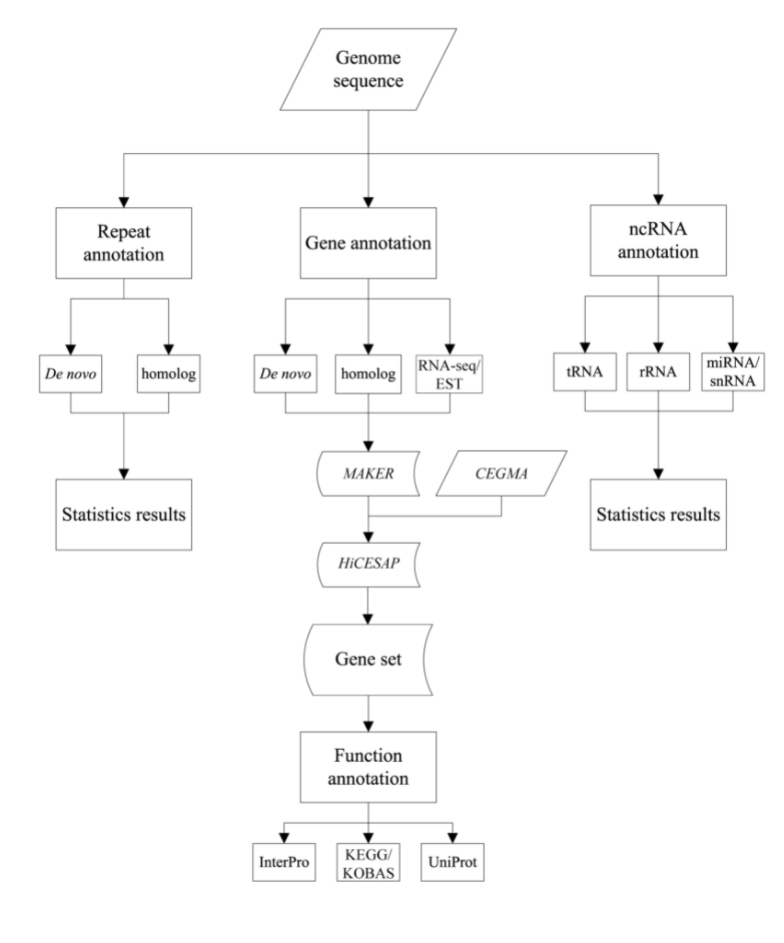


**Fig S7** Genome annotation analysis process.


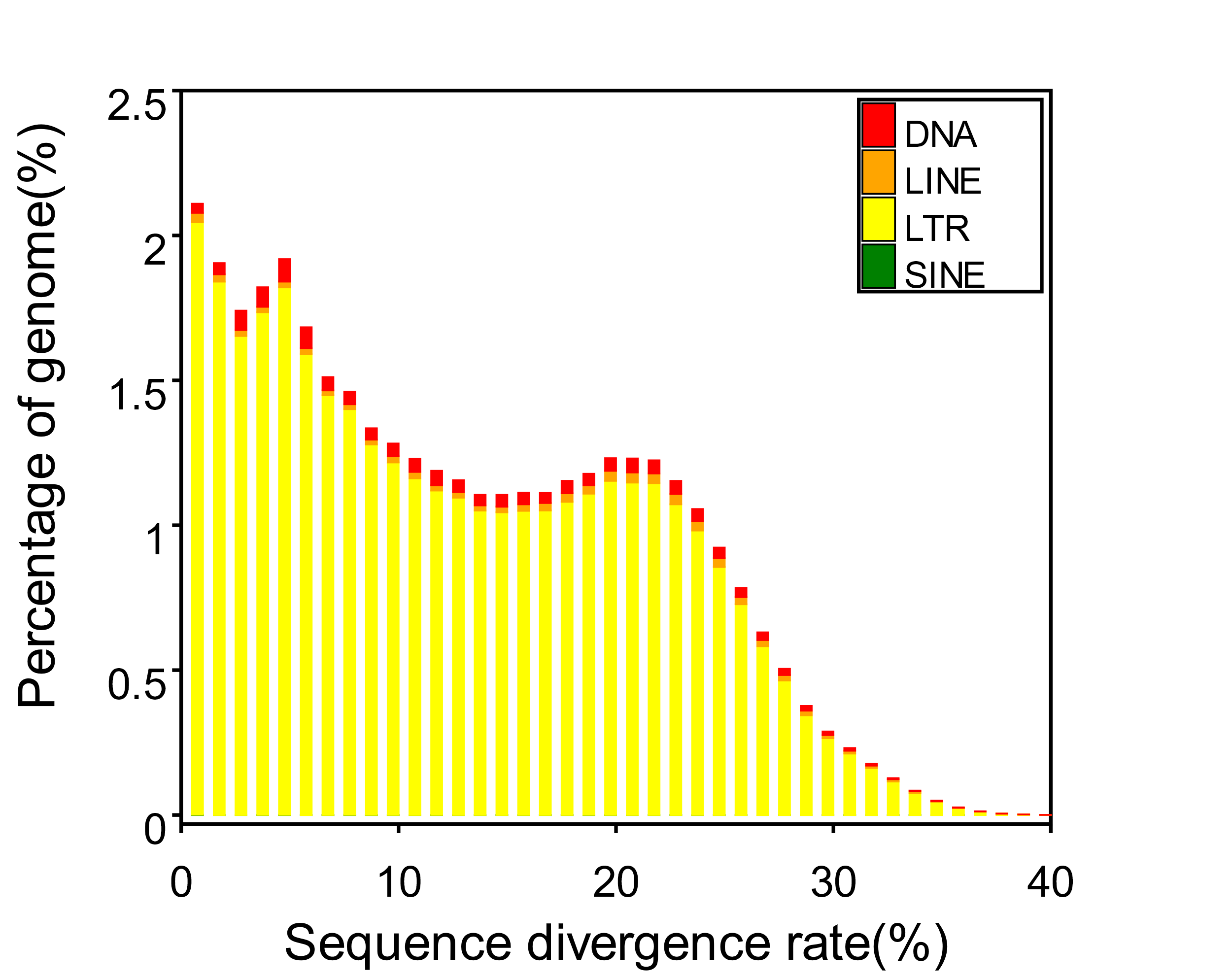


**Fig S8** TE bifurcation degree distribution diagram obtained by REPEATMASKER

**
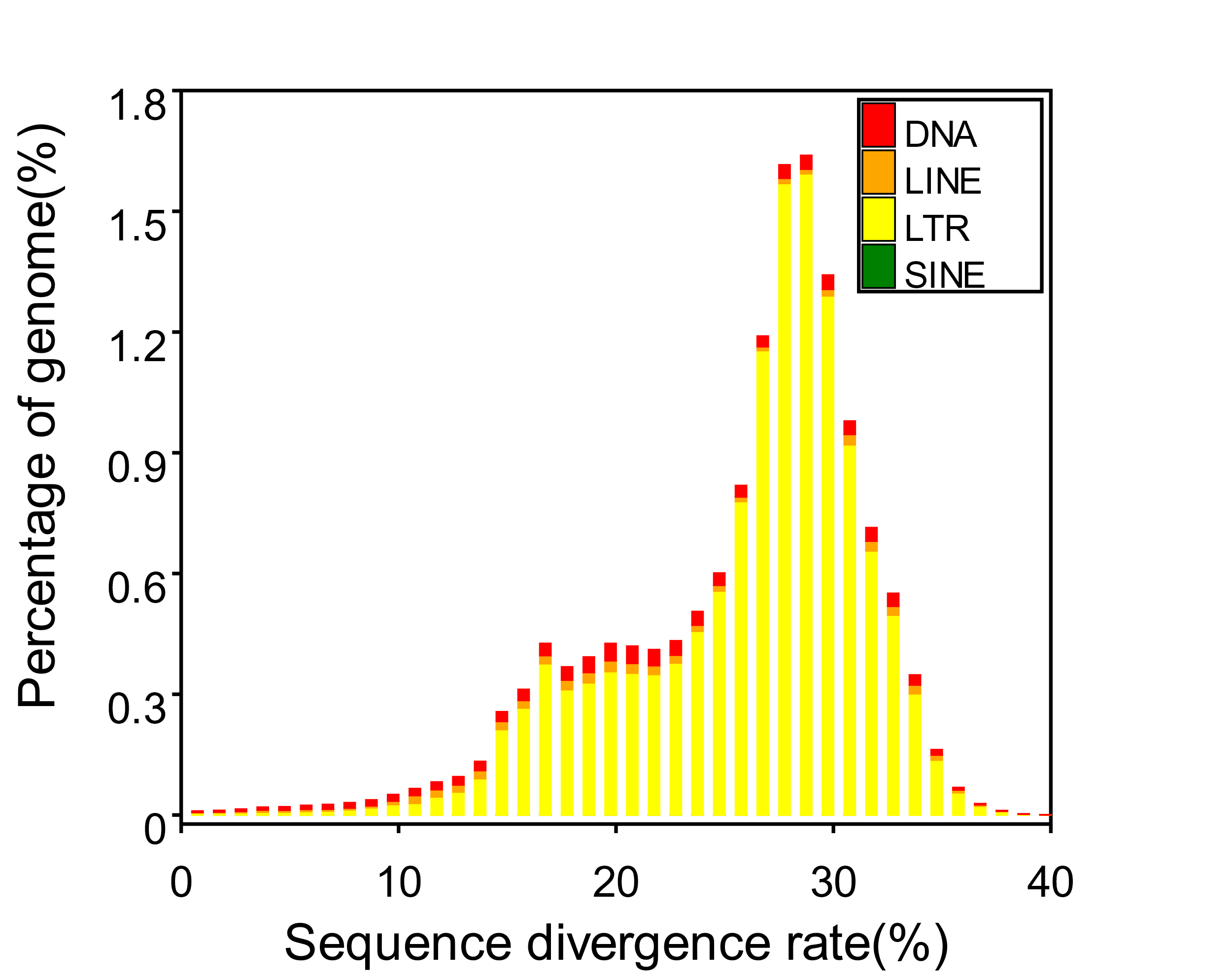
**

**Fig S9** TE bifurcation distribution predicted by the De Novo method.


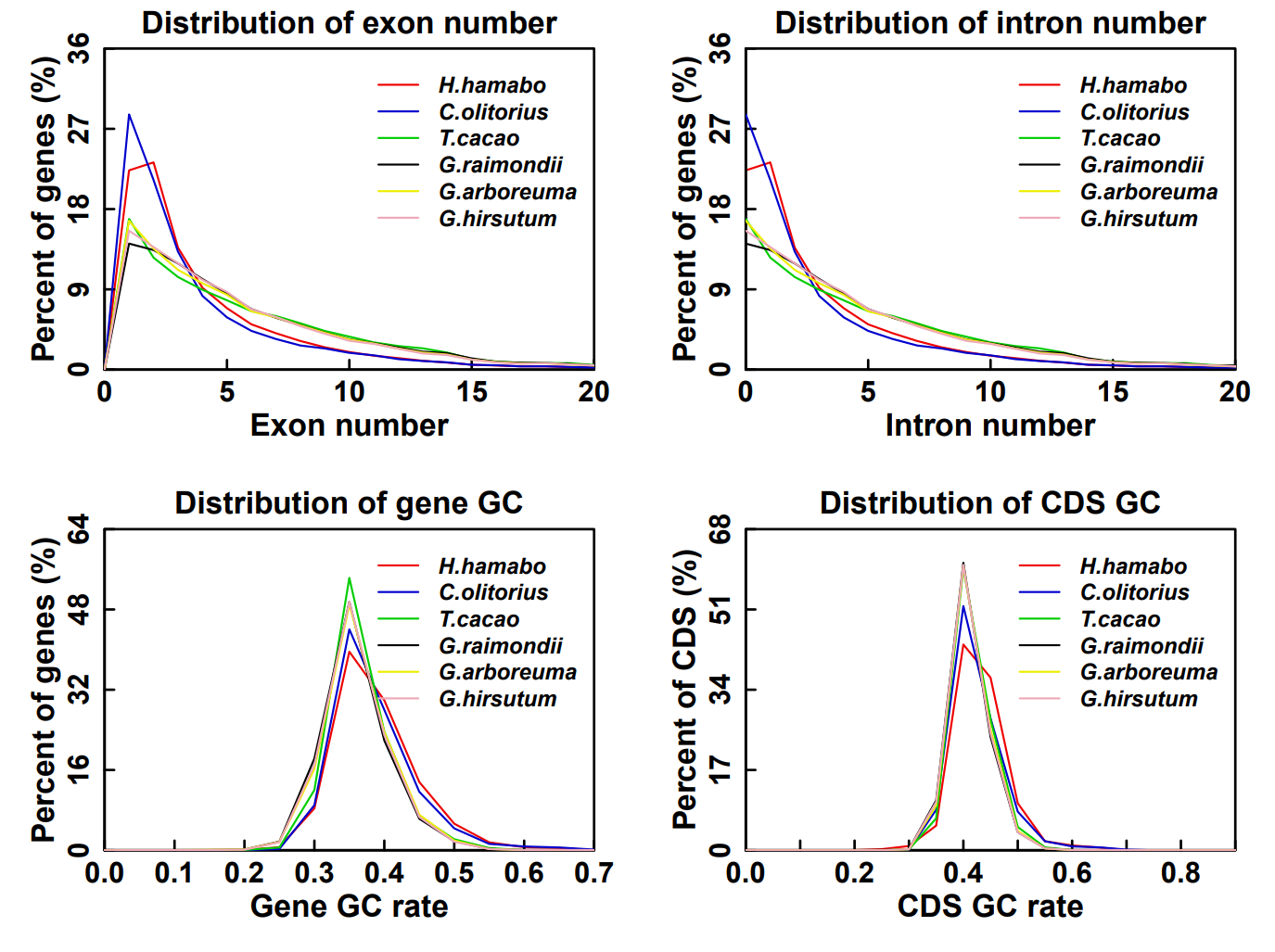


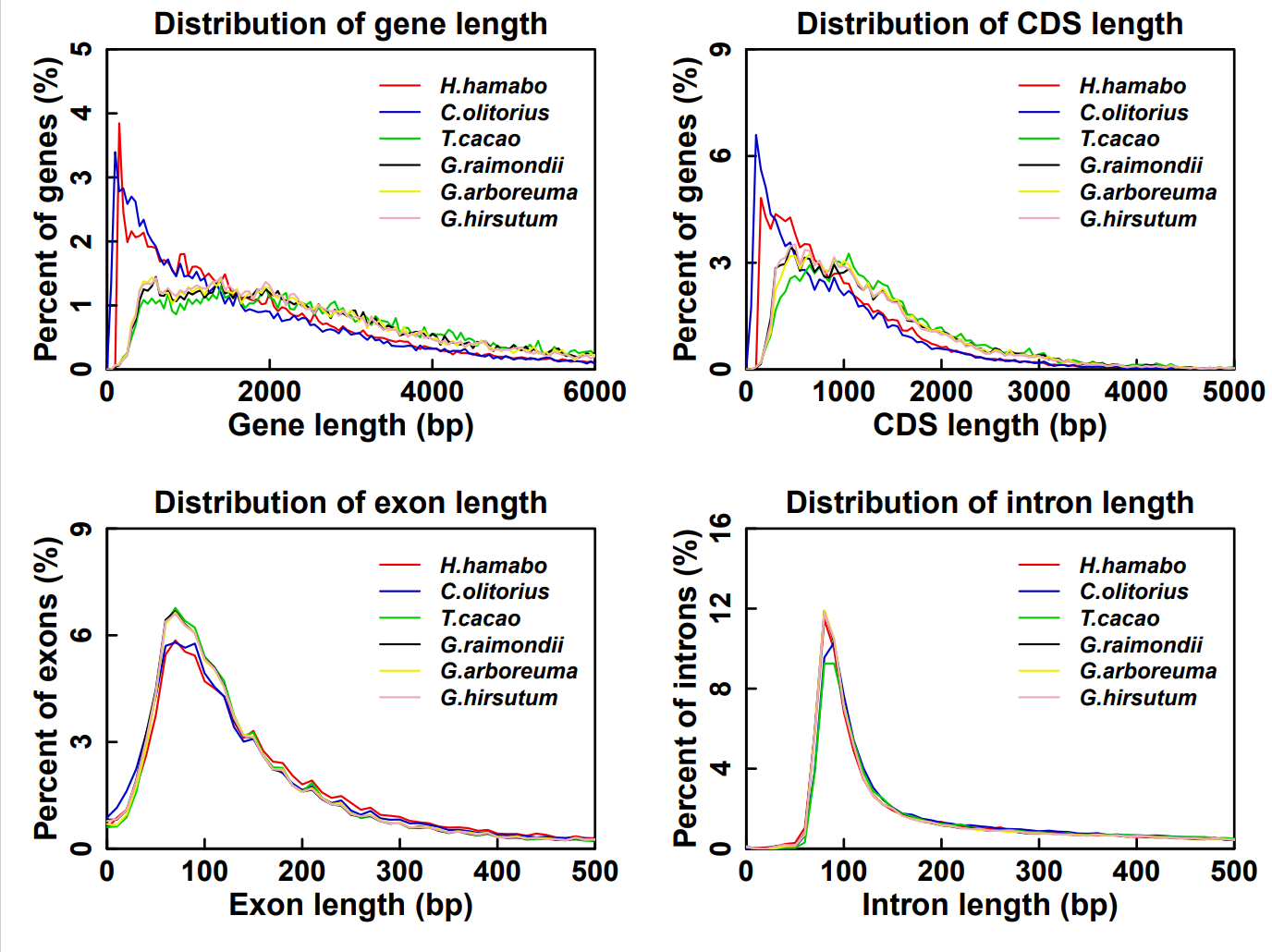


**Fig S10** Gene structure prediction results and gene set statistics


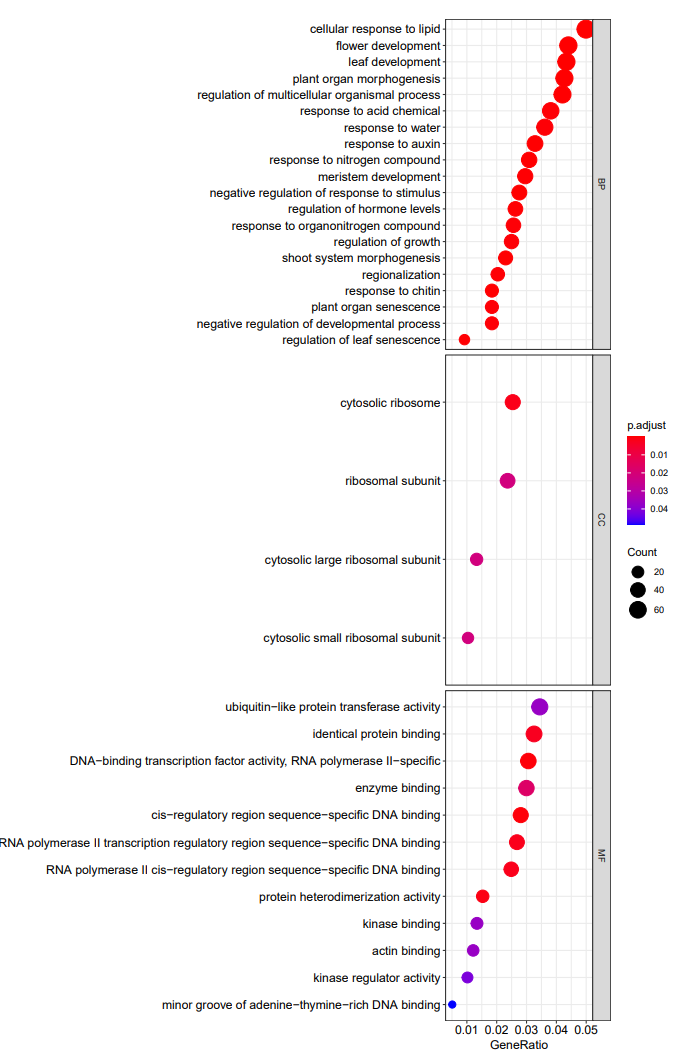


**Fig S11** Enrichment result of unique families


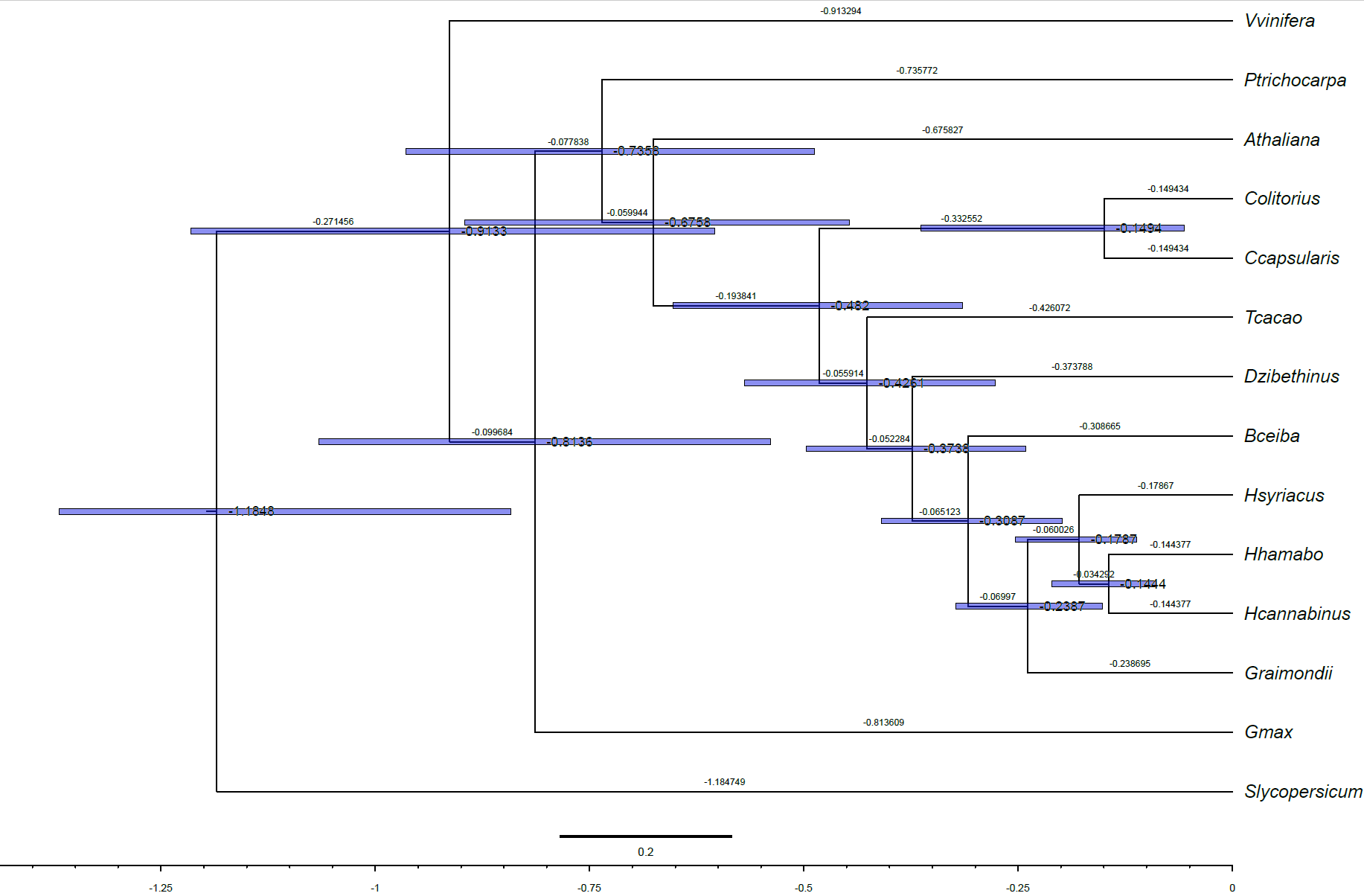


**Fig S12** Evaluation of divergence time


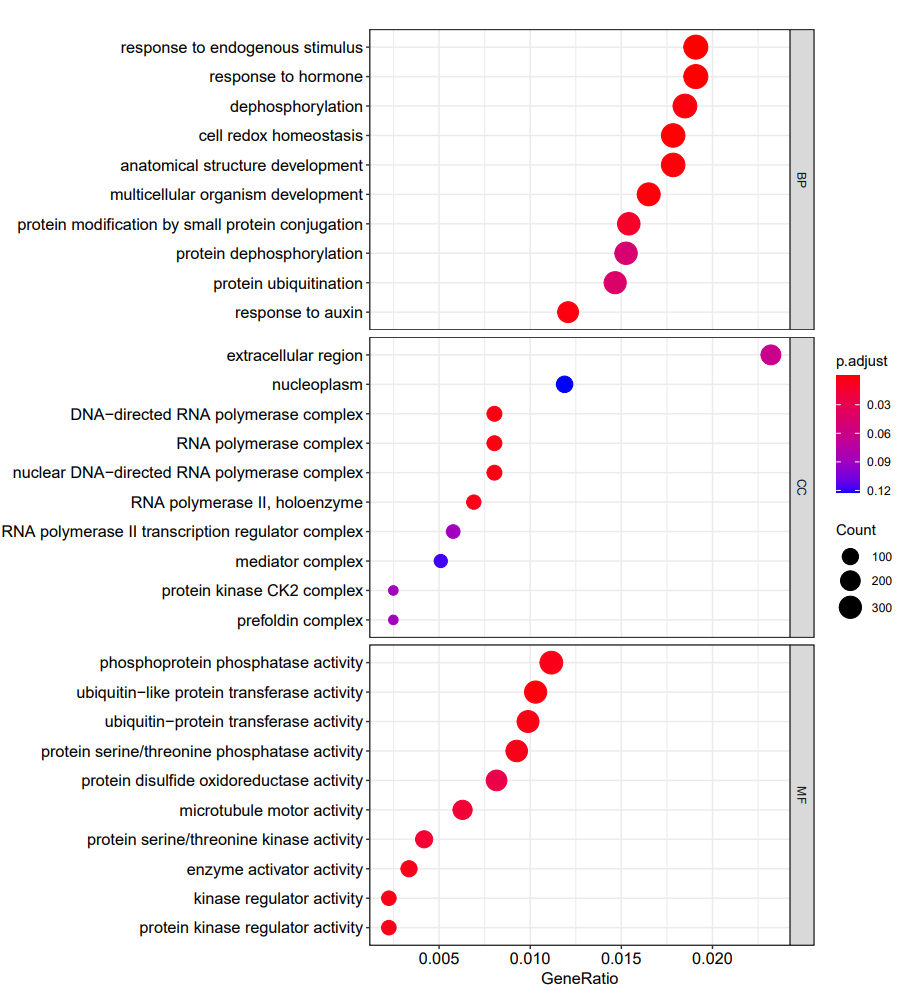


**Fig S13** Enrichment result of significant expansion families.


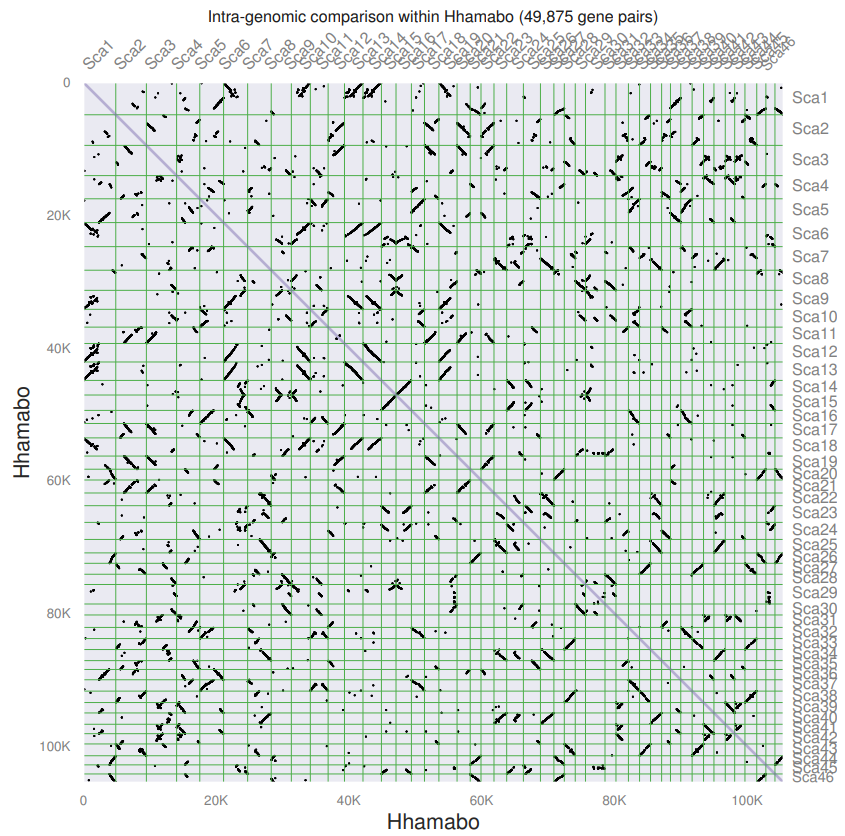


**Fig S14** the intra-genome comparison Dot Plot.


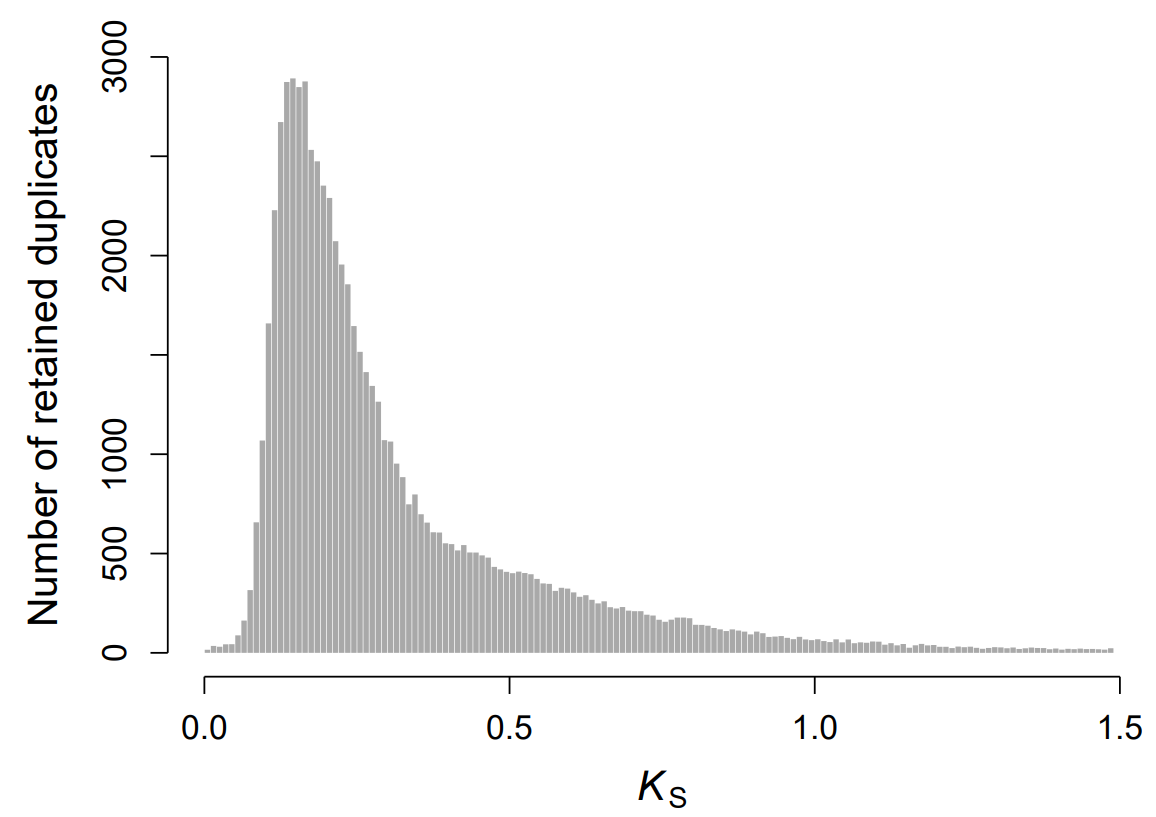

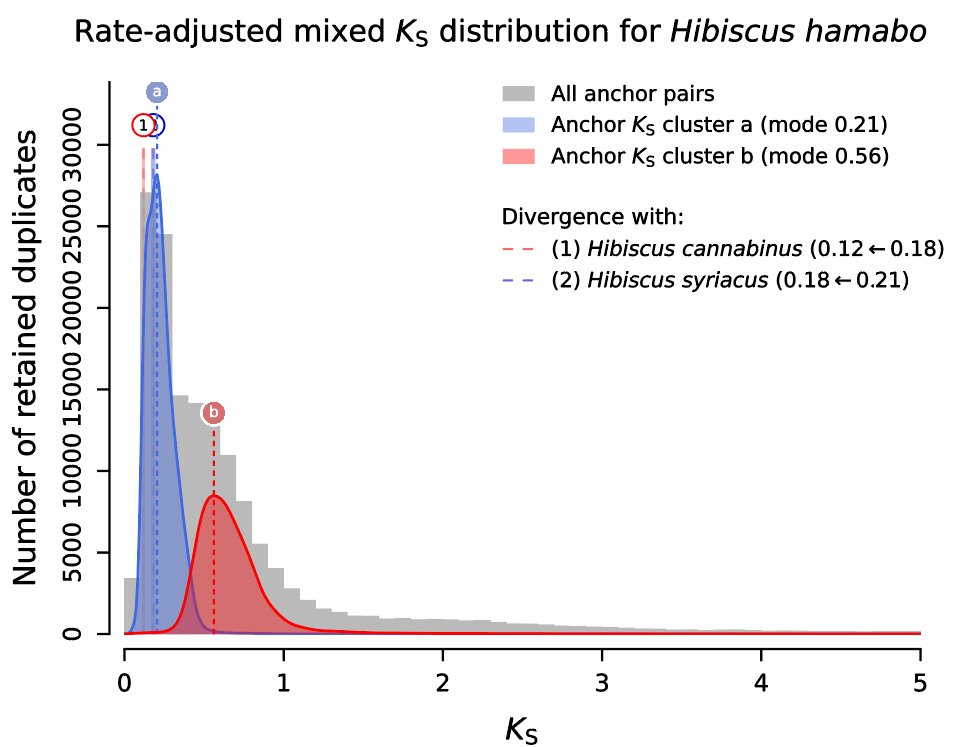

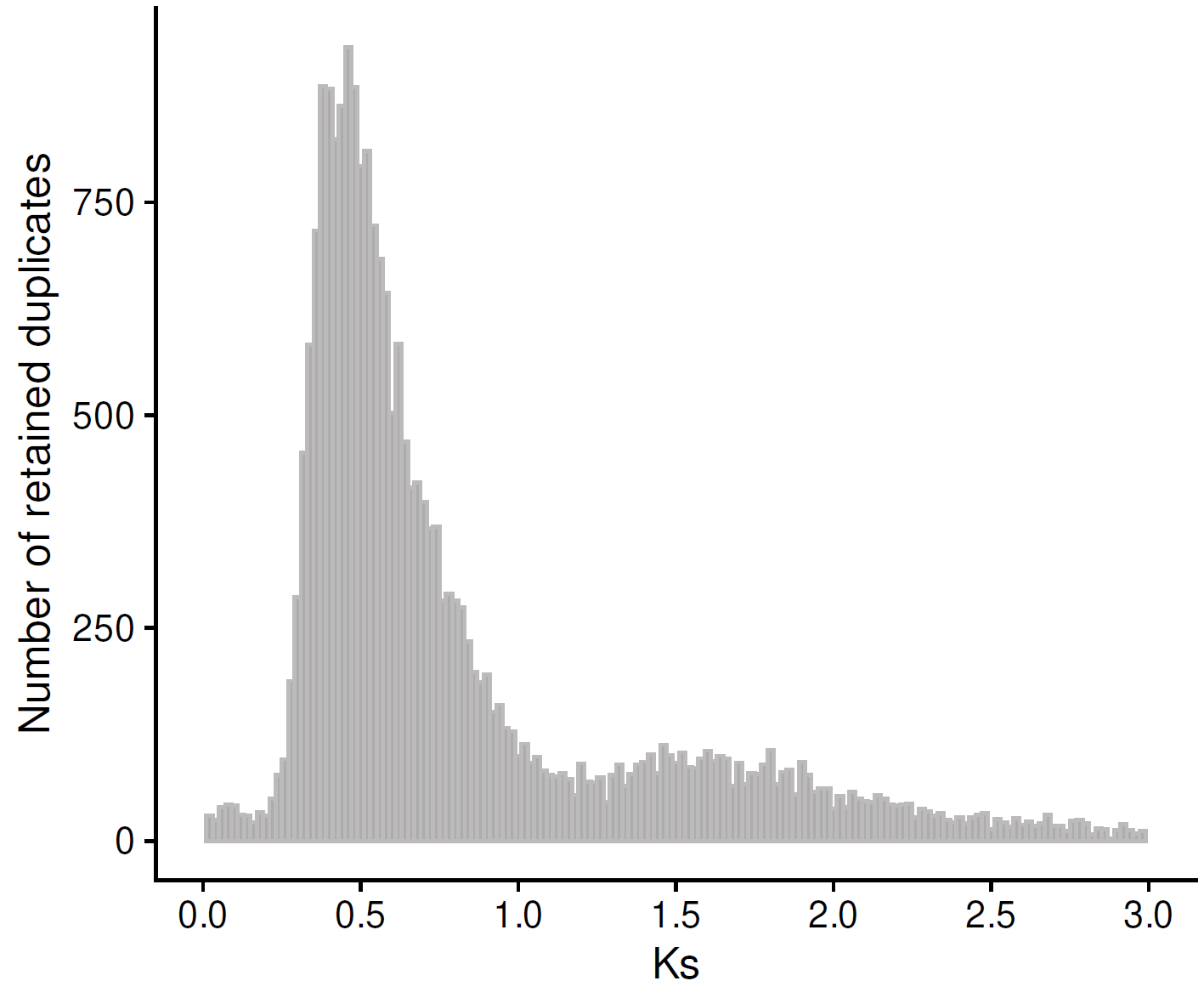

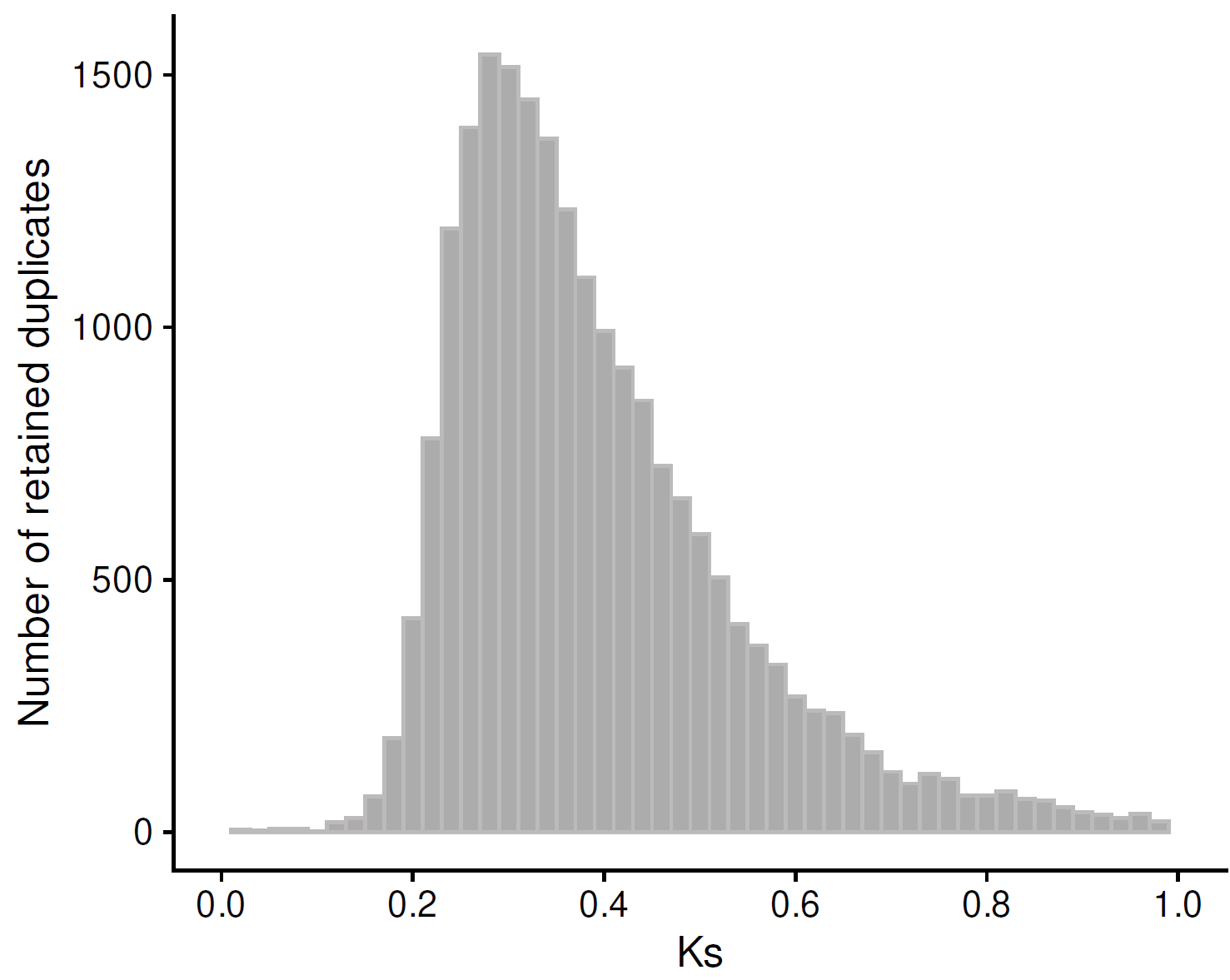


**d**

**c**

**b**

**a**

**Fig S15** ks distribution and rate-ajusting

(a) Paralogs Ks distribution of *H. hamabo*

(b) Rate-adjusted mixed Ks distribution for *H. hamabo* using a reference phylogeny of (*H. syriacus* (*H. cannabinus*, *H. hamabo*)). The dashed lines (blue , red ) indicate two polyploidy events in *H. hamabo*，the solid lines (red , blue ) indicate the divergence events with *H. cannabinus* and *H. syriacus.*

(c) Paralogues Ks distribution of *G. raimondii*

(d) orthologues Ks distribution of *H.* *hamabo* and *G. raimondii*

**b**

**a**


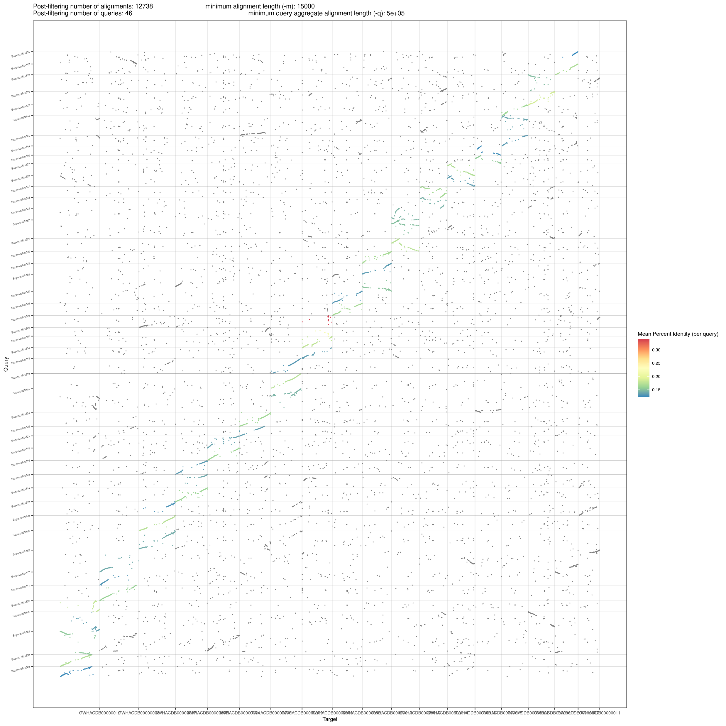

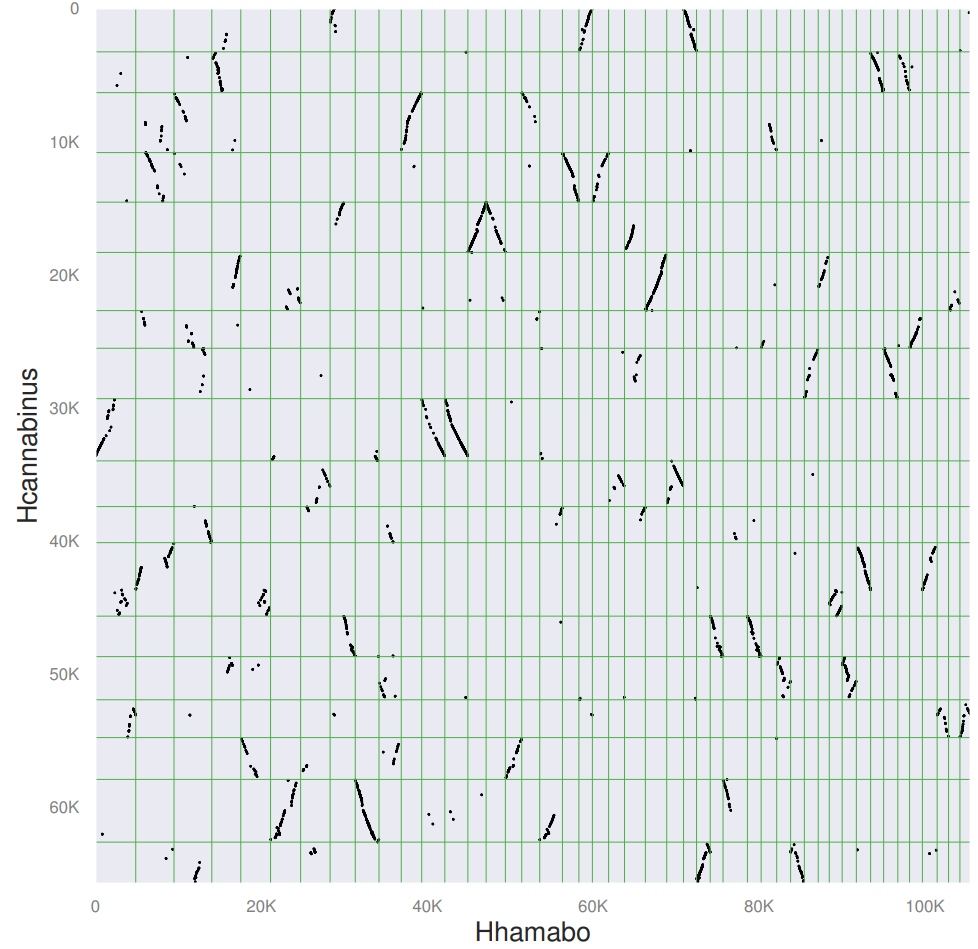

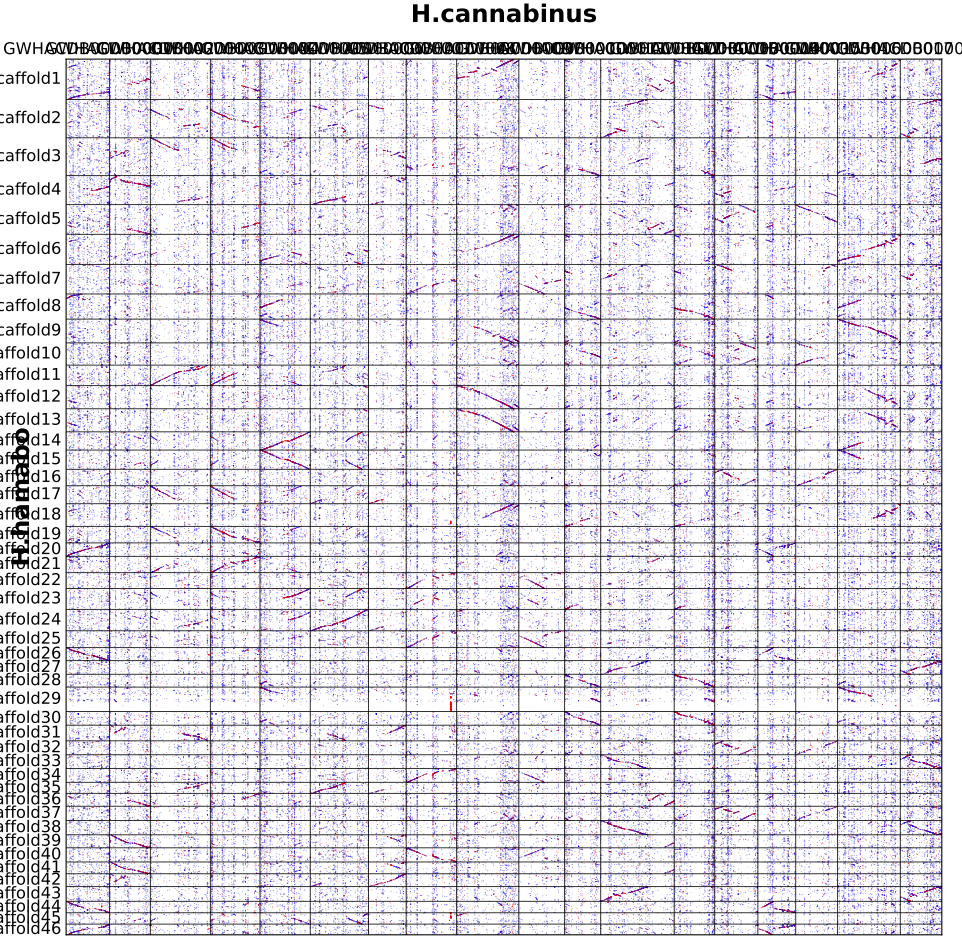

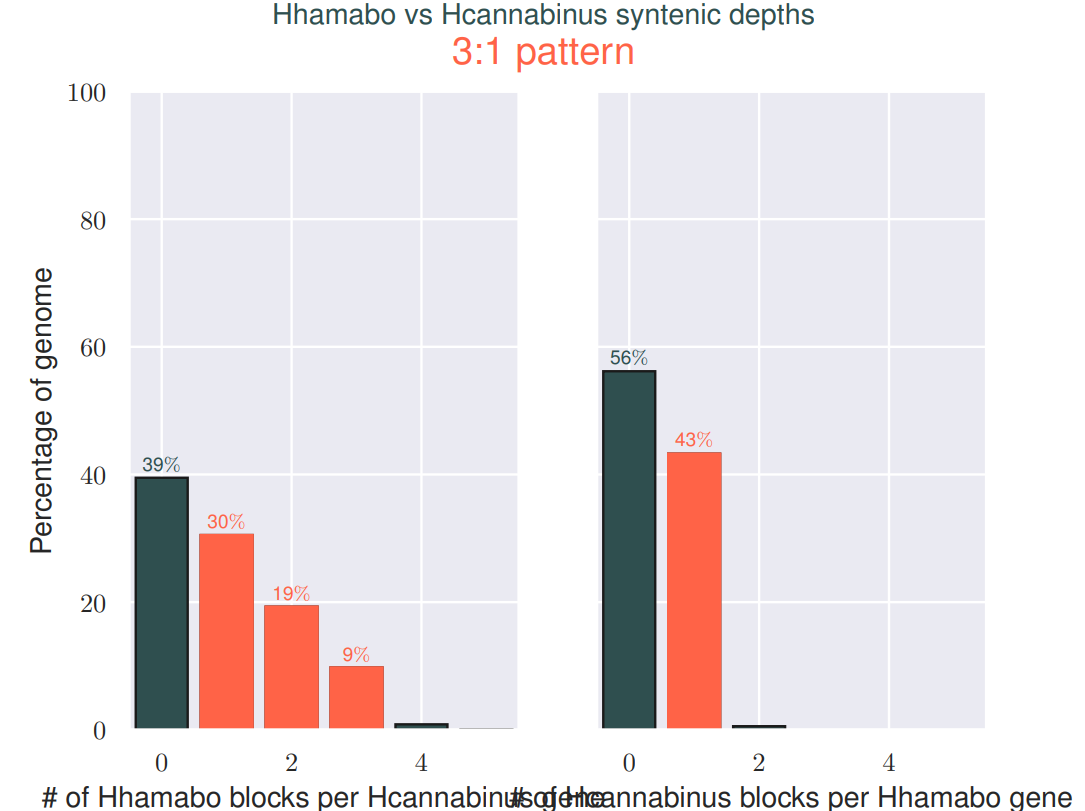


**d**

**c**

**Fig S16** The inter-genome comparison between *H.* *hamabo* and *H.cannabinus.*

(a) The inter-genome comparison between *H.* *hamabo* and *H.cannabinus* using JCVI package.

(b) Syntenic depth show a 3:1 pattern.

(c) The inter-genome comparison between *H.* *hamabo* and *H.cannabinus* using WGDI package.

(d) The inter-genome comparison between *H.* *hamabo* and *H.cannabinus* using minimap package.


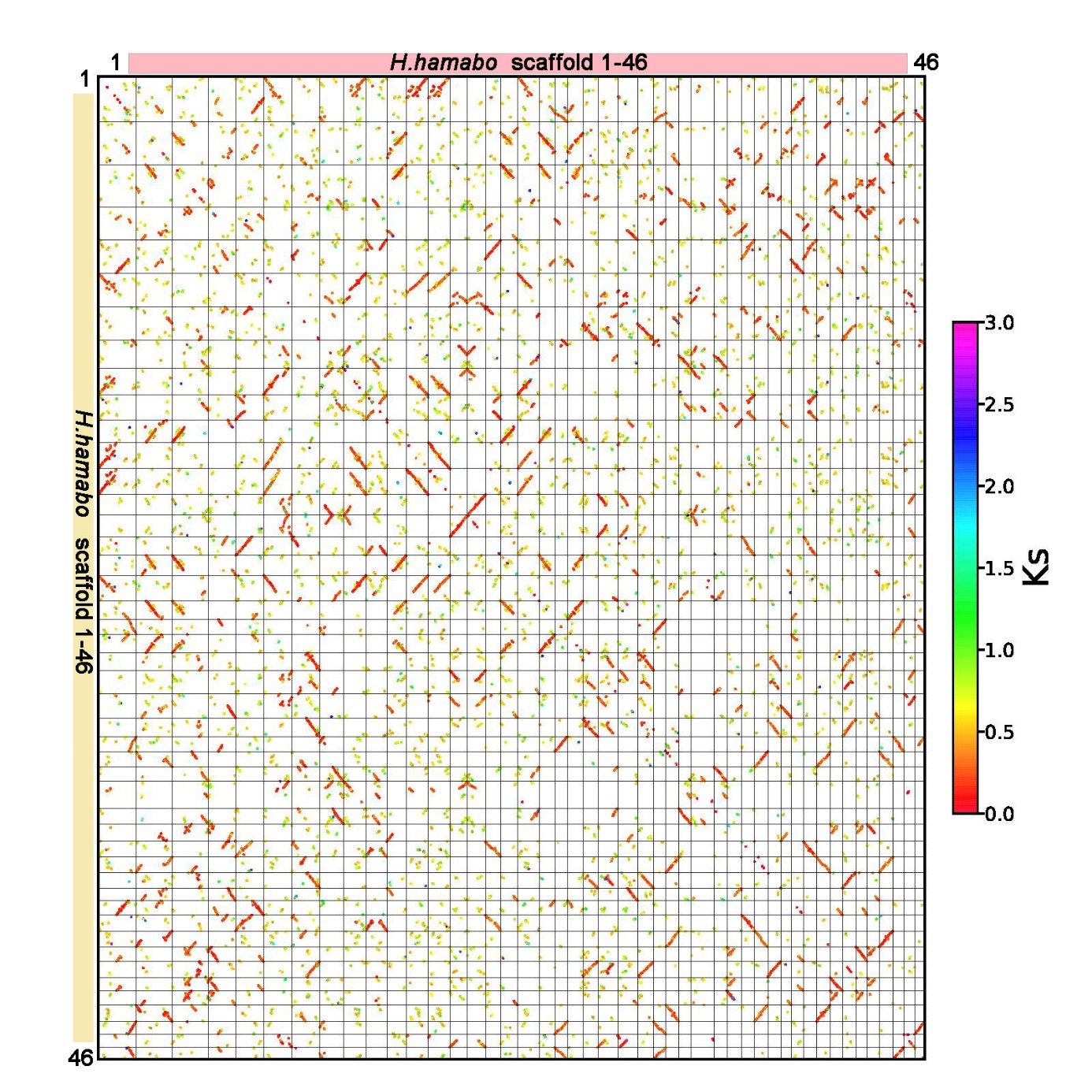


**Fig S17(a)** Ks dotplots of all anchor pairs within *H. hamabo*


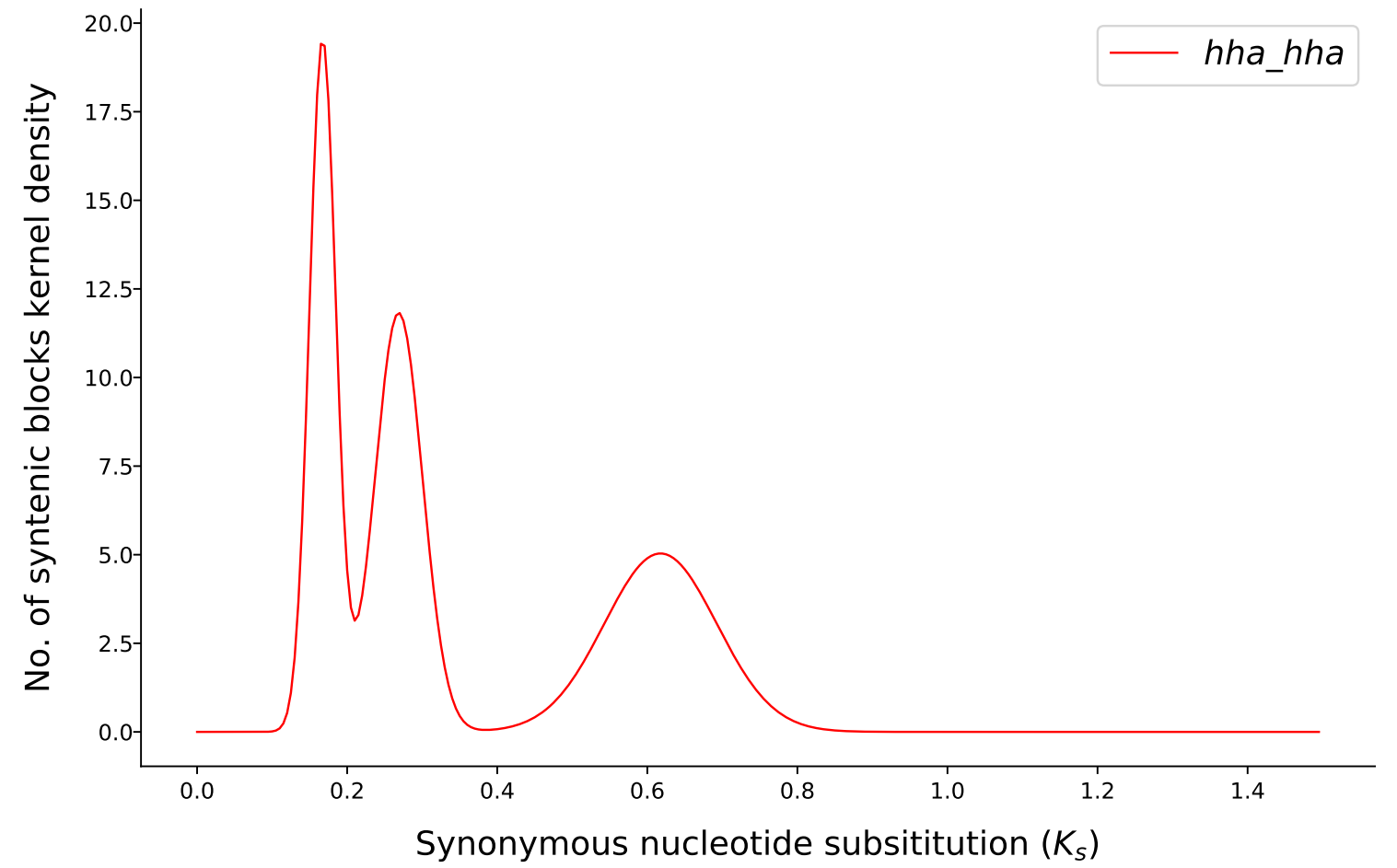

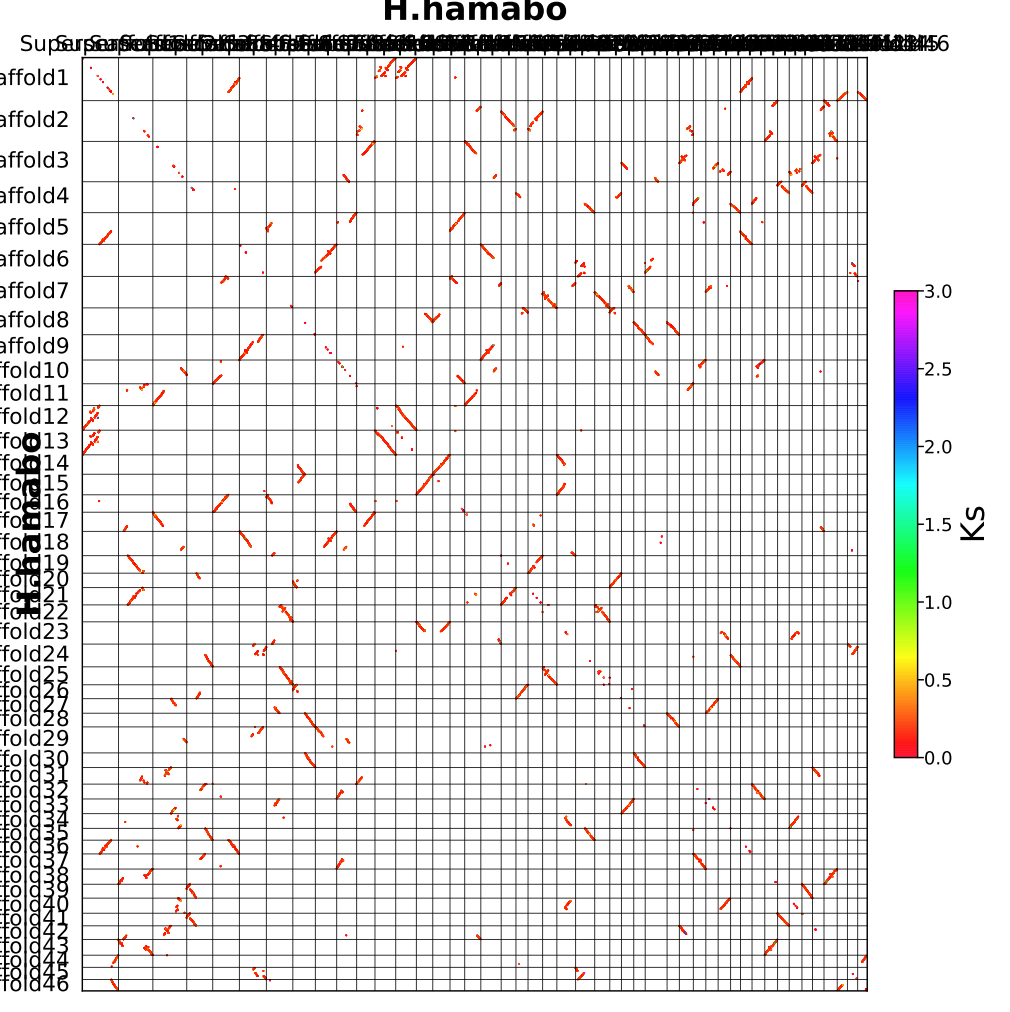

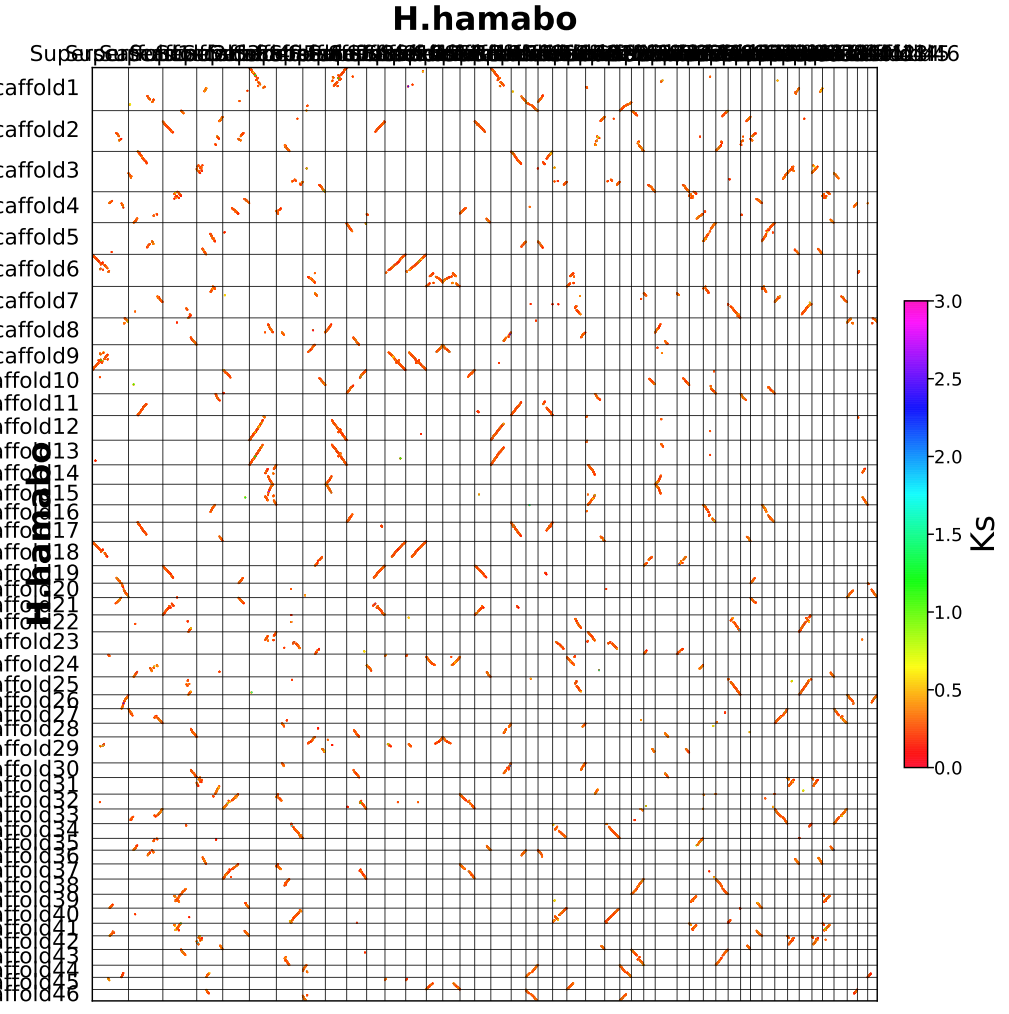


Ks dotplot of peak1

Ks dotplot of peak2

**Fig S17(b)** Ks distribution of all syntenic blocks, with each peak corresponding one Ks dotplot.

**A**


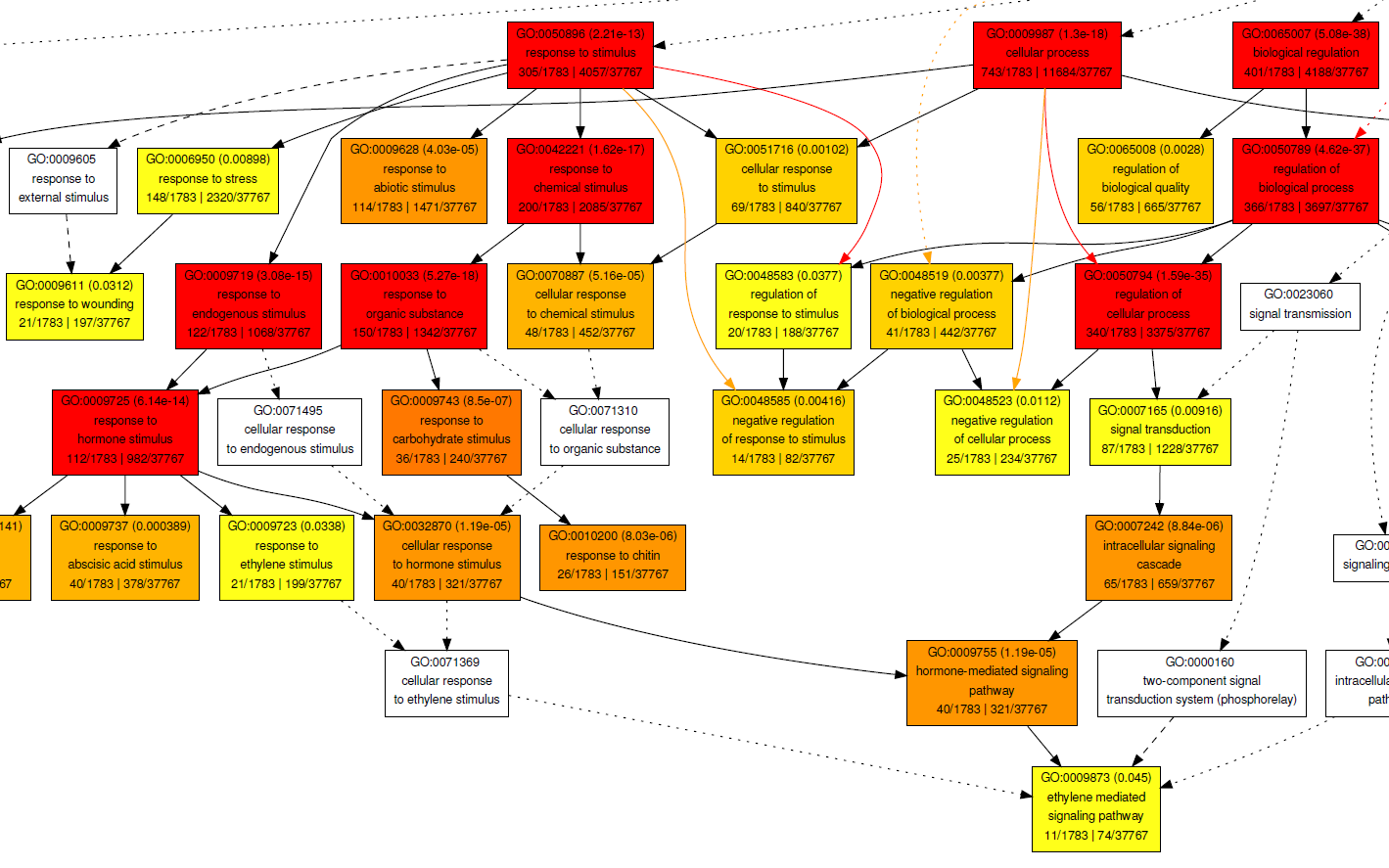


**B**


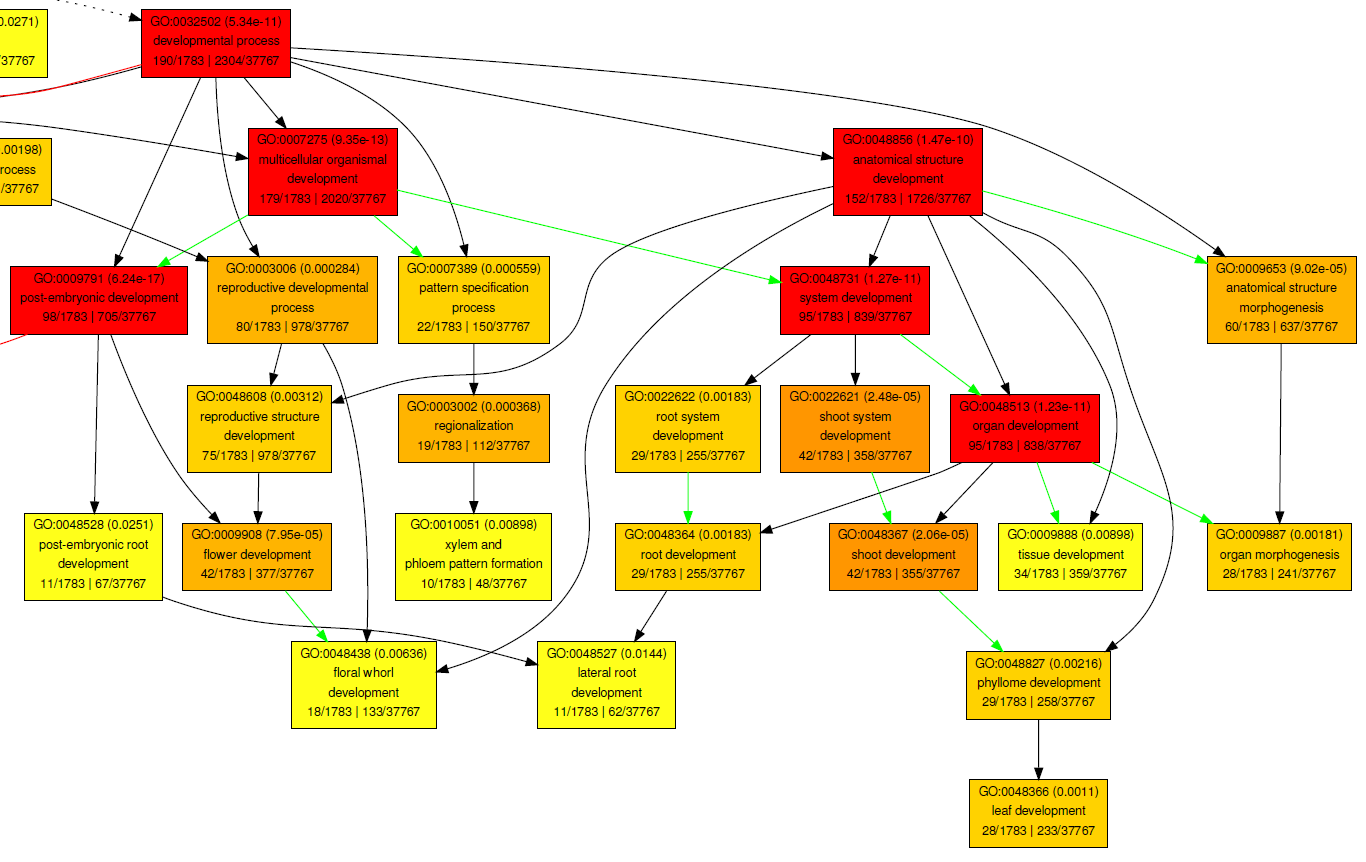


**Fig S18 GO** Enrichment of WGT significant expansion families

(a). A parent GO category: response to stimulus

(b). A parent GO category: developmental process

**
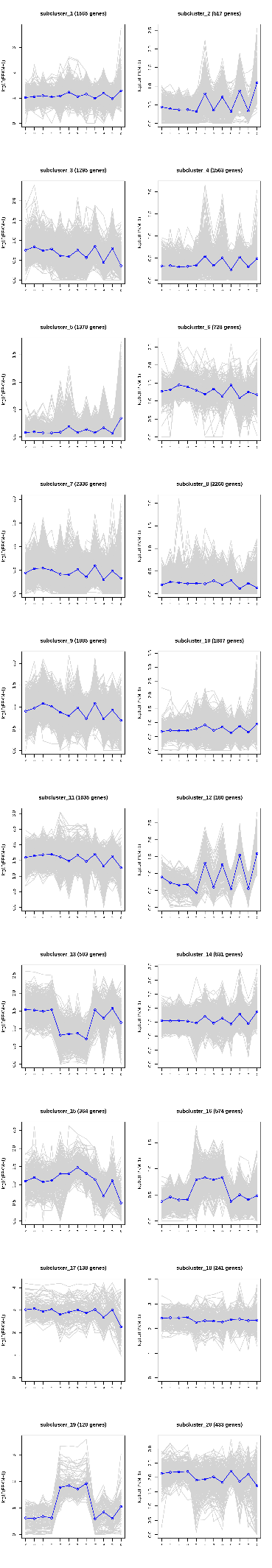

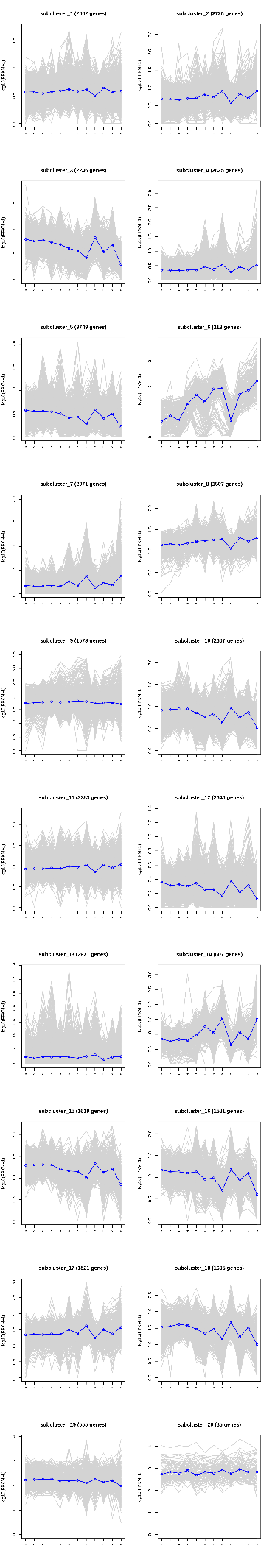
**

Subcluster_1(1665 genes)

Subcluster_2(617 genes)

Subcluster_1(1285 genes)

Subcluster_4(1503 genes)

Subcluster_5(1370 genes)

Subcluster_6(720 genes)

Subcluster_8(2260 genes)

Subcluster_7(2336 genes)

Subcluster_9(1005 genes)

Subcluster_10(1007 genes)

Subcluster_11(1035 genes)

Subcluster_12(180 genes)

Subcluster_13(503 genes)

Subcluster_14(631 genes)

Subcluster_15(364 genes)

Subcluster_16(674 genes)

Subcluster_17(130 genes)

Subcluster_18(241 genes)

Subcluster_19(120 genes)

Subcluster_20(433 genes)

Subcluster_1(2662 genes)

Subcluster_2(2726 genes)

Subcluster_1(2246 genes)

Subcluster_4(2025 genes)

Subcluster_5(3748 genes)

Subcluster_6(213 genes)

Subcluster_8(1007 genes)

Subcluster_7(2071 genes)

Subcluster_9(1573 genes)

Subcluster_10(2007 genes)

Subcluster_11(3203 genes)

Subcluster_12(2646 genes)

Subcluster_13(2971 genes)

Subcluster_14(607 genes)

Subcluster_15(1610 genes)

Subcluster_16(1501 genes)

Subcluster_17(1521 genes)

Subcluster_18(1606 genes)

Subcluster_19(555 genes)

Subcluster_20(05genes)

**Fig S19(left)** All differentially expressed genes (DEGs) in leaves were classified into 20 clusters according to their expression patterns. The X axis represent “log10(FPKM^-1^)” ranking from 0-3.0, with 0.5 per unit, The Y axis represent 12 leaf samples with different treatments: A (5 min-CK-L), C (5 min-S-L), E (5 min-W-L), G (5 min-SW-L), I (9 h-CK-L), K (9 h-S-L), M (9 h-W-L), O (9 h-SW-L), Q (3 d-CK-L), S (3 d-S-L), U (3 d-W-L), W (3 d-SW-L). Time: 5 min, 9 h, 3 d. Treatment: CK (control), S (3.5% NaCl solution), W (waterlogging), SW (NaCl+ waterlogging), Organs**:** L(leaf).

**Fig S20(right)** All differentially expressed genes (DEGs) in roots were classified into 20 clusters according to their expression patterns. The X axis represent “log10(FPKM-1)” ranking from 0-3.0, with 0.5 per unit, The Y axis represent 12 root samples with different treatments: B (5 min-CK-R), D (5 min-S-R), F (5 min-W-R), H (5 min-SW-R), J (9 h-CK-R), L (9 h-S-R), N (9 h-W-R), P (9 h-SW-R), R (3 d-CK-R), T (3 d-S-R), V (3 d-W-R), X (3 d-SW-R). Time: 5 min, 9 h, 3 d. Treatment: CK (control), S (3.5% NaCl solution), W (waterlogging), SW (NaCl+ waterlogging), Organs: R(root).

**
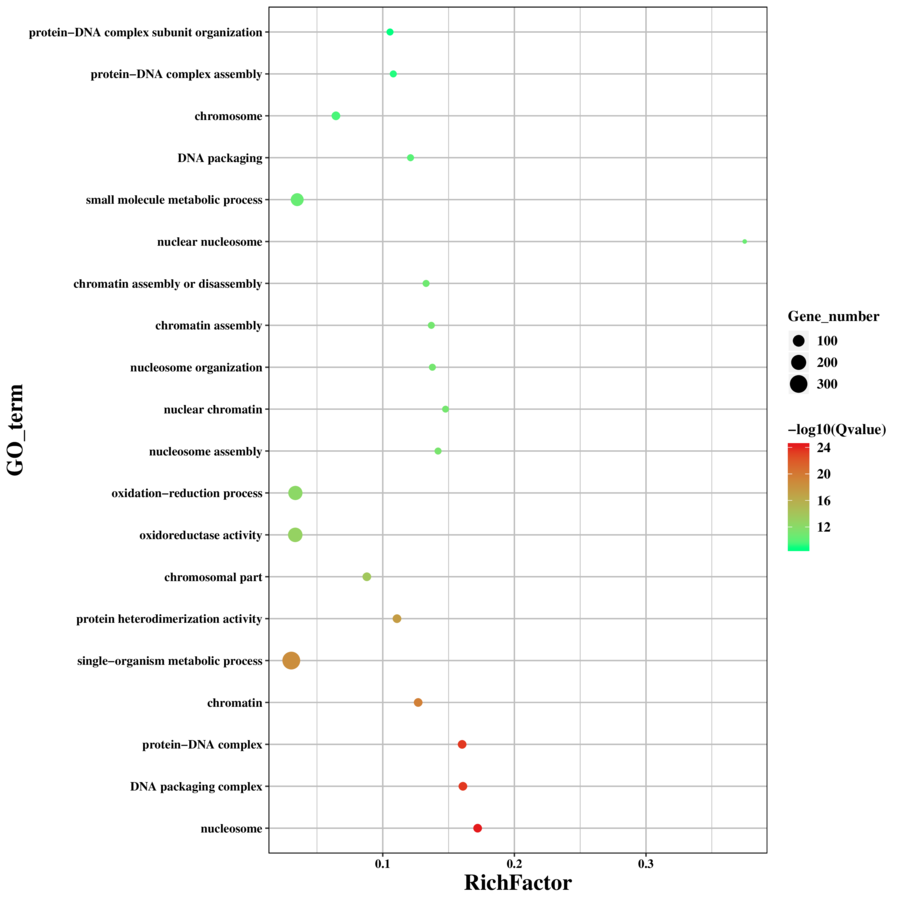
**

**Fig S21** GO enrichment result of DEGs belonging to Subcluster 11 (Fig S20) in roots.


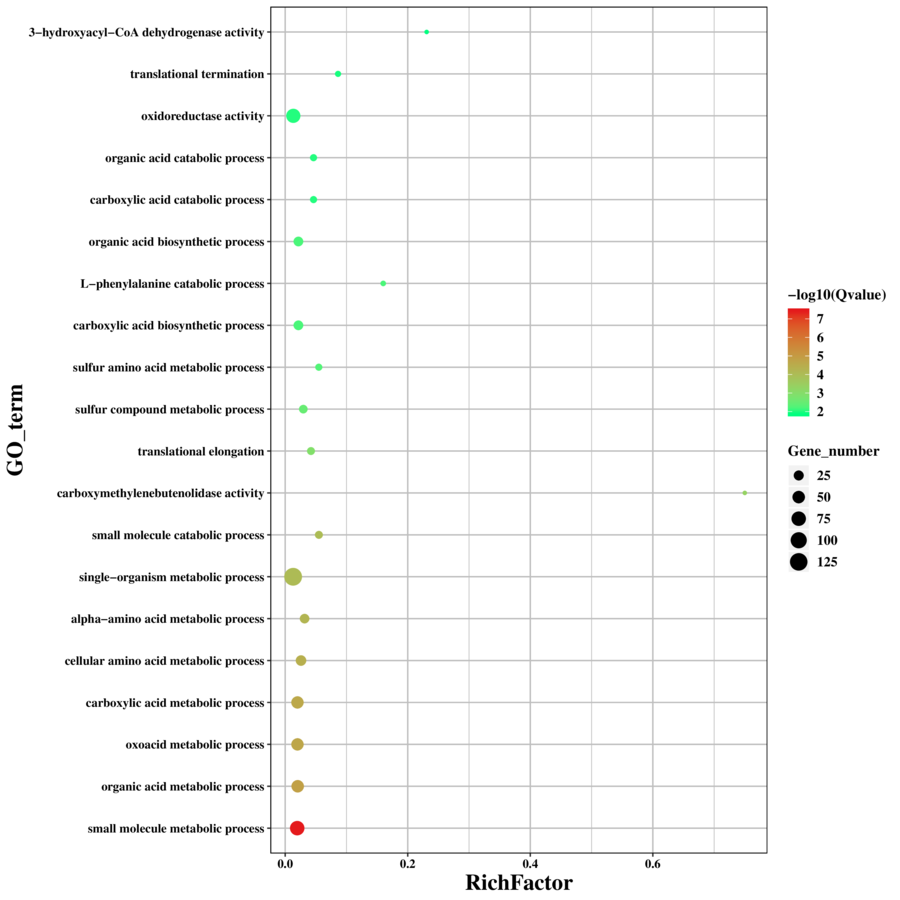


**Fig S22** GO enrichment result of DEGs belonging to Subcluster 18 (Fig. S19) in leaves.


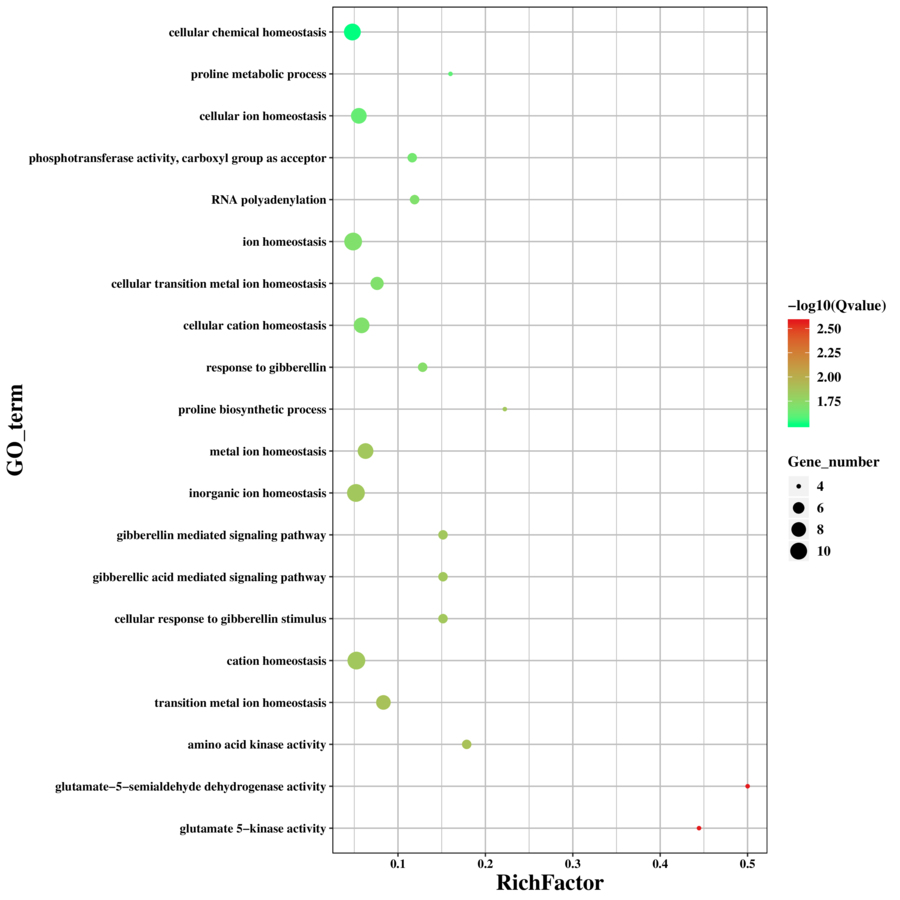


**Fig S23** GO enrichment result of DEGs belonging to Subcluster 17 (Fig. S20) in roots.

**
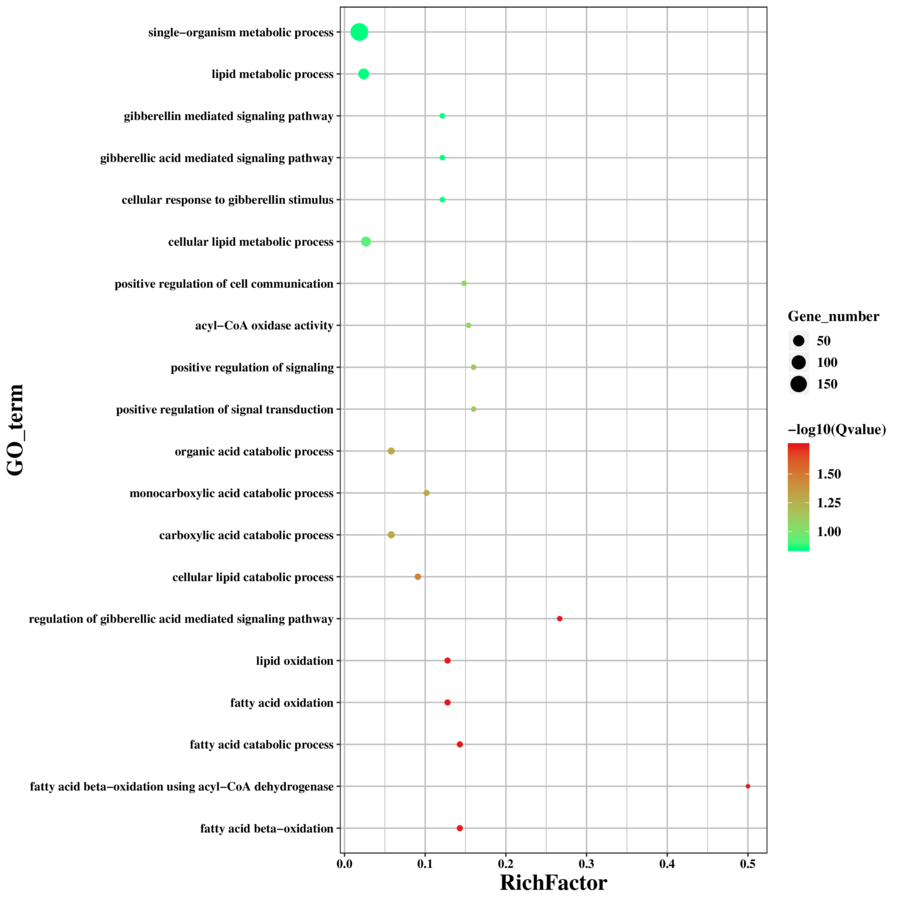
**

**Fig S24** GO enrichment result of DEGs belonging to Subcluster 1 (Fig. S19) in leaves.

**
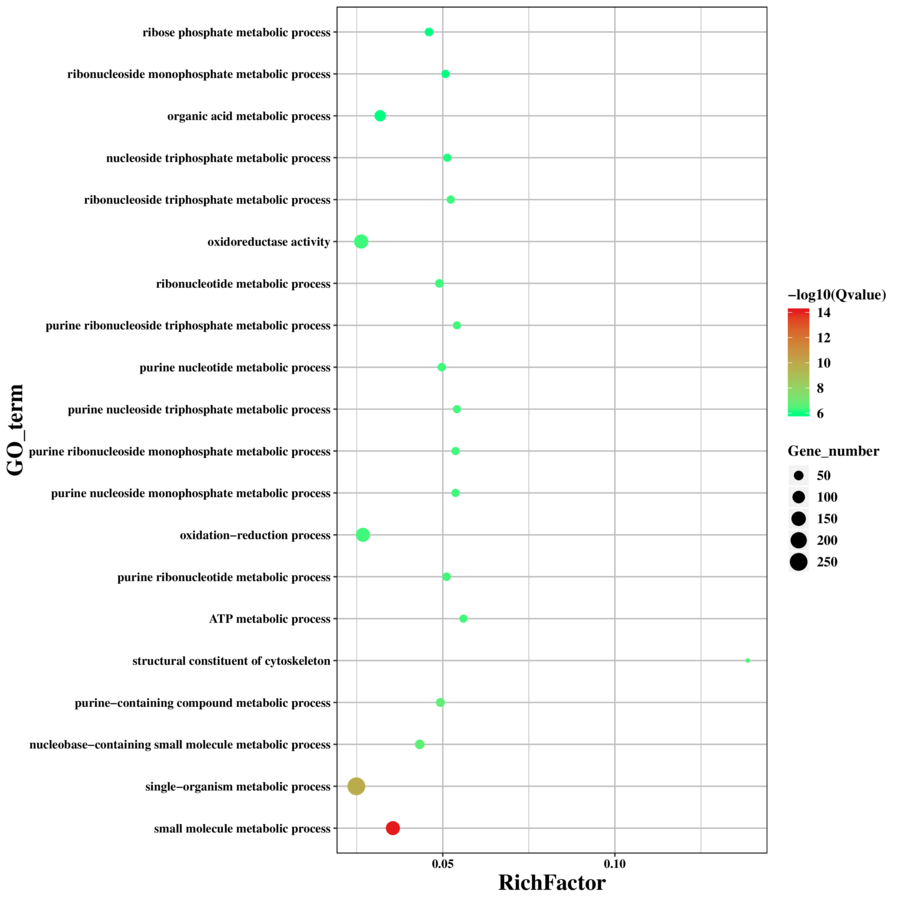
**

**Fig S25** GO enrichment result of DEGs belonging to Subcluster 9 (Fig. S20) in roots.


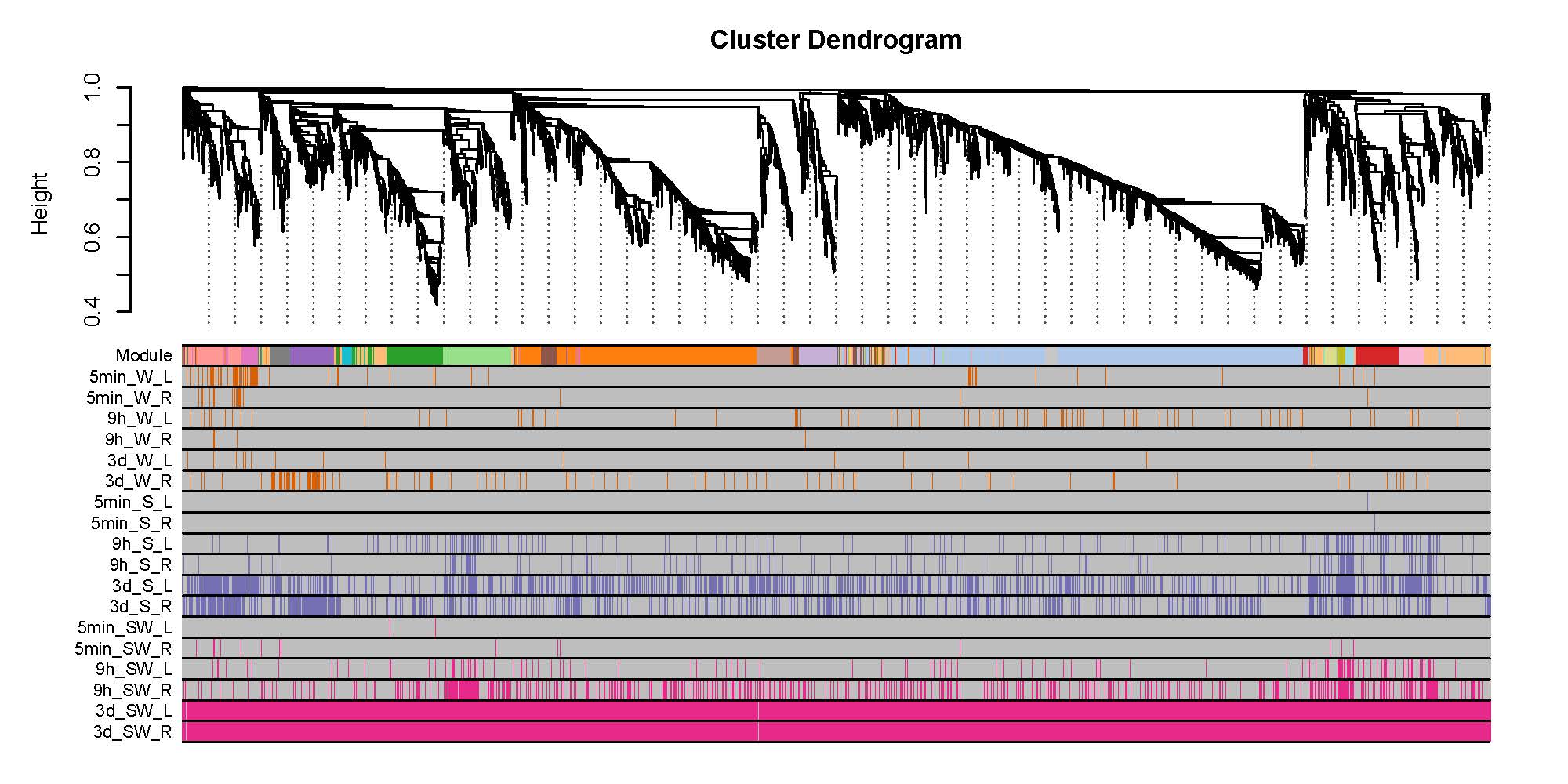


**Fig S26** The expressed genes under SW treatment were classified into twenty modules.


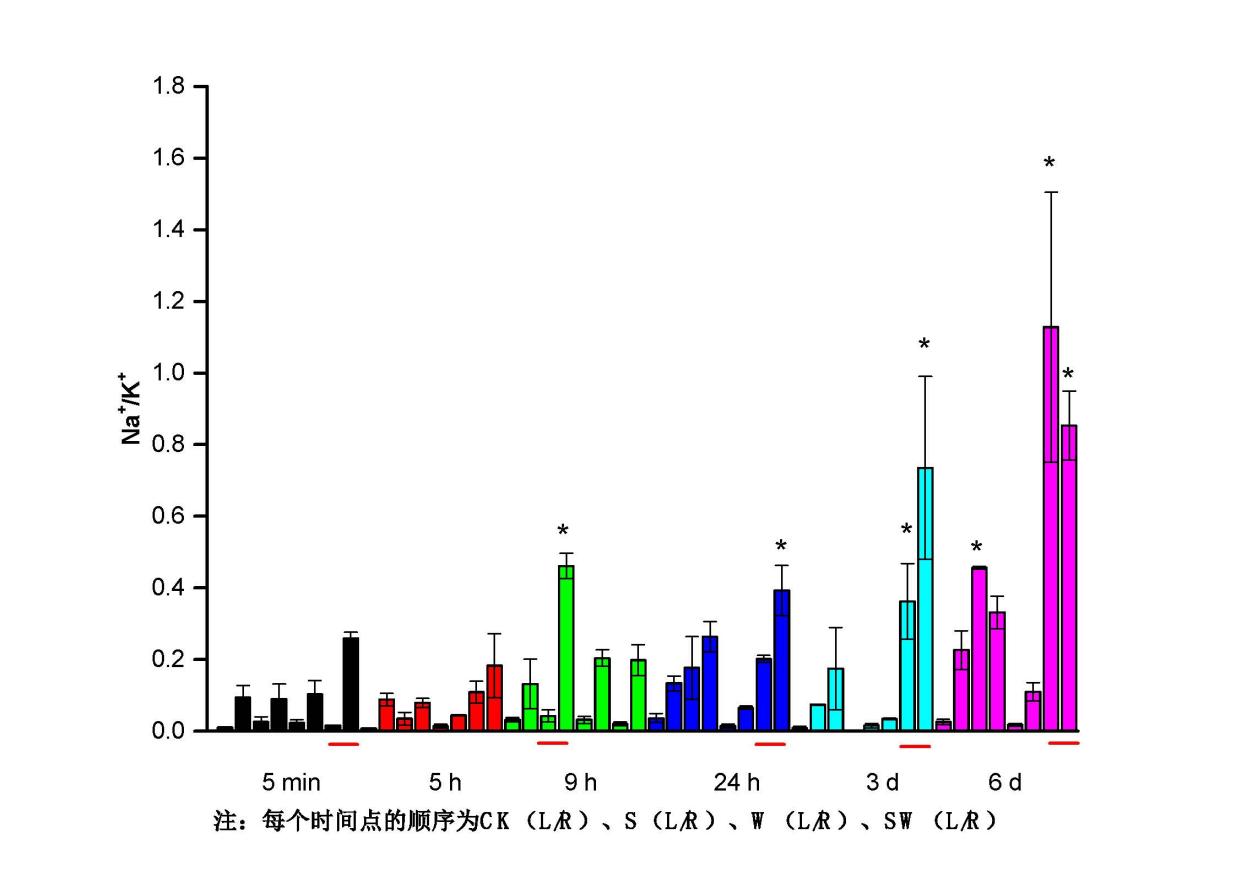


**Fig S27** Na^+^/K^+^ ratio in different period of root and leaf under treatment.

Different colors represented different timepoints：black (5 min), red (5 h), green (9 h), dark blue (24 h), light blue (3 d), pink (6 d). Eight columns at each timepoint represented 8 treatments, in the order of: CK (leaf), CK (root), S (leaf), S (root), W (leaf), W (root), SW (leaf), SW (root). The red horizontal line at the bottom indicates that there is a significant difference in Na^+^/K^+^ between leaves and roots, and asterisk indicates that there is a significant difference between treatment and control

## Supplementary Information Tables

**Table S1** Statistics of Number of molecules in ZMW pore

| **Sample** | **Cell Well** | **Productive ZMW** | **Productive 0(%)** | **Productive 1(%)** | **Productive 2(%)** |
| --- | --- | --- | --- | --- | --- |
| sample 1 | A01 | 1,014,246 | 28.82 | 60.77 | 10.90 |
| sample 2 | A01 | 1,012,230 | 36.97 | 56.16 | 7.56 |
| sample 3 | B01 | 1,014,618 | 35.33 | 58.81 | 6.32 |
| sample 4 | C01 | 1,015,446 | 53.96 | 42.02 | 4.39 |
| sample 5 | D01 | 1,015,195 | 26.44 | 65.27 | 8.69 |
| sample 6 | E01 | 1,015,261 | 32.80 | 61.80 | 5.79 |
| sample 7 | F01 | 1,014,426 | 38.45 | 57.64 | 4.39 |
| sample 8 | E01 | 1,017,916 | 30.79 | 63.44 | 5.89 |
| sample 9 | A01 | 1,008,481 | 28.04 | 66.65 | 6.37 |
| sample 10 | B01 | 1,011,382 | 34.54 | 60.11 | 6.13 |
| sample 11 | C01 | 1,008,416 | 24.75 | 70.06 | 6.26 |
| sample 12 | D01 | 1,010,012 | 23.56 | 70.61 | 6.74 |
| sample 13 | E01 | 1,012,339 | 16.16 | 75.64 | 8.88 |

**Table S2** Statistics of Sample sequencing data quality

| **Sample** | **Polymerase read bases (bp)** | **Polymerase read number** | **Average polymerase read length (bp)** | **Polymerase read N50 (bp)** | **Average Longest Subread length (bp)** |
| --- | --- | --- | --- | --- | --- |
| sample 1 | 10,150,907,383 | 611,401 | 16,603 | 28,750 | 10,017 |
| sample 2 | 10,594,983,060 | 561,514 | 18,869 | 35,750 | 9,652 |
| sample 3 | 10,884,919,293 | 592,093 | 18,384 | 33,750 | 10,051 |
| sample 4 | 8,096,436,497 | 422,892 | 19,145 | 35,250 | 10,340 |
| sample 5 | 11,257,056,661 | 658,577 | 17,093 | 31,250 | 9,587 |
| sample 6 | 10,881,586,640 | 623,408 | 17,455 | 31,750 | 10,547 |
| sample 7 | 11,079,307,262 | 579,862 | 19,107 | 35,250 | 10,826 |
| sample 8 | 9,999,872,206 | 644,467 | 15,516 | 26,250 | 10,739 |
| sample 9 | 11,075,363,198 | 661,425 | 16,745 | 31,250 | 10,259 |
| sample 10 | 8,938,749,938 | 600,049 | 14,897 | 25,250 | 9,966 |
| sample 11 | 11,946,988,318 | 695,652 | 17,174 | 32,250 | 10,277 |
| sample 12 | 11,268,057,421 | 703,972 | 16,006 | 29,250 | 10,124 |
| sample 13 | 11,885,856,424 | 758,796 | 15,664 | 28,750 | 9,888 |
| total | 138,060,084,301 | 8,114,108 |  |  |  |

**Table S3** Statistics of Subread

| **Sample** | **Total bases (bp)** | **Subread number** | **Average subread length (bp)** |
| --- | --- | --- | --- |
| sample 1 | 10,116,966,441 | 1,360,198 | 7,438 |
| sample 2 | 10,546,042,481 | 1,664,727 | 6,335 |
| sample 3 | 10,845,058,720 | 1,487,724 | 7,290 |
| sample 4 | 8,065,970,956 | 1,107,828 | 7,281 |
| sample 5 | 11,214,779,017 | 1,598,258 | 7,017 |
| sample 6 | 10,849,586,355 | 1,326,858 | 8,177 |
| sample 7 | 11,043,446,660 | 1,370,443 | 8,058 |
| sample 8 | 9,978,196,214 | 1,090,120 | 9,153 |
| sample 9 | 11,041,004,812 | 1,397,920 | 7,898 |
| sample 10 | 8,915,647,072 | 1,087,280 | 8,200 |
| sample 11 | 11,910,711,147 | 1,479,311 | 8,052 |
| sample 12 | 11,237,147,431 | 1,369,954 | 8,203 |
| sample 13 | 11,853,809,340 | 1,459,497 | 8,122 |
| total | 137,618,366,646 | 17,800,118 |  |

**Table S4** Statistics for the *H. hamabo* assembly

| **Name** | **scaffold length (bp)** | **scaffold number** | **contig length (bp)** | **contig number** |
| --- | --- | --- | --- | --- |
| max_len | 64,458,935 | -- | 9,364,180 | -- |
| N10 | 58,974,770 | 3 | 3,797,336 | 36 |
| N20 | 52,190,993 | 6 | 3,025,233 | 86 |
| N30 | 41,980,257 | 10 | 2,423,272 | 150 |
| N40 | 37,773,810 | 14 | 1,969,863 | 229 |
| N50 | 36,349,750 | 19 | 1,645,376 | 324 |
| N60 | 32,975,219 | 24 | 1,281,892 | 442 |
| N70 | 32,173,236 | 29 | 999,920 | 594 |
| N80 | 29,615,882 | 35 | 711,915 | 794 |
| N90 | 28,208,848 | 41 | 386,045 | 1,113 |
| Total_length | 1,718,145,230 | 1,915 | 1,716,923,730 | 4,358 |
| number>=2,000bp | -- | 1,496 | -- | 3,939 |

**Table S5** Statistics for base composition

|  | **Length (bp)** | **% of genome** |
| --- | --- | --- |
| A | 558,672,331 | 32.52 |
| C | 299,815,878 | 17.45 |
| G | 299,773,058 | 17.45 |
| T | 558,662,463 | 32.52 |
| N | 1,221,500 | 0.07 |
| GC | 599,588,936 | 34.90 |
| Total | 1,718,145,230 | 100.00 |

Note: The genome size used to calculate GC content does not contain N

**Table S6** Statistics for mapping result

| **Reads mapping rate (%)** | **base mapping rate (%)** | **Average sequencing depth** | **Coverage (%)** | **Coverage (>= 5X, %)** | **Coverage (>= 10X, %)** | **Coverage (>= 20X, %)** |
| --- | --- | --- | --- | --- | --- | --- |
| 91.12 | 96.80 | 80.10 | 99.57 | 98.97 | 98.26 | 97.36 |

**Table S7** Statistics of assembly integrity and sequencing uniformity

| **Reads mapping rate (%)** | **base mapping rate (%)** | **Average sequencing depth** | **Coverage (%)** | **Coverage (>= 5X, %)** | **Coverage (>= 10X, %)** | **Coverage (>= 20X,%)** |
| --- | --- | --- | --- | --- | --- | --- |
| 91.12 | 96.80 | 80.10 | 99.57 | 98.97 | 98.26 | 97.36 |

**Table S8** Statistics of SNP types

| **SNP** | **Number** | **Percentage of SNP (%)** | **Percentage of genome (%)** |
| --- | --- | --- | --- |
| All SNP | 788,866 | 100.0000 | 0.0459 |
| Heterozygosis SNP | 778,038 | 98.6274 | 0.0453 |
| Homology SNP | 10,828 | 1.3726 | 0.0006 |

**Table S9** Statistics of indel types

| **indel** | **Number** | **Percentage of indel (%)** | **Percentage of genome (%)** |
| --- | --- | --- | --- |
| All indel | 292,537 | 100.0000 | 0.0170 |
| Heterozygosis indel | 240,309 | 82.1465 | 0.0140 |
| Homology indel | 52,228 | 17.8535 | 0.0030 |

**Table S9** Statistics of repeat sequence

| **Type** | **Repeat size(bp)** | **% of genome** |
| --- | --- | --- |
| Trf | 79,468,780 | 4.63 |
| Repeatmasker | 251,986,138 | 14.67 |
| Proteinmask | 198,821,317 | 11.57 |
| *De novo* | 825,517,024 | 48.05 |
| Total | 909,200,730 | 52.92 |

**Table S10** Statistics of repeat sequence classification results

|  | **RepeatMasker TEs** | | **RepeatProteinMask TEs** | | ***De novo*** | | **Combined TEs** | |
| --- | --- | --- | --- | --- | --- | --- | --- | --- |
|  | Length | % | Length | % | Length | % | Length | % |
| DNA | 13,637,228 | 0.79 | 120,644 | 0.01 | 26,057,272 | 1.52 | 38,188,814 | 2.22 |
| LINE | 8,829,874 | 0.51 | 189,203 | 0.01 | 16,043,488 | 0.93 | 23,526,104 | 1.37 |
| SINE | 18,643 | 0.00 | 0 | 0.00 | 332,432 | 0.02 | 350,920 | 0.02 |
| LTR | 227,555,469 | 13.24 | 198,512,211 | 11.55 | 774,781,493 | 45.09 | 814,310,642 | 47.39 |
| Satellite | 2,641,781 | 0.15 | 0 | 0.00 | 359,451 | 0.02 | 2,969,251 | 0.17 |
| Simple repeat | 1,732,892 | 0.10 | 0 | 0.00 | 11,156,454 | 0.65 | 12,698,182 | 0.74 |
| Other | 52,775 | 0.00 | 0 | 0.00 | 0 | 0.00 | 52,775 | 0.00 |
| Unknown | 153,035 | 0.01 | 0 | 0.00 | 5,164,896 | 0.30 | 5,317,813 | 0.31 |
| Total | 251,986,138 | 14.67 | 198,821,317 | 11.57 | 814,001,119 | 47.38 | 870,545,413 | 50.67 |

**Table S11** Statistics of gene prediction

| **Gene set** | | **Number** | **Average gene length (bp)** | **Average CDS length (bp)** | **Average exon per gene** | **Average exon length (bp)** | **Average intron length (bp)** |
| --- | --- | --- | --- | --- | --- | --- | --- |
| *De novo* | AUGUSTUS | 106,645 | 3,118.15 | 1,211.63 | 4.90 | 247.20 | 488.66 |
|  | GlimmerHMM | 133,110 | 1,643.02 | 806.06 | 3.39 | 237.52 | 349.66 |
| Homolog | *C. olitorius* | 104,853 | 2,544.16 | 888.04 | 3.77 | 235.27 | 596.91 |
|  | *G. arboreum* | 119,127 | 3,298.40 | 956.65 | 3.92 | 243.98 | 801.69 |
|  | *G. hirsutum* | 126,663 | 3,623.42 | 933.84 | 3.81 | 244.87 | 955.89 |
|  | *G. raimondii* | 124,758 | 3,668.48 | 945.66 | 3.88 | 243.74 | 945.50 |
|  | *T. cacao* | 111,776 | 2,908.54 | 965.87 | 4.00 | 241.19 | 646.57 |
| trans.orf/ISOseq | | 40,295 | 2,670.38 | 782.07 | 4.11 | 344.73 | 404.03 |
| BUSCO | | 2,148 | 4,651.80 | 1,549.24 | 8.79 | 176.26 | 398.30 |
| MAKER | | 91,869 | 2,750.07 | 1,084.46 | 4.78 | 263.85 | 394.69 |
| HiCESAP | | 107,309 | 2,582.43 | 1,008.86 | 4.42 | 272.00 | 404.24 |

Note: Species used for comparison: *Corchorus olitorius, Gossypium hirsutum, Gossypium arboreum, Gossypium raimondii* and *Theobroma cacao*.

**Table S12** The statistical results of the final gene set evidence

|  | **>=20% overlap** | | | **>=50% overlap** | | **>=80% overlap** |
| --- | --- | --- | --- | --- | --- | --- |
|  | No. | Ratio (%) | No. | Ratio (%) | No. | Ratio (%) |
| P(single) | 316 | 0.29 | 1,735 | 1.62 | 11,193 | 10.43 |
| P(more) | 10,345 | 9.64 | 12,038 | 11.22 | 8,931 | 8.32 |
| H(single) | 282 | 0.26 | 353 | 0.33 | 548 | 0.51 |
| H(more) | 789 | 0.74 | 924 | 0.86 | 1,389 | 1.29 |
| C(single) | 4,914 | 4.58 | 5,474 | 5.10 | 6,508 | 6.06 |
| C(more) | 0 | 0.00 | 0 | 0.00 | 0 | 0.00 |
| PH | 33,654 | 31.36 | 35,129 | 32.74 | 35,284 | 32.88 |
| PC | 3,761 | 3.50 | 3,476 | 3.24 | 3,534 | 3.29 |
| HC | 1,704 | 1.59 | 2,007 | 1.87 | 3,241 | 3.02 |
| PHC | 51,544 | 48.03 | 46,150 | 43.01 | 36,552 | 34.06 |

Note:

(1) P: is supported by the results of De Novo's prediction.

(2) P(single): This gene was supported by only one De novo predictive software.

(3) P(more): here are more than two DE NOVO prediction software support at the same time, no H and C support.

(4) H: indicates evidence supported by homologous prediction data.

(5) H(single): The gene was supported by only one Homolog species.

(6) H(More): The gene was supported by multiple Homolog species, but not by P and C.

(7) C: means there is evidence of RNA-seq support.

(8) C(SINGLE): RNASEQ or ISSEQ only.

(9) C(more): RNA-Seq and ISSEQ are both supported, but P and H are not.

(10) PH: only Denovo and homologous simultaneously supported.

(11) PC: only Denovo and transcription are supported at the same time.

(12) HC: Only Denovo and homologous support.

(13) PHC: simultaneously supported by DENOVO, homology and transcription.

(14) Overlap: refers to the ratio of the overlap between the final gene set and the CDS region of various predicted results.

**Table S13** Statistics of function annotation

| **Type** | | **Number** | **Percentage (%)** |
| --- | --- | --- | --- |
| Total | | 107,309 |  |
| Annotated | InterPro | 72,709 | 67.76 |
|  | GO | 50,042 | 46.63 |
|  | KEGG_ALL | 96,295 | 89.74 |
|  | KEGG_KO | 35,671 | 33.24 |
|  | Swissprot | 70,593 | 65.78 |
|  | TrEMBL | 96,377 | 89.81 |
|  | NR | 96,701 | 90.11 |
| Annotated | | 96,981 | 90.38 |
| Unannotated | | 10,328 | 9.62 |

**Table S14** Statistics of non-coding RNA annotations

| **Type** | | **Copy** | **Average length(bp)** | **Total length(bp)** | **% of genome** |
| --- | --- | --- | --- | --- | --- |
| miRNA | | 439 | 122.09 | 53,598 | 0.0031 |
| tRNA | | 2,743 | 75.22 | 206,328 | 0.0120 |
| rRNA | rRNA | 3,936 | 585.92 | 2,306,169 | 0.1342 |
|  | 18S | 1,039 | 1,716.72 | 1,783,671 | 0.1038 |
|  | 28S | 1,518 | 199.72 | 303,173 | 0.0176 |
|  | 5.8S | 990 | 175.33 | 173,575 | 0.0101 |
|  | 5S | 389 | 117.61 | 45,750 | 0.0027 |
|  | 8S | 0 | 0.00 | 0 | 0.0000 |
| snRNA | snRNA | 583 | 126.54 | 73,772 | 0.0043 |
|  | CD-box | 288 | 107.20 | 30,875 | 0.0018 |
|  | HACA-box | 46 | 128.28 | 5,901 | 0.0003 |
|  | splicing | 249 | 148.58 | 36,996 | 0.0022 |
|  | scaRNA | 0 | 0.00 | 0 | 0.0000 |

**Table S15** Sample grouping information for transcriptome

| **Group number** | **Samples in the group** | | |  |
| --- | --- | --- | --- | --- |
| A (5 min-CK-L) | A1 | A2 | A3 | |
| B (5 min-CK-R) | B1 | B2 | B3 | |
| C (5 min-S-L) | C1 | C2 | C3 | |
| D (5 min-S-R) | D1 | D2 | D3 | |
| E (5 min-W-L) | E1 | E2 | E3 | |
| F (5 min-W-R) | F1 | F2 | F3 | |
| G (5 min-SW-L) | G1 | G2 | G3 | |
| H (5 min-SW-R) | H1 | H2 | H3 | |
| I (9 h-CK-L) | I1 | I2 | I3 | |
| J (9 h-CK-R) | J1 | J2 | J3 | |
| K (9 h-S-L) | K1 | K2 | K3 | |
| L (9 h-S-R) | L1 | L2 | L3 | |
| M (9 h-W-L) | M1 | M2 | M3 | |
| N (9 h-W-R) | N1 | N2 | N3 | |
| O (9 h-SW-L) | O1 | O2 | O3 | |
| P (9 h-SW-R) | P1 | P2 | P3 | |
| Q (3 d-CK-L) | Q1 | Q2 | Q3 | |
| R (3 d-CK-R) | R1 | R2 | R3 | |
| S (3 d-S-L) | S1 | S2 | S3 | |
| T (3 d-S-R) | T1 | T2 | T3 | |
| U (3 d-W-L) | U1 | U2 | U3 | |
| V (3 d-W-R) | V1 | V2 | V3 | |
| W (3 d-SW-L) | W1 | W2 | W3 | |
| X (3 d-SW-R) | X1 | X2 | X3 | |

**NOTE:**

**Time** 5 min, 9 h, 3 d

**Treatment** CK (control), S (3.5% NaCl solution), W (waterlogging), SW (NaCl+ waterlogging)

**Organs** L(leaf), R(root)
